# Supplementary material for: Examining the Intended and Unintended Impacts of Raising a Minimum Legal Drinking Age on Primary and Secondary Societal Harm and Violence from a Contextual Policy Perspective: A Scoping Review
Source: Int J Environ Res Public Health. 2021 Feb 19;18(4):1999. doi: 10.3390/ijerph18041999 (PMC7922690; doi:10.3390/ijerph18041999)
Supplement: Supplementary file 1 [file ijerph-18-01999-s001.pdf]

Supplementary file S1 - final scientific search strategy

Web of Science

1st strategy

TOPIC: (minor* OR adolescent* OR teenager* OR youth* OR underage*) AND TOPIC: (alcohol*) AND TOPIC: (drinking* OR buying* OR purchasing* OR possessing* OR selling* OR vending* OR “underage drinking”) AND TOPIC: (“age law” OR “legal restriction” OR “age restriction” OR “age limit” OR “legal age limit” OR “minimum legal drinking age” OR “MLDA” OR “legal drinking age” OR “minimum drinking age” OR “drinking age” OR “purchasing age” OR “age restriction”) AND TOPIC: (policy* OR legislation* OR regulation*) AND TOPIC: (increase* OR raise* OR change*)

2st strategy

TOPIC: (((“age law” OR “legal restriction” OR “age restriction” OR “age limit” OR “legal age limit” OR “minimum legal drinking age” OR “MLDA” OR “legal drinking age” OR “minimum drinking age” OR “drinking age” OR “purchasing age” OR “age restriction”) NEAR/10 increase*))
Refined by: TOPIC: (alcohol*) AND TOPIC: (drinking*)
TOPIC: ((("age law" OR "legal restriction" OR "age restriction" OR "age limit" OR "legal age limit" OR "minimum legal drinking age" OR "MLDA" OR "legal drinking age" OR "minimum drinking age" OR "drinking age" OR "purchasing age" OR "age restriction") NEAR/10 raise*))
Refined by: TOPIC: (alcohol*) AND TOPIC: (drinking*)
TOPIC: ((("age law" OR "legal restriction" OR "age restriction" OR "age limit" OR "legal age limit" OR "minimum legal drinking age" OR "MLDA" OR "legal drinking age" OR "minimum drinking age" OR "drinking age" OR "purchasing age" OR "age restriction") NEAR/10 change*))
Refined by: TOPIC: (alcohol*) AND TOPIC: (drinking*)

Sociological abstracts

1st strategy

MAINSUBJECT("underage drinking") AND (increase* OR raise* OR change*)

2st strategy

(minor* OR adolescent* OR teenager* OR youth* OR underage*) AND alcohol* AND (drinking* OR buying* OR purchasing* OR possessing* OR selling* OR vending* OR "underage drinking") AND (“age law” OR “legal restriction” OR “age restriction” OR “age limit” OR “legal age limit” OR “minimum legal drinking age” OR “MLDA” OR “legal drinking age” OR “minimum drinking age” OR “drinking age” OR “purchasing age” OR “age restriction”) AND (policy* OR legislation* OR regulation*) AND (increase* OR raise* OR change*)

3rd strategy

((“age law” OR “legal restriction” OR “age restriction” OR “age limit” OR “legal age limit” OR “minimum legal drinking age” OR “MLDA” OR “legal drinking age” OR “minimum drinking age” OR “drinking age” OR “purchasing age” OR “age restriction”) N/10 (increase* OR raise* OR change*)) AND (alcohol* or drinking*)

PubMed

1st strategy

((("Underage Drinking"[Mesh]) AND ("Underage Drinking/epidemiology"[Mesh] OR "Underage Drinking/legislation and jurisprudence"[Mesh] OR "Underage Drinking/organization and administration"[Mesh] OR "Underage Drinking/prevention and control"[Mesh] OR "Underage Drinking/trends"[Mesh]))) AND (increase* OR raise* OR change*)

2st strategy

((((((minor* OR adolescent* OR teenager* OR youth* OR underage*)) AND alcohol*) AND (drinking* OR buying* OR purchasing* OR possessing* OR selling* OR vending* OR “underage drinking”)) AND (“age law” OR “legal restriction” OR “age restriction” OR “age limit” OR “legal age limit” OR “minimum legal drinking age” OR “MLDA” OR “legal drinking age” OR “minimum drinking age” OR “drinking age”

OR “purchasing age” OR “age restriction”)) AND (policy* OR legislation* OR regulation*)) AND (increase* OR raise* OR change*)

PsycINFO

1st strategy

(MA underage drinking AND (legislation or laws or regulation or policy or prevention or epidemiology or trends)) AND (increase* or raise* or change*)

2st strategy

(minor* OR adolescent* OR teenager* OR youth* OR underage*) AND alcohol* AND (drinking* OR buying* OR purchasing* OR possessing* OR selling* OR vending* OR “underage drinking”) AND (“age law” OR “legal restriction” OR “age restriction” OR “age limit” OR “legal age limit” OR “minimum legal drinking age” OR “MLDA” OR “legal drinking age” OR “minimum drinking age” OR “drinking age” OR “purchasing age” OR “age restriction”) AND (policy* OR legislation* OR regulation*) AND (increase* OR raise* OR change*)

3rd strategy

((“age law” OR “legal restriction” OR “age restriction” OR “age limit” OR “legal age limit” OR “minimum legal drinking age” OR “MLDA” OR “legal drinking age” OR “minimum drinking age” OR “drinking age” OR “purchasing age” OR “age restriction”) N10 (increase* OR raise* OR change*)) AND (alcohol* or drinking*)

Embase

1st strategy

'alcohol abuse'/exp AND (‘age law’ OR ‘legal restriction’ OR ‘age restriction’ OR ‘age limit’ OR ‘legal age limit’ OR ‘minimum legal drinking age’ OR ‘MLDA’ OR ‘legal drinking age’ OR ‘minimum drinking age’ OR ‘drinking age’ OR ‘purchasing age’ OR ‘age restriction’) AND (minor* OR adolescent* OR teenager* OR youth* OR underage*)

2st strategy

(minor* OR adolescent* OR teenager* OR youth* OR underage*) AND alcohol* AND (drinking* OR buying* OR purchasing* OR possessing* OR selling* OR vending* OR 'underage drinking') AND (‘age law’ OR ‘legal restriction’ OR ‘age restriction’ OR ‘age limit’ OR ‘legal age limit’ OR ‘minimum legal drinking age’ OR ‘MLDA’ OR ‘legal drinking age’ OR ‘minimum drinking age’ OR ‘drinking age’ OR ‘purchasing age’ OR ‘age restriction’) AND (policy* OR legislation* OR regulation*) AND (increase* OR raise* OR change*)

3rd strategy

(('age law' OR 'legal restriction' OR 'age limit' OR 'legal age limit' OR 'minimum legal drinking age' OR 'mlda' OR 'legal drinking age' OR 'minimum drinking age' OR 'drinking age' OR 'purchasing age' OR 'age restriction') NEAR/10 (increase* OR raise* OR change*)) AND alcohol*

Supplementary file S2 - final grey search strategy

OAIster

1st strategy (English language)

((alcohol OR drinking) AND (minor OR minors OR adolescent OR adolescents OR teen OR teenage OR teenager OR teenagers OR youth OR youths OR underage OR underaged) AND (“age law” OR “legal restriction” OR “age restriction” OR “age limit” OR “legal age limit” OR “minimum legal drinking age” OR “MLDA” OR “legal drinking age” OR “minimum drinking age” OR “drinking age” OR “purchasing age” OR “age restriction”))

2st strategy (Dutch language)

((leeftijdsgrens OR leeftijdsgrenzen OR “drank en horecawet” OR “drank- en horecawet”) AND (drank OR drinken OR alcohol)

GLIN

1st strategy (English language)

ALL ((alcohol OR drinking) AND (minor OR minors OR adolescent OR adolescents OR teen OR teenage OR teenager OR teenagers OR youth OR youths OR underage OR underaged))

2st strategy (Dutch language)

((leeftijdsgrens OR leeftijdsgrenzen OR “drank en horecawet” OR “drank- en horecawet”) AND (drank OR drinken OR alcohol)
ALL ((alcohol OR drinken OR drank) AND (minderjarig OR minderjarige OR minderjarigen OR adolescent OR adolescenten OR tiener OR tieners OR jongere OR jongeren OR jeugd OR jeugdigen))

Opengrey

1st strategy (English language)

alcohol* AND (minor* OR adolescent* OR teenager* OR youth* OR underage*) lang:"en"

Google Scholar

1st strategy (English language)

("raise AROUND(10)limit" AND “underage drinking”) OR ("limit AROUND(10)raise" AND “underage drinking”)
("increase AROUND(10)limit" AND “underage drinking”) OR ("limit AROUND(10)increase" AND “underage drinking”)
("change AROUND(10)limit" AND “underage drinking”) OR ("limit AROUND(10)change" AND “underage drinking”)
("raise AROUND(10)limit" AND “alcohol availability”) OR ("limit AROUND(10)raise" AND “alcohol availability”)
("increase AROUND(10)limit" AND “alcohol availability”) OR ("limit AROUND(10)increase" AND “alcohol availability”)
("change AROUND(10)limit" AND “alcohol availability”) OR ("limit AROUND(10)change" AND “alcohol availability”)
("raise AROUND(10)restriction" AND “underage drinking”) OR ("restriction AROUND(10)raise" AND “underage drinking”)
("increase AROUND(10)restriction" AND “underage drinking”) OR ("restriction AROUND(10)increase" AND “underage drinking”)
("change AROUND(10)restriction" AND “underage drinking”) OR ("restriction AROUND(10)change" AND “underage drinking”)
("raise AROUND(10)restriction" AND “alcohol availability”) OR ("restriction AROUND(10)raise" AND “alcohol availability”)
("increase AROUND(10)restriction" AND “alcohol availability”) OR ("restriction AROUND(10)increase" AND “alcohol availability”)

("change AROUND(10)restriction" AND “alcohol availability”) OR ("restriction AROUND(10)change" AND “alcohol availability”)

2st strategy (Dutch language)

("verhoging AROUND(10)leeftijdsgrens" AND “alcohol”) OR ("leeftijdsgrens AROUND(10)verhoging" AND “alcohol”)
("verhogen AROUND(10)leeftijdsgrens" AND “alcohol”) OR ("leeftijdsgrens AROUND(10)verhogen" AND “alcohol”)
("ophoging AROUND(10)leeftijdsgrens" AND “alcohol”) OR ("leeftijdsgrens AROUND(10)ophoging" AND “alcohol”)
("ophogen AROUND(10)leeftijdsgrens" AND “alcohol”) OR ("leeftijdsgrens AROUND(10)ophogen" AND “alcohol”)
("verhoging AROUND(10)leeftijdsgrens" AND “beschikbaarheid”) OR ("leeftijdsgrens AROUND(10)verhoging" AND “beschikbaarheid”)
("verhogen AROUND(10)leeftijdsgrens" AND “beschikbaarheid”) OR ("leeftijdsgrens AROUND(10)verhogen" AND “beschikbaarheid”)
("ophoging AROUND(10)leeftijdsgrens" AND “beschikbaarheid”) OR ("leeftijdsgrens AROUND(10)ophoging" AND “beschikbaarheid”)
("ophogen AROUND(10)leeftijdsgrens" AND “beschikbaarheid”) OR ("leeftijdsgrens AROUND(10)ophogen" AND “beschikbaarheid”)
("verhoging AROUND(10)leeftijdsgrens" AND “drank- en horecawet”) OR ("leeftijdsgrens AROUND(10)verhoging" AND “drank- en horecawet”)
("verhogen AROUND(10)leeftijdsgrens" AND “drank- en horecawet”) OR ("leeftijdsgrens AROUND(10)verhogen" AND “drank- en horecawet”)
("ophoging AROUND(10)leeftijdsgrens" AND “drank- en horecawet”) OR ("leeftijdsgrens AROUND(10)ophoging" AND “drank- en horecawet”)
("ophogen AROUND(10)leeftijdsgrens" AND “drank- en horecawet”) OR ("leeftijdsgrens AROUND(10)ophogen" AND “drank- en horecawet”)

Supplementary file S3 - invitation send to experts

Dear KBS member,

My name is Ruud Roodbeen and I am a PhD-student at the department of Tranzo Scientific Centre for Care and Well-being, Tilburg University. For my dissertation, I am currently conducting a scoping review focussing on impact of the raised minimum legal drinking age (MLDA) for the sale and purchase of alcohol. **The aim of our scoping review is to investigate possible intended and unintended (both positive as well as negative) impact of raised MLDA.**

Part of this review is gathering literature (in English or Dutch language) from experts in the field of alcohol research, and for this scoping review to be successful, we really would like your expertise and advice. Therefore, if possible, could you, as an expert in the field of alcohol research, **perhaps indicate relevant literature in English or Dutch language?** Both scientific and grey literature is welcome, describing impact of raised MLDA (intended or unintended). This impact could be on, for example, drinking and/or purchasing behavior of underage youth or adolescents, the attitudes of parents or alcohol sellers, alcohol in relation to traffic accidents, compliance of alcohol sellers, and so on. Some examples of countries with a recently raised MLDA are (obtained from the 'World Health Organization Global status report on alcohol and health 2018' and additional search engines):

- France (in 2009, raised from 16 to 18)
- Denmark (in 2011, raised from 16 to 18)
- Zambia (in 2011, raised from 14 to 18)
- Italy (in 2012, raised from 16 to 18)
- Portugal (in 2013, raised from 16 to 18),
- the Netherlands (in 2014, raised from 16 to 18)
- Malaysia (in 2016, raised from 18 to 21)

Your expertise and advice are very much appreciated, and if applicable, forwarding this email to other experts is very much appreciated as well!

Thanks in advance for your attention and time, we await your reply with much interest. If you have any enquiries regarding our question, our scoping review, my dissertation, or any other questions, please do not hesitate to contact me (using contact details below).

Yours faithfully,

and on behalf of my co-authors, Rachel Dijkstra, Karen Schelleman-Offermans, Roland Friele and Dike van de Mheen,

Ruud Roodbeen

PhD-student Tranzo / AW Verslaving (addiction)

r.t.j.roodbeen@uvt.nl • Secretariat +31 (0)13 466 2969 • Mobile +31 (0)6 46 06 55 27
Tilburg University • Tilburg School of Social and Behavioural Sciences • Tranzo
Mailing address: 90153, 5000 LE Tilburg
Visiting address: Professor Cobbenhagenlaan 125, Tilburg (Reitse Poort 1, kamer RP 234)
www.tilburguniversity.edu/tranzo

Supplementary file S4 - criteria used during selection of studies

In general, studies are excluded when they do not target or are not related to *increases* of minimum legal drinking ages (MLDA). Some examples of this are presented below. Studies are excluded:

1. when they are focused on policy measures or behaviour curbing alcohol availability other than or not directly related to increases of MLDA (e.g., studies only focused on the costs of alcohol, only on taxation effects, or only on changing the blood alcohol concentration (BAC) limit for driving behaviour; for example, [1][2]);
2. when they are only focused on (the effectiveness of) interventions/educational campaigns/programmes not related to MLDA policy (e.g., only examining web-based computer-tailored interventions to prevent binge drinking in adolescence);
3. when they are only addressing epidemiological trends not related to MLDA (e.g., presenting trends in drinking behaviour by age and gender);
4. when investigating the impact of the existing MLDA (e.g., investigating the impact of an existing MLDA of 18 years by comparing drinking behaviour of 16/17-year-olds and 18/19-year-olds within the same region).

Furthermore, studies are excluded:

- when not written in English or Dutch;
- when no full text is available (e.g., conference abstracts or posters);
- when involving MLDA not related to alcohol (e.g., tobacco age limits [3]);
- when targeting the lowering of MLDA (e.g., [4,5]);
- when performed in an area smaller than a state or a (Canadian) province (i.e., areas without their own legislation; e.g., municipalities, counties, residence halls, college campuses, army barracks, native/indigenous lands; e.g., [6]);
- when only targeting a specific subgroup in society (e.g., college students, military, this does not involve specific cohorts of youth or high school students, since they represent all youth in society; e.g., [7,8]);
- when only providing general or context information about MLDAs (e.g., text or reference books, editorials, opinion pieces, reviews from books; e.g., [9,10]);
- when raising an MLDA is only used as a control in analyses (raising an MLDA as a policy measure must have a prominent role in analytic models or at least have a similar role in these models compared to other policy measures; e.g., [11,12]);
- when the impacts of raising an MLDA are based on simulations (e.g., [13,14]);
- when investigating the impact of existing MLDA (i.e., not investigating changes in MLDA, for instance, studies investigating the impact of an existing MLDA of 21 by comparing drinking behaviour of underage youth with adults within the same region to assess effectiveness; e.g., [15,16]);
- when studies only describe methodological protocol (e.g., [17,18]).

We do not require a specific 'increase-amount' (i.e., a specific number of years), a specific type of increase (e.g., an increase for only the sale of alcohol or an increase for only the possession of alcohol), or a specific vendor-type in our search (e.g., increases for bars and cafes or supermarkets and liquor stores), all can be included.

Furthermore, the investigation of a raise in MLDAs always occurs in a natural setting, meaning that multiple policy measures or events exist or are implemented (and sometimes evaluated) simultaneously (e.g., the simultaneous increase of taxes or BAC-levels for driving). Studies that investigated raising an MLDA as one of multiple policy measures are included (and should have a similar role in analytic models compared to other policy measures).

References supplementary file S4

1. Rowland, B.; Toumbourou, J.W.; Livingston, M. The association of alcohol outlet density with illegal underage adolescent purchasing of alcohol. *J. Adolesc. Heal.* **2015**, *56*, 146–152, doi:10.1016/j.jadohealth.2014.08.005.
2. Fell, J.C.; Voas, R.B. The effectiveness of reducing illegal blood alcohol concentration (BAC) limits for driving: Evidence for lowering the limit to .05 BAC. *J. Safety Res.* **2006**, *37*, 233–243, doi:10.1016/j.jsr.2005.07.006.
3. Ertan Yörük, C.; Yörük, B.K. Do minimum legal tobacco purchase age laws work? *Contemp. Econ. Policy* **2016**, *34*, 415–429, doi:10.1111/coep.12153.
4. Kypri, K.; Davie, G.; McElduff, P.; Langley, J.; Connor, J. Long-term effects of lowering the alcohol minimum purchasing age on traffic crash injury rates in New Zealand. *Drug Alcohol Rev.* **2017**, *36*, 178–185, doi:10.1111/dar.12378.
5. Williams, A.F.; Rich, R.F.; Zador, P.L.; Robertson, L.S. The legal minimum drinking age and fatal motor vehicle crashes. *J. Legal Stud.* **1975**, *4*, 219–239, doi:10.1086/467530.
6. Sherman, S.G.; Srirojn, B.; Patel, S.A.; Galai, N.; Sintupat, K.; Limaye, R.J.; Manowanna, S.; Celentano, D.D.; Aramrattana, A. Alcohol consumption among high-risk Thai youth after raising the legal drinking age. *Drug Alcohol Depend.* **2013**, *132*, 290–294, doi:10.1016/j.drugalcdep.2013.02.023.
7. Wallace, A.E.; Wallace, A.; Weeks, W.B. The U.S. military as a natural experiment: Changes in drinking age, military environment, and later alcohol treatment episodes among veterans. *Mil. Med.* **2008**, *173*, 619–625, doi:10.7205/milmed.173.7.619.
8. Keller, A.; Frye, L.; Bauerle, J.; Turner, J.C. Legal ages for purchase and consumption of alcohol and heavy drinking among college students in Canada, Europe, and the United States. *Subst. Abus.* **2009**, *30*, 248–252, doi:10.1080/08897070903041228.
9. Wagenaar, A.C. Aggregate beer and wine consumption. Effects of changes in the minimum legal drinking age and a mandatory beverage container deposit law in Michigan. *J. Stud. Alcohol* **1982**, *43*, 469–487, doi:10.15288/jsa.1982.43.469.
10. Sivan, A.B. Review of alcohol, young drivers, and traffic accidents: Effects of minimum-age laws. *Rehabil. Psychol.* **1984**, *29*, 123–125, doi:10.1037/h0091099.
11. Adrian, M.; Ferguson, B.S.; Her, M. Can alcohol price policies be used to reduce drunk driving? Evidence from Canada. *Subst. Use Misuse* **2001**, *36*, 1923–1957, doi:10.1081/JA-100108433.
12. Houston, D.J.; Richardson, L.E.; Neeley, G.W. Mandatory seat belt laws in the states: A study of fatal and severe occupant injuries. *Eval. Rev.* **1996**, *20*, 146–159, doi:10.1177/0193841X9602000202.
13. Cobiac, L.; Vos, T.; Doran, C.; Wallace, A. Cost-effectiveness of interventions to prevent alcohol-related disease and injury in Australia. *Addiction* **2009**, *104*, 1646–1655, doi:10.1111/j.1360-0443.2009.02708.x.
14. Holm, A.L.; Veerman, L.; Cobiac, L.; Ekholm, O.; Diderichsen, F. Cost-effectiveness of preventive interventions to reduce alcohol consumption in Denmark. *PLoS One* **2014**, *9*, doi:10.1371/journal.pone.0088041.
15. Rooney, J.F.; Schwartz, S. The effect of minimum drinking age laws upon adolescent alcohol use and problems. *Contemp. Drug Probl.* **1977**, *6*, 569–583.
16. National Highway Traffic Safety Administration. *Evaluation of Minimum Drinking Age Laws Using the National Electronic Injury Surveillance System*; Bureau of Transportation Statistics: Washington, DC, USA, 1982.
17. Baccini, M.; Carreras, G. Analyzing the effect of selected control policy measures and socio demographic factors on alcoholic beverage consumption in Europe within the AMPHORA project: Statistical methods. *Subst. Use Misuse* **2014**, *49*, 1546–1554, doi:10.3109/10826084.2014.913388.
18. Kindelberger, J. *Calculating Lives Saved Due to Minimum Drinking Age Laws;* Citeseer: Princeton, NJ, USA, 2005.

Supplementary file S5 - extraction

First path: Implementation

[44]	First author, year, country	General aim	Study design	The policy measure(s) or intervention(s) at hand	Measured policy effects on MLDA	Reflection on policy and other occurrences by authors	Comments
	Hingson, 1983, USA.	- This paper examines the impact of raising the drinking age on teenage drinking, driving after drinking, and non-fatal accident involvement in Massachusetts prior to the law's enactment and twice at yearly intervals after the law enactment.<br>- Massachusetts was compared with New York State, who retained an 18-year-old drinking age.	- Random telephone surveys with approximately 1,000 16-19-year-olds in each state were undertaken prior to the law's enactment and twice at yearly intervals after the law, to assess the law's impact on teenage drinking, driving after drinking, and non-fatal accident involvement.<br>- Respondents were asked where they most often obtained their alcohol (e.g., liquor/grocery store, bar/club, at home) and where they drank > 5 consumptions in the last month (e.g., party, bar).<br>- Drinking was measured asking for ‘any drinking’ in the last month and ‘drinking 6+ drinks at one time’ in the last month.<br>- Log-linear analysis was used on the survey data.<br>- Fatal crash data reported to the US Department of Transportation by each state from 1976-1981 were also analyzed.<br>- To assess law enforcement practices and problems, interviews were conducted with over 50 Massachusetts police officers representing all levels of command in urban, rural, and suburban jurisdictions.	- Legislation raising the legal drinking age in Massachusetts from 18 to 20 in 1979.<br>- At the time Massachusetts raised its legal drinking age from 18 to 20, the two states had similar laws regarding age of driving licensure and penalties for driving while intoxicated.	- After the law, the frequency of teenage drinking in bars and clubs and the percentage of teenagers reporting they most often obtained their alcohol in liquor stores and groceries dropped in Massachusetts compared to New York. (sig.).<br>- During the two years after the law, the proportion of Massachusetts teenagers who had someone else purchase alcohol for them or who most often obtained alcohol from their homes nearly doubled (sig.).<br>- The average daily consumption of alcohol in the 16-19-year-old age group during the two years after the law did not decline in Massachusetts compared to New York. Nor did teenagers report shifts to the use of other psychoactive drugs.<br>- After the law, the proportion of teenagers who reported driving after drinking heavily (six or more drinks) did not decline in either state. However, the frequency that teenagers reported they drove after any drinking declined significantly more in Massachusetts.<br>- The three methods of statistical analysis indicated no significant difference between the two states in the overall teenage fatal accident trends (not sig.).<br>- The frequency with which teenagers were arrested for driving under the influence did not significantly change in Massachusetts during the first year after the law compared to the previous two years.<br>- Enforcement of the law focusing on the sellers was minimal and sporadic.<br>- The intensity of enforcement varied widely from community to community.<br>- The reasons most often cited for the variability in enforcement of the law among communities across the state was the lack of personnel and competing priorities, particularly in some high crime inner-city jurisdictions. Moreover, many officers did not perceive teenage purchasing of alcohol or drinking per se as a sufficiently serious crime to stigmatize<br>Juveniles by putting an arrest on their records.	- The study examined the first two years following enactment of the law. During this time period, the 18 and 19-year-old age groups who had previously been allowed to drink had that privilege revoked. One could hypothesize that the previous drinking habits of this group would be resistant to change.<br>- The state's law provides a symbolic statement to teenagers that its citizens disapprove of their drinking, and fears the accidents they may cause when they drive after drinking. The study results prompt us to ask whether the law could have had a greater impact among all Massachusetts teenagers if enforcement efforts were more consistent and if greater attention were paid to preventing the common practices of non-teenagers purchasing alcohol for teenagers or liquor outlets not requiring age identification. Without sufficient resources and coordination of enforcement efforts, those police who actively strive to enforce the law in one community may find their efforts negated by minimal enforcement in the next.<br>- Lack of community resources and variable willingness to enforce laws focused on teenagers raise questions about whether alternative strategies such as increased enforcement of the drunk driving and traffic safety laws aimed at all drivers, or requirements for safer cars and improved road design would yield greater reductions in nonfatal and fatal accidents both among teenagers and non-teenagers.	**INCLUDE.**<br><br>- In addition to telephone surveys, data from the Fatality Analysis Reporting System (FARS) was used.

[45]	First author, year, country	General aim	Study design	The policy measure(s) or intervention(s) at hand	Measured policy effects on MLDA	Reflection on policy and other occurrences by authors	Comments
	Vingilis, 1981, Canada.	This paper reports four types of data which are relevant to the study of the effects of the increase in the MLDA.	Data are from:<br>1) surveys of drinking and drinking problems among high school students (measuring: 1) alcohol use in the month prior, 2) feeling 'tight' at least once in the month prior, 3) 'drunk' at least once in the month prior, 4) 5 or more drinks on a singes occasion at least once in the month prior). Conducted in 1977 and 1979, stratified proportional sample in the province. Log linear analyses.<br>2) a study (surveys) of perceptions of vice-principals. Conducted in 1972 and 1980.<br>3) a trend analyses of young drinking offender charges; and<br>4) trend analyses of drinking-driving statistics and driver fatalities. Two types of drinking-driving statistics were obtained: monthly Ontario drinking-driving convictions form 1977 and 1979 for 16-21-year-olds and monthly Ontario accident fatalities from 1973 to 1979 for 16-21-year-olds. Time series analyses were used.	The increase of the MLDA in 1979 in Ontario (from 18 to 19).	1) the proportion of drinkers among 18-19-year-olds decreased from 1977 to 1979 (as well as a decrease in the proportion feeling 'tight'). Comparable effects were observed for younger drinkers (under 18) in the exact opposite direction.<br>2) The findings from this study indicate that the vice-principals have perceived either no change or less student drinking and alcohol-related problems at their school since the increase in legal drinking age (between 20 per cent and 30 per cent of the vice-principals reported a decrease in drink-related behaviors).<br>4) There were no significant differences between the pre-intervention and post-intervention time periods for both types of drinking-driving statistics.	- The results of the four studies indicate few statistically significant changes, however, these findings seem to tentatively suggest a minimal effect for 18-19-year-old high school students, but not for the regular (once a week or more) or younger drinkers.<br>- The fact that regular drinkers reported no changes in their drinking habits could partially explain why major changes were not observed for the more general methods of measurement, that is, the rates of accidents, charges and convictions and the perceptions of vice-principals.<br>- Additionally, a large proportion of Ontario youths are not in school and these non-students may be heavier drinkers. Conviction and fatality statistics are not sensitive enough measures, as it seems that too few high-accident-and-arrest-risk youths were changing their drinking patterns for an effect to emerge from these statistics.<br>- The minimal Ontario effect was expected, as it was felt that the one-year increase was not sufficient to cause a major impact on youthful drinking behavior.	INCLUDE.<br><br>- Data from the Fatal Accident Reporting System (FARS).<br><br>- Only 1, 2 and 4 approved criteria and are extracted.

[46]	First author, year, country	General aim	Study design	The policy measure(s) or intervention(s) at hand	Measured policy effects on MLDA	Reflection on policy and other occurrences by authors	Comments
	Fell, 2008, USA.	This study reports on an effort to evaluate and interrelate the existence and strength of two core MLDA laws and fourteen expanded laws designed to (a) control the sales of alcohol, (b) prevent possession and consumption of alcohol, and (c) prevent alcohol impaired driving by youth aged 20 and younger.	- Our first analysis determined if the enactment of the possession and purchase laws (the two core MLDA laws) was associated with a reduction in the ratio of drinking to nondrinking drivers aged 20 and younger who were involved in fatal crashes controlling for as many variables as possible.<br>- Annual FARS data were used from 1982 to 1990. An Analysis of Variance (ANOVA) was used.<br>- Our second analysis determined whether the existence and strength of any of the 16 underage drinking laws was associated with a reduction in the percentage of drivers aged 20 and younger involved in fatal crashes who were drinking. Pooled data from the 1998-2004 FARS data was used. Stepwise Linear Regression models were used.	- Two core MLDA laws (prohibiting possession and purchase of alcohol by youth).<br>- 14 additional underage drinking laws (consumption, furnishing/selling, age 21 for on-premises servers/sellers, age 21 for off-premises servers/sellers, zero tolerance, use and lose, keg registration, RBS training, use of fake ID, transfer/production of fake ID, retailer support provisions for fake ID, social host-underage parties, GDL with night restrictions, state control of alcohol).	- In the presence of numerous covariates, the possession and purchase laws account for an 11.2% ($p = 0.041$) reduction in the ratio of alcohol-positive to alcohol-negative younger than age 21 drivers involved in fatal crashes.<br>- Making it illegal to use a false identification to purchase alcohol was significant (second analysis). Specifically, from state to state, a unit difference (increase) in the strength of the False ID Use law was associated with a 7.3% smaller outcome measure ($p = .034$).	- Differences across states in patterns of underage alcohol use and drinking-related problems may exist that call for varying mixes of legal provisions. Such differences across states in effectiveness of laws could also explain why we found few significant results.<br>- The awareness of these laws by youth and the enforcement of these laws may play a much greater role than the presence, absence or strength (as assessed in this study).<br>- This study did not have the opportunity to uncover the impact of the enforcement of the laws, which may be the most important factor in MLDA effectiveness. Even without substantial enforcement, it may be important for states to adopt effective expanded MLDA 21 laws to have a good foundation in preventing, or at least reducing, underage drinking.	**INCLUDE.**<br><br>- The outcome data - drinking driver and nondrinking driver fatal traffic crashes - were obtained from the Fatality Analysis Reporting System (FARS) maintained by NHTSA.<br>- Information from the National Institute on Alcohol Abuse and Alcoholism (NIAAA) Alcohol Policy Information System (APIS) dataset (1998-2005) was used.<br>- Information from the National Highway Traffic Safety Administration's (NHTSA, 2006) Digest of Impaired Driving and Selected Beverage Control Laws was used.<br>- Information from the Insurance Institute for Highway Safety (IIHS) was used.

[47]	First author, year, country	General aim	Study design	The policy measure(s) or intervention(s) at hand	Measured policy effects on MLDA	Reflection on policy and other occurrences by authors	Comments
	Schelleman-Offermans, 2017, the Netherlands.	The main aim of this study is to investigate the effect of the planned increase of the minimum legal age for the sale of alcohol in the Netherlands from 16 to 18 years old on the compliance of alcohol retailers (on- and off-premises) using 15-year-old mystery shoppers. In other words, the question is raised whether the compliance with the minimum legal age for selling alcoholic beverages via on-premise (sport bars, public bars, cafe's and disco's) and off-premise outlets (take-away restaurants, supermarkets, liquor stores, and alcohol home delivery services) significantly has increased for 15-year-olds after the minimum legal age was raised.	A total of 1770 alcohol purchase attempts by 15-year-old mystery shoppers were conducted in three independent cross-sectional Dutch representative samples of on- and off-premise alcohol outlets in 2013 (T0), 2014 (T1), and 2016 (T2). The effect of the policy change was estimated controlling for gender and age of the vendor. Univariate analyses (Chi-square) were conducted to explore changes in compliance one and two years after the increased minimum age was introduced. A single logistic regression analysis was conducted to estimate the effect of the policy change on compliance (no/yes) of alcohol sellers, controlling for the passage of time (T1, T2, with T0 as reference).	The increase from 16 to 18 years of the MLDA for the sale and purchase of all alcoholic beverages in the Netherlands in 2014.	- Results showed that vendors requested more frequently an ID (significant overall increase of 7.4% points after one year and 23.3% points after two years) of the 15-year-old mystery shoppers after the policy change.<br>- Mean compliance rates including all alcohol outlets increased significantly by 9.2% points after almost one year and 27.4% points after two years and 5 months compared with before the policy change, even after controlling for the gender and age of the vendor.<br>- Two years after the policy change, alcohol vendors were up to 3 times more likely to comply with the alcohol age limit policy.	- It can be concluded that it became more difficult for 15-year-old adolescents to purchase alcohol after the minimum legal age for alcohol was raised from 16 to 18 years and its effect on compliance seems to increase over time<br>- It might be naïve to believe that the effect found in this study can entirely be attributed to the implementation of the increased minimum legal age for alcohol. Several other changes occurred in the years preceding the raise of the minimum legal age, which might have contributed to the observed effect on compliance, such as the increased media attention or the changes in parental norms considering underage drinking (became stricter).<br>- Effects of the increase in minimum legal age may have been more pronounced if local alcohol policies and enforcement efforts would have been more adjusted to the alcohol policy changes; a process which may take longer than one year. Indeed, after two years, the effect of the new minimum legal age on compliance rates is much greater than after one year.<br>- A rise in the compliance rate was already present in the years preceding the introduction of the new minimum legal age. This perhaps signifies a process in which a lowering in the general acceptability of juvenile drinking already started before the increased minimum legal age was introduced and alcohol vendors might have been anticipating this formal legal change (i.e., policy endogeneity).	**INCLUDE.**<br><br>- In 2013, 1399 alcohol purchase attempts were conducted by 51 mystery shoppers, followed by 361 purchase attempts conducted by 19 different mystery shoppers in 2014, and 398 attempts conducted by 17 again different mystery shoppers in 2016 in on- and off-premise outlets.

[48]	First author, year, country	General aim	Study design	The policy measure(s) or intervention(s) at hand	Measured policy effects on MLDA	Reflection on policy and other occurrences by authors	Comments
	Yu, 1998, USA.	This study examined the perceived change in parental and peer attitudes toward underage drinking associated with the raising of the legal drinking age and its effect on youthful alcohol use and drinking driving.	- Data were collected through telephone interviews (digit-dialing) from randomly selected New Yorkers in the 16 to 20-age groups in 1982 and 1983 and the 16 to 24-age group in 1985 and 1986 (a series of four surveys were conducted in 1982, before the enactment of the 19-drinking age law; 1983, after the enactment of the 19-drinking age law, 1985, before the 21-drinking law; and 1986, after the 21-drinking age law).<br>- Two dependent variables are included: respondents' frequency of drinking in the past month and frequency of driving under the influence of alcohol in the past month.<br>- A three-stage stratified proportionate random sampling procedure was designed. The 57 non-New York City counties were stratified on the dimension of a county's young adult population (16-20 in 82 and 83, 16-24 in 85 and 86) and a county's alcohol crash involvement among those young adults.	New York state raised the legal alcohol drinking age twice in the 1980's, from 18 to 19 in 1982 and from 19 to 21 in 1985.	- The perceived parental approval of alcohol use is much higher among the legal drinkers than illegal drinkers. The change of the approval rate corresponds to the drinking status instead of age: the perceived parental approval rate remained low for illegal drinkers when the legal drinking age changed from 18 to 19 and to 21.<br>- The differences between the perceived peer approval rates for legal and illegal alcohol use are relatively small. The perceived peer approval of alcohol use appears to be independent of the drinking status, the peer approval rate for underage drinking remained close to 80% when the legal drinking age changed from 18 to 21, and the approval rate for legal drinking was a constant 90% for all samples.	- Data show a considerable difference between youths' perceptions of parental and peer attitudes toward youthful alcohol use. Youths are more likely to view their parents as abiding by the drinking age law than their peers. Furthermore, perceived parental attitudes significantly influence alcohol use among the underage drinkers, whereas perceived peer attitudes appear to be effective across all legal and underage samples. These effect patterns correspond to parental and peer attitudes toward alcohol use over the 4-year study period.<br>- Perceived parental approval of alcohol use tends to be specific to the legal drinking age, but perceived peer attitudes tend to be independent of the drinking age laws. Thus, the reduction in youthful drinking and drinking driving may be more attributable to the perception of increased disapproval of teenage alcohol use by parents than to the perceived changes in peer attitudes.<br>- The findings (i.e., differences between perceptions on parents and peers) bear significance for public policies directed at enforcing the 21-drinking age law and reducing youthful alcohol use and highway crashes. Parental involvement should be incorporated as a critical component in the strategies to reduce underage drinking and drinking driving.	**INCLUDE.**<br><br>- The New York State Youth Alcohol Survey.<br><br>- Unclear about the significance of effects.

[49]	First author, year, country	General aim	Study design	The policy measure(s) or intervention(s) at hand	Measured policy effects on MLDA	Reflection on policy and other occurrences by authors	Comments
	Yu, 1998, USA.	This study aims to examine: (1) the change of alcohol use and purchase patterns among the underaged immediately after the raise of the purchase age; (2) the long-term change in underage alcohol use and purchase patterns after the raise of the purchase age; and (3) the change in impaired driving practices among youth over time after the raise of the purchase age.	- Five telephone surveys were conducted with youths aged 16 to 24 in 10 sampled New York State counties in 1982, 1983, 1985, 1986, and 1996.<br>- Data were collected through telephone interviews from randomly selected New Yorkers (a series of five surveys were conducted in 1982, before the enactment of the 19-drinking age law; 1983, after the enactment of the 19-drinking age law, 1985, before the 21-drinking law; 1986, after the 21-drinking age law, and in 1996, a decade after the 21-purchase age was enacted).<br>- Two dependent variables are included: respondents' frequency of drinking in the past month and frequency of driving under the influence of alcohol in the past month.<br>- A three-stage stratified proportionate random sampling procedure was designed. The 57 non-New York City counties were stratified on the dimension of a county's young adult population (16-20 in 82 and 83, 16-24 in 85 and 86) and a county's alcohol crash involvement among those young adults.	New York state raised the legal alcohol drinking age twice in the 1980's, from 18 to 19 in 1982 and from 19 to 21 in 1985.	- Analysis of the self-reported data showed that, 10 years after the enactment of the 21-drinking age law, alcohol use among 18-, 19-, and 20-year-olds decreased by up to 58% (sig.).<br>- Frequent heavy weekend drinking was reduced by 53% for 16-year-olds and 43% for 17-year-olds between 1982 and 1996 (sig.).<br>- Over the following 10 years, the prevalence of self-reported alcohol use increased slightly to 73%, representing only a 1% decline from the 1985 rate for respondents who were 21 and older.<br>- Alcohol purchase rates of 19- and 20-year-olds were reduced by -70% from 1985 to 1996 (sig.).<br>- From 1982 (before the 19 law) to 1996, the impaired driving rates for 18-year-olds decreased by 84%.<br>- Although impaired driving rates declined over the survey years for each age group, -25% of all underage respondents in 1996 reported that they had ridden in a vehicle with an impaired driver.<br>- Between 1982 and 1983, when the purchase age was raised to 19, the perceived parental approval of alcohol use for the 18-year-old respondents dropped from 69% to 42%. Between 1985 and 1986, when the purchase age was raised to 21, the perceived parental approval rates changed from 68% to 48% for 19-year-olds and from 78% to 56% for 20-year-olds.<br>- In 1996, 69%, 76%, 77%, 82% and 80%, respectively, of the 16-, 17-, 18-, 19-, and 20-year-old respondents thought their peers would approve of their drinking.	- Both the 19 and 21 purchase age laws had immediate impact on alcohol purchase and use by the targeted youth groups. A decade later, the effectiveness of the 21-purchase age law continues.<br>- Analyses of 21-to 24-year-old respondents, who were not affected by the change in the law, showed that alcohol purchase and use rates did not significantly decline from 1985 to 1996. This finding provides further evidence that the purchase age laws tend to have a distinctive impact on the age groups which the laws specifically target.<br>- Weekend drinking tends to be one of the most important factors in youth highway crashes. Findings indicate that weekend drinking and heavy weekend drinking decreased, however, many underage respondents indicated drinking alcohol away from their own home, most commonly at friends' houses. After the establishment of the higher alcohol purchase ages, when youths cannot use alcohol at home in the presence of their parents, their alternative drinking location would be their friends' houses where parents may be absent.<br>- Continued enforcement of the 21-purchase age law will maximize its effect in reducing underage purchase and use of alcohol. Enforcement efforts may emphasize parental involvement in media campaigns.<br>- Anti-drunk driving campaigns, such as "friends don't let friends drive drunk," should not only target adult party-bar goers, but also target underage youths. "Zero Tolerance" laws, which reduce the illegal blood alcohol concentration level to 0.02 for drivers under the age of 21 and which have been enacted in 45 states and the District of Columbia, will also provide additional leverage to enforce the 21-purchase age law.	**INCLUDE.**<br><br>- The New York State Youth Alcohol Survey.<br>- New York State Department of Motor Vehicles records.<br><br>- Follow-up on study [48], using comparable data and methods, only now measuring long-time changes.<br><br>- Unclear about the significance of effects.

[50]	**First author, year, country**	**General aim**	**Study design**	**The policy measure(s) or intervention(s) at hand**	**Measured policy effects on MLDA**	**Reflection on policy and other occurrences by authors**	**Comments**
	Sannen, 2014, the Netherlands.	The aim of this exploration is to gain more insight into possible shifts in alcohol- and/or drug use among 16- and 17-year-olds after the change in the minimum legal drinking age.	- In order to obtain a quick scope of the situation, prevention workers of thirteen regular institutions for addiction-care in the Netherlands were consulted.<br>- In the summer of 2014, a number of questions were submitted to 23 contacts at these institutions.<br>- At that time, the change in the minimum legal drinking age was in force for more than six months.	The minimum legal drinking age in the Netherlands was changed on January 2014 from 16 to 18 years. Vendors are no longer allowed to sell alcohol to individuals younger than 18 years old, and individuals under 18 are punishable by law if they carry alcohol in publicly accessible places.	- Prevention workers generally do not see signs of increased illicit drug use among 16 and 17-year-olds after the change in legislation.<br>- If they see changes in illicit drug use, these changes are not always associated with the increase in the alcohol age from 16 to 18 years.<br>- The signals that may indicate a shift in alcohol or illicit drug use among 16 and 17-year-olds after the change in legislation are usually concerning very specific groups of young people, limited in size.<br>- Prevention workers do not expect a massive shift in the future either. Reasons for this are that young people still are required an effort to obtain illicit drugs. Also, alcohol is still easily available for underage individuals in the Netherlands, and, for most young people, changing from alcohol to illicit drugs is a big change or transmission.	- Respondents only observe few desirable effects of the increase of the minimum legal drinking age in the Netherlands. They see that 16- and 17-year-olds continue to drink because it is still easy to obtain alcohol when going out, for example obtaining drinks from older friends.<br>- According to the prevention workers, a significant proportion of 16- and 17-year-olds engage in drinking more out of sight, on private property. Parents allow and facilitate this by providing accommodations and alcohol, because they do not support the change in legislation and because they are afraid that their children will go out and drink on the street.	**INCLUDE.**<br><br>-

[51]	First author, year, country	General aim	Study design	The policy measure(s) or intervention(s) at hand	Measured policy effects on MLDA	Reflection on policy and other occurrences by authors	Comments
	Hingson, 1985, USA.	This study explores whether, when Massachusetts raised its legal drinking age from 18 to 20 in early 1979, significant declines occurred in that state in 1) teenage drinking and in turn, 2) homicide rates, 3) suicide rates, and 4) deaths from nontraffic accidents in 1980, 1981 and 1982 relative to New York State, where the legal drinking age remained at 18 during that time.	- An anonymous random digit dialing telephone survey of 16 to 19-year-olds was conducted in Massachusetts and New York (control), prior to enactment of the law in 1979, asking teenagers about their personal characteristics, drinking practices (average drinks daily), procurement of alcohol, use of psychoactive drugs and possible experiences with police enforcement when obtaining alcohol. Twice and yearly intervals following the law were conducted (six waves for each state). Log linear analyses was used to test the impact of the law on dependent variables.<br>- Interviews were conducted with 50 Massachusetts police officers and inspectors in all levels of command and context, asking them about enforcement practices before and after the drinking age change.<br>- Arrest data from the Uniform Crime Reporting system were evaluated before and after the law change for both states.<br>- The number of fatalities from non-traffic accidents, suicides, and homicides were analyzed separately, collating data into pre- and post-law groups. The number of fatalities was fit to a log linear model.	Raise of the legal drinking age from 18 to 20 in early 1979 in Massachusetts.	- After the law, the frequency of teenage drinking in bars-clubs-restaurants and the percentage of teenagers reporting they most often obtained their alcohol in liquor stores and groceries dropped in Massachusetts compared with New York (sig.).<br>- One third attempting to purchase liquor indicated they were never asked for ID, 5% were stopped by the police just once, none were arrested the first year after the law.<br>- Two years after the law, the proportion of Massachusetts teenagers who had someone else purchase alcohol for them nearly doubled (sig.).<br>- The average daily consumption of alcohol in the target population (15 to 19-year-olds) two years after the law did not significantly decline in Massachusetts compared to New York.<br>- After the law change, arrests among the target population rose over 150% (for multiple alcohol-related offenses).<br>- The intensity of enforcement was varied between communities caused by lack of personnel and competing priorities (as the main reasons). Also, many officers did not perceive underage drinking or purchasing alcohol as a serious crime.<br>- During the three years after Massachusetts raised its drinking age, compared with New York, there were no significant changes among the target population in the number of deaths from: 1) accidental injury other than traffic accidents, 2) the number of suicides or 3) homicide deaths.	- This study found no reductions in non-motor vehicle accident deaths, homicides, or suicides relative to New York among 15 to 19-year-olds after Massachusetts raised its drinking age from 18 to 20.<br>- It is possible that some types of nontraffic accidents, homicides, and suicide are also more likely than others to involve alcohol, but the possible relations are not well established and hence the specific subcategories of violent death are not monitored over time.<br>- Despite similarities between states, it is possible that one of the two experienced a change that could confound a change in violent death rates.<br>- The actual contribution that drinking makes is quite small relative to other independent predictors of other causes of death.<br>- It is conceivable that changes in location of drinking (e.g., form bars to drinking at home) might reduce the frequency of nighttime travel and rates of fatal crashes.	INCLUDE.<br><br>- An anonymous random digit dialing telephone survey of 16 to 19-year-olds.<br>- Interviews with 50 Massachusetts police officers and inspectors.<br>- Arrest data from the Uniform Crime Reporting system.<br>- The number of fatalities from non-traffic accidents, suicides, and homicides.

Second path: Primary societal impact

[44]	First author, year, country	General aim	Study design	The policy measure(s) or intervention(s) at hand	Measured policy effects on MLDA	Reflection on policy and other occurrences by authors	Comments
	Hingson, 1983, USA.	- This paper examines the impact of raising the drinking age on teenage drinking, driving after drinking, and non-fatal accident involvement in Massachusetts prior to the law's enactment and twice at yearly intervals after the law enactment.<br>- Massachusetts was compared with New York State, who retained an 18-year-old drinking age.	- Random telephone surveys with approximately 1,000 16-19-year-olds in each state were undertaken prior to the law's enactment and twice at yearly intervals after the law, to assess the law's impact on teenage drinking, driving after drinking, and non-fatal accident involvement.<br>- Respondents were asked where they most often obtained their alcohol (e.g., liquor/grocery store, bar/club, at home) and where they drank > 5 consumptions in the last month (e.g., party, bar).<br>- Drinking was measured asking for 'any drinking' in the last month and 'drinking 6+ drinks at one time' in the last month.<br>- Log-linear analysis was used on the survey data.<br>- Fatal crash data reported to the US Department of Transportation by each state from 1976-1981 were also analyzed.<br>- To assess law enforcement practices and problems, interviews were conducted with over 50 Massachusetts police officers representing all levels of command in urban, rural, and suburban jurisdictions.	- Legislation raising the legal drinking age in Massachusetts from 18 to 20 in 1979.<br>- At the time Massachusetts raised its legal drinking age from 18 to 20, the two states had similar laws regarding age of driving licensure and penalties for driving while intoxicated.	- After the law, the frequency of teenage drinking in bars and clubs and the percentage of teenagers reporting they most often obtained their alcohol in liquor stores and groceries dropped in Massachusetts compared to New York. (sig.).<br>- During the two years after the law, the proportion of Massachusetts teenagers who had someone else purchase alcohol for them or who most often obtained alcohol from their homes nearly doubled (sig.).<br>- The average daily consumption of alcohol in the 16-19-year-old age group during the two years after the law did not decline in Massachusetts compared to New York. Nor did teenagers report shifts to the use of other psychoactive drugs.<br>- After the law, the proportion of teenagers who reported driving after drinking heavily (six or more drinks) did not decline in either state. However, the frequency that teenagers reported they drove after any drinking declined significantly more in Massachusetts.<br>- The three methods of statistical analysis indicated no significant difference between the two states in the overall teenage fatal accident trends (not sig.).<br>- The frequency with which teenagers were arrested for driving under the influence did not significantly change in Massachusetts during the first year after the law compared to the previous two years.<br>- Enforcement of the law focusing on the sellers was minimal and sporadic.<br>- The intensity of enforcement varied widely from community to community.<br>- The reasons most often cited for the variability in enforcement of the law among communities across the state was the lack of personnel and competing priorities, particularly in some high crime inner-city jurisdictions. Moreover, many officers did not perceive teenage purchasing of alcohol or drinking per se as a sufficiently serious crime to stigmatize Juveniles by putting an arrest on their records.	- The study examined the first two years following enactment of the law. During this time period, the 18 and 19-year-old age groups who had previously been allowed to drink had that privilege revoked. One could hypothesize that the previous drinking habits of this group would be resistant to change.<br>- The state's law provides a symbolic statement to teenagers that its citizens disapprove of their drinking, and fears the accidents they may cause when they drive after drinking. The study results prompt us to ask whether the law could have had a greater impact among all Massachusetts teenagers if enforcement efforts were more consistent and if greater attention were paid to preventing the common practices of non-teenagers purchasing alcohol for teenagers or liquor outlets not requiring age identification. Without sufficient resources and coordination of enforcement efforts, those police who actively strive to enforce the law in one community may find their efforts negated by minimal enforcement in the next.<br>- Lack of community resources and variable willingness to enforce laws focused on teenagers raise questions about whether alternative strategies such as increased enforcement of the drunk driving and traffic safety laws aimed at all drivers, or requirements for safer cars and improved road design would yield greater reductions in nonfatal and fatal accidents both among teenagers and non-teenagers.	**INCLUDE.**<br><br>- In addition to telephone surveys, data from the Fatality Analysis Reporting System (FARS) was used.

[45]	First author, year, country	General aim	Study design	The policy measure(s) or intervention(s) at hand	Measured policy effects on MLDA	Reflection on policy and other occurrences by authors	Comments
	Vingilis, 1981, Canada.	This paper reports four types of data which are relevant to the study of the effects of the increase in the MLDA.	Data are from:<br>1) surveys of drinking and drinking problems among high school students (measuring: 1) alcohol use in the month prior, 2) feeling 'tight' at least once in the month prior, 3) 'drunk' at least once in the month prior, 4) 5 or more drinks on a singes occasion at least once in the month prior). Conducted in 1977 and 1979, stratified proportional sample in the province. Log linear analyses.<br>2) a study (surveys) of perceptions of vice-principals. Conducted in 1972 and 1980.<br>3) a trend analyses of young drinking offender charges; and<br>4) trend analyses of drinking-driving statistics and driver fatalities. Two types of drinking-driving statistics were obtained: monthly Ontario drinking-driving convictions form 1977 and 1979 for 16-21-year-olds and monthly Ontario accident fatalities from 1973 to 1979 for 16-21-year-olds. Time series analyses were used.	The increase of the MLDA in 1979 in Ontario (from 18 to 19).	1) the proportion of drinkers among 18-19-year-olds decreased from 1977 to 1979 (as well as a decrease in the proportion feeling 'tight'). Comparable effects were observed for younger drinkers (under 18) in the exact opposite direction.<br>2) The findings from this study indicate that the vice-principals have perceived either no change or less student drinking and alcohol-related problems at their school since the increase in legal drinking age (between 20 per cent and 30 per cent of the vice-principals reported a decrease in drink-related behaviors).<br>4) There were no significant differences between the pre-intervention and post-intervention time periods for both types of drinking-driving statistics.	- The results of the four studies indicate few statistically significant changes, however, these findings seem to tentatively suggest a minimal effect for 18-19-year-old high school students, but not for the regular (once a week or more) or younger drinkers.<br>- The fact that regular drinkers reported no changes in their drinking habits could partially explain why major changes were not observed for the more general methods of measurement, that is, the rates of accidents, charges and convictions and the perceptions of vice-principals.<br>- Additionally, a large proportion of Ontario youths are not in school and these non-students may be heavier drinkers. Conviction and fatality statistics are not sensitive enough measures, as it seems that too few high-accident-and-arrest-risk youths were changing their drinking patterns for an effect to emerge from these statistics.<br>- The minimal Ontario effect was expected, as it was felt that the one-year increase was not sufficient to cause a major impact on youthful drinking behavior.	INCLUDE.<br><br>- Data from the Fatal Accident Reporting System (FARS).<br><br>- Only 1, 2 and 4 approved criteria and are extracted.

[49]	First author, year, country	General aim	Study design	The policy measure(s) or intervention(s) at hand	Measured policy effects on MLDA	Reflection on policy and other occurrences by authors	Comments
	Yu, 1998, USA.	This study aims to examine: (1) the change of alcohol use and purchase patterns among the underaged immediately after the raise of the purchase age; (2) the long-term change in underage alcohol use and purchase patterns after the raise of the purchase age; and (3) the change in impaired driving practices among youth over time after the raise of the purchase age.	- Five telephone surveys were conducted with youths aged 16 to 24 in 10 sampled New York State counties in 1982, 1983, 1985, 1986, and 1996.<br>- Data were collected through telephone interviews from randomly selected New Yorkers (a series of five surveys were conducted in 1982, before the enactment of the 19-drinking age law; 1983, after the enactment of the 19-drinking age law, 1985, before the 21-drinking law; 1986, after the 21-drinking age law, and in 1996, a decade after the 21-purchase age was enacted).<br>- Two dependent variables are included: respondents' frequency of drinking in the past month and frequency of driving under the influence of alcohol in the past month.<br>- A three-stage stratified proportionate random sampling procedure was designed. The 57 non-New York City counties were stratified on the dimension of a county's young adult population (16-20 in 82 and 83, 16-24 in 85 and 86) and a county's alcohol crash involvement among those young adults.	New York state raised the legal alcohol drinking age twice in the 1980's, from 18 to 19 in 1982 and from 19 to 21 in 1985.	- Analysis of the self-reported data showed that, 10 years after the enactment of the 21-drinking age law, alcohol use among 18-, 19-, and 20-year-olds decreased by up to 58% (sig.).<br>- Frequent heavy weekend drinking was reduced by 53% for 16-year-olds and 43% for 17-year-olds between 1982 and 1996 (sig.).<br>- Over the following 10 years, the prevalence of self-reported alcohol use increased slightly to 73%, representing only a 1% decline from the 1985 rate for respondents who were 21 and older.<br>- Alcohol purchase rates of 19- and 20-year-olds were reduced by -70% from 1985 to 1996 (sig.).<br>- From 1982 (before the 19 law) to 1996, the impaired driving rates for 18-year-olds decreased by 84%.<br>- Although impaired driving rates declined over the survey years for each age group, -25% of all underage respondents in 1996 reported that they had ridden in a vehicle with an impaired driver.<br>- Between 1982 and 1983, when the purchase age was raised to 19, the perceived parental approval of alcohol use for the 18-year-old respondents dropped from 69% to 42%. Between 1985 and 1986, when the purchase age was raised to 21, the perceived parental approval rates changed from 68% to 48% for 19-year-olds and from 78% to 56% for 20-year-olds.<br>- In 1996, 69%, 76%, 77%, 82% and 80%, respectively, of the 16-, 17-, 18-, 19-, and 20-year-old respondents thought their peers would approve of their drinking.	- Both the 19 and 21 purchase age laws had immediate impact on alcohol purchase and use by the targeted youth groups. A decade later, the effectiveness of the 21-purchase age law continues.<br>- Analyses of 21-to 24-year-old respondents, who were not affected by the change in the law, showed that alcohol purchase and use rates did not significantly decline from 1985 to 1996. This finding provides further evidence that the purchase age laws tend to have a distinctive impact on the age groups which the laws specifically target.<br>- Weekend drinking tends to be one of the most important factors in youth highway crashes. Findings indicate that weekend drinking and heavy weekend drinking decreased, however, many underage respondents indicated drinking alcohol away from their own home, most commonly at friends' houses. After the establishment of the higher alcohol purchase ages, when youths cannot use alcohol at home in the presence of their parents, their alternative drinking location would be their friends' houses where parents may be absent.<br>- Continued enforcement of the 21-purchase age law will maximize its effect in reducing underage purchase and use of alcohol. Enforcement efforts may emphasize parental involvement in media campaigns.<br>- Anti-drunk driving campaigns, such as "friends don't let friends drive drunk," should not only target adult party-bar goers, but also target underage youths. "Zero Tolerance" laws, which reduce the illegal blood alcohol concentration level to 0.02 for drivers under the age of 21 and which have been enacted in 45 states and the District of Columbia, will also provide additional leverage to enforce the 21-purchase age law.	**INCLUDE.**<br><br>- The New York State Youth Alcohol Survey.<br>- New York State Department of Motor Vehicles records.<br><br>- Follow-up on study [48], using comparable data and methods, only now measuring long-time changes.<br><br>- Unclear about the significance of effects.

[50]	First author, year, country	General aim	Study design	The policy measure(s) or intervention(s) at hand	Measured policy effects on MLDA	Reflection on policy and other occurrences by authors	Comments
	Sannen, 2014, the Netherlands.	The aim of this exploration is to gain more insight into possible shifts in alcohol- and/or drug use among 16- and 17-year-olds after the change in the minimum legal drinking age.	- In order to obtain a quick scope of the situation, prevention workers of thirteen regular institutions for addiction-care in the Netherlands were consulted.<br>- In the summer of 2014, a number of questions were submitted to 23 contacts at these institutions.<br>- At that time, the change in the minimum legal drinking age was in force for more than six months.	The minimum legal drinking age in the Netherlands was changed on January 2014 from 16 to 18 years. Vendors are no longer allowed to sell alcohol to individuals younger than 18 years old, and individuals under 18 are punishable by law if they carry alcohol in publicly accessible places.	- Prevention workers generally do not see signs of increased illicit drug use among 16 and 17-year-olds after the change in legislation.<br>- If they see changes in illicit drug use, these changes are not always associated with the increase in the alcohol age from 16 to 18 years.<br>- The signals that may indicate a shift in alcohol or illicit drug use among 16 and 17-year-olds after the change in legislation are usually concerning very specific groups of young people, limited in size.<br>- Prevention workers do not expect a massive shift in the future either. Reasons for this are that young people still are required an effort to obtain illicit drugs. Also, alcohol is still easily available for underage individuals in the Netherlands, and, for most young people, changing from alcohol to illicit drugs is a big change or transmission.	- Respondents only observe few desirable effects of the increase of the minimum legal drinking age in the Netherlands. They see that 16- and 17-year-olds continue to drink because it is still easy to obtain alcohol when going out, for example obtaining drinks from older friends.<br>- According to the prevention workers, a significant proportion of 16- and 17-year-olds engage in drinking more out of sight, on private property. Parents allow and facilitate this by providing accommodations and alcohol, because they do not support the change in legislation and because they are afraid that their children will go out and drink on the street.	**INCLUDE.**<br><br>-

[51]	First author, year, country	General aim	Study design	The policy measure(s) or intervention(s) at hand	Measured policy effects on MLDA	Reflection on policy and other occurrences by authors	Comments
	Hingson, 1985, USA.	This study explores whether, when Massachusetts raised its legal drinking age from 18 to 20 in early 1979, significant declines occurred in that state in 1) teenage drinking and in turn, 2) homicide rates, 3) suicide rates, and 4) deaths from nontraffic accidents in 1980, 1981 and 1982 relative to New York State, where the legal drinking age remained at 18 during that time.	- An anonymous random digit dialing telephone survey of 16 to 19-year-olds was conducted in Massachusetts and New York (control), prior to enactment of the law in 1979, asking teenagers about their personal characteristics, drinking practices (average drinks daily), procurement of alcohol, use of psychoactive drugs and possible experiences with police enforcement when obtaining alcohol. Twice and yearly intervals following the law were conducted (six waves for each state). Log linear analyses was used to test the impact of the law on dependent variables.<br>- Interviews were conducted with 50 Massachusetts police officers and inspectors in all levels of command and context, asking them about enforcement practices before and after the drinking age change.<br>- Arrest data from the Uniform Crime Reporting system were evaluated before and after the law change for both states.<br>- The number of fatalities from non-traffic accidents, suicides, and homicides were analyzed separately, collating data into pre- and post-law groups. The number of fatalities was fit to a log linear model.	Raise of the legal drinking age from 18 to 20 in early 1979 in Massachusetts.	- After the law, the frequency of teenage drinking in bars-clubs-restaurants and the percentage of teenagers reporting they most often obtained their alcohol in liquor stores and groceries dropped in Massachusetts compared with New York (sig.).<br>- One third attempting to purchase liquor indicated they were never asked for ID, 5% were stopped by the police just once, none were arrested the first year after the law.<br>- Two years after the law, the proportion of Massachusetts teenagers who had someone else purchase alcohol for them nearly doubled (sig.).<br>- The average daily consumption of alcohol in the target population (15 to 19-year-olds) two years after the law did not significantly decline in Massachusetts compared to New York.<br>- After the law change, arrests among the target population rose over 150% (for multiple alcohol-related offenses).<br>- The intensity of enforcement was varied between communities caused by lack of personnel and competing priorities (as the main reasons). Also, many officers did not perceive underage drinking or purchasing alcohol as a serious crime.<br>- During the three years after Massachusetts raised its drinking age, compared with New York, there were no significant changes among the target population in the number of deaths from: 1) accidental injury other than traffic accidents, 2) the number of suicides or 3) homicide deaths.	- This study found no reductions in non-motor vehicle accident deaths, homicides, or suicides relative to New York among 15 to 19-year-olds after Massachusetts raised its drinking age from 18 to 20.<br>- It is possible that some types of nontraffic accidents, homicides, and suicide are also more likely than others to involve alcohol, but the possible relations are not well established and hence the specific subcategories of violent death are not monitored over time.<br>- Despite similarities between states, it is possible that one of the two experienced a change that could confound a change in violent death rates.<br>- The actual contribution that drinking makes is quite small relative to other independent predictors of other causes of death.<br>- It is conceivable that changes in location of drinking (e.g., form bars to drinking at home) might reduce the frequency of nighttime travel and rates of fatal crashes.	INCLUDE.<br><br>- An anonymous random digit dialing telephone survey of 16 to 19-year-olds.<br>- Interviews with 50 Massachusetts police officers and inspectors.<br>- Arrest data from the Uniform Crime Reporting system.<br>- The number of fatalities from non-traffic accidents, suicides, and homicides.

[52]	First author, year, country	General aim	Study design	The policy measure(s) or intervention(s) at hand	Measured policy effects on MLDA	Reflection on policy and other occurrences by authors	Comments
	Grucza, 2009, USA.	To evaluate trends in the past 30-day prevalence of binge drinking by age, gender, and student-status among youth and young adults in the United States between 1979 and 2006.	- Data were analysed from twenty administrations of the National Survey on Drug Use and Health, calculating trends in relative risk for four different age groups, student status and race/ethnicity, stratified by gender and relative to the 24-34-year-old reference group.<br>- Binge drinking has been queried as the number of days in the past 30 days in which an individual has consumed five or more drinks on any one occasion. In the 1979 survey, individuals were asked about the largest number of drinks they had in any 1 day in the past 30 days. These questions were used to determine the prevalence of individuals who had drunk five drinks in a day, at least once in the past 30 days.<br>- Multistage probability sampling was used for all surveys.	The federally mandated transition to a uniform legal drinking age of 21 years, and other policy changes aimed at curbing underage drinking.	- Individuals younger than 20 years have experienced marked reductions in risk for binge drinking, suggesting that changes in the MLDA, as well as other policy changes and public health campaigns, have been successful.<br>- Countering the former trend, risk for binge drinking among girls and young women has been rising, with risk increasing faster for minorities than for whites.<br>- The reduction in risk for binge drinking among youths has not reached college students.	The reduction in binge drinking among the youths in general is likely to be at least partly attributable to the adoption of the uniform drinking age of 21 years. Other policy changes (e.g., zero-tolerance driving under the influence laws) may also have had effects but have not been as thoroughly studied as the MLDA and zero-tolerance policies.	**INCLUDE.**<br><br>- Data is used from the National Survey on Drug Use and Health (NSDUH) and the formerly known National Household Survey on Drug Abuse (NHSDA).

[53]	First author, year, country	General aim	Study design	The policy measure(s) or intervention(s) at hand	Measured policy effects on MLDA	Reflection on policy and other occurrences by authors	Comments
	Jager, 2015, USA.	- Drawing from a developmental-contextual perspective, and using multi-cohort panel data, we examine how the trajectories of age 18–26 binge drinking (i.e., consuming five or more drinks in a row), have changed across the last three decades.<br>- We focus on two components of the chronosystem that may help explain historical variation in young adult binge drinking trajectories: (a) historical variation in the frequency and timing of social role acquisition and (b) historical variation in minimum legal drinking age (MLDA).	- As part of the national Monitoring the Future Study, over 64,000 youths from 28 consecutive cohorts of high-school seniors between 1976 and 2004 were surveyed at biennial intervals between ages 18 and 26.<br>- We focused on the first 5 waves of the MTF panel data, using Full Information Maximum Likelihood estimation analyses.<br>- The output measure of this study is 'binge-drinking', operationalized as 5 or more drinks in a row in the past two weeks.	- Historical increases in minimum legal drinking age.<br>- The 'Big 5' young adult social roles (marriage, parenthood, education, residential status, and employment) were also outcome of interest but not a measure or intervention.	- For both genders, the positive quadratic effect of cohort year on age 18 binge drinking was no longer significant once MLDA laws were controlled, thus linking MLDA laws that were implemented in the mid-1980s with the sharp decline in age 18 binge drinking across the mid-1980s and early 1990s (sig.).<br>- We found that historical increases in minimum legal drinking age account for a portion of the historical decline in age 18 levels of binge drinking, while historical variation in social role acquisition (e.g., marriage, parenthood, and employment) accounts for a portion of the historical acceleration in age 18–22 growth.	Future research should focus on other proximal contextual factors that are known to be associated with binge drinking and have potentially varied historically (in both linear and non-linear fashions). Although by no means an exhaustive list, the following are known to be associated with binge drinking: the perceived risk and availability of alcohol, parental attitudes as well as peer norms regarding alcohol use, and alcohol abuse prevention efforts, including school- based programs, media campaigns.	**INCLUDE.**<br><br>- Data from the 1976–2004 Monitoring the Future (MTF) surveys were used.

[54]	**First author, year, country**	**General aim**	**Study design**	**The policy measure(s) or intervention(s) at hand**	**Measured policy effects on MLDA**	**Reflection on policy and other occurrences by authors**	**Comments**
	Johnson, 1992, Canada.	The purpose of this paper is to use aggregate time series compiled from data for each province of Canada to estimate the short-run and long-run responsiveness of consumption to price, income, and the legal drinking age	Elasticities for beer, wine and spirits are estimated for each of the provinces of Canada over the period 1956-1983, using unrestricted dynamic regressions modelled after the error-correction mechanism. Alternative long-run estimates are also obtained from cointegrating regressions.	Alcohol consumption to price, income and the legal drinking age.	- Estimates vary markedly across provinces and suggest that increases in price will reduce consumption of all beverages in the short run, but in the long run no evidence is found that spirits use is price-sensitive.<br>- Increases in the legal drinking age reduce consumption in the short run (an increase of one year in the legal drinking age will reduce the demand for beer by about 2% to 3% with typically a larger effect on the demand for wine and no evidence for spirits) but there is little indication of a long-run effect.	-	**INCLUDE.**<br><br>- A detailed appendix explaining the data construction and a listing of the data are available on request.

[55]	First author, year, country	General aim	Study design	The policy measure(s) or intervention(s) at hand	Measured policy effects on MLDA	Reflection on policy and other occurrences by authors	Comments
	Krauss, 2015, USA.	We examined whether exposure to permissive MLDA laws during adolescence has long-term effects on illicit drug use and disorders in adulthood.	- Participants from the NSDUH were linked with historical state MLDA laws. Participants born in 1949–1972 (age 31–63 years at observation were analyzed because they came of legal age for alcohol purchase when changes occurred in state MLDA laws.<br>- The main outcomes were use of marijuana and use of other illicit drugs. We used variables provided by the NSDUH which flagged whether the participant had used marijuana in the past month, used marijuana in the past year, and met the criteria for Diagnostic and Statistical Manual of Mental Disorders 4th edition (DSM-IV) marijuana abuse or dependence. Similarly, we used recoded variables that flagged whether the participant had used illicit drugs other than marijuana in the past month, used illicit drugs other than marijuana in the past year, and met the criteria for abuse or dependence for illicit drugs other than marijuana. Illicit drugs other than marijuana included cocaine, heroin, hallucinogens, inhalants, and the non-medical use of pain relievers, sedatives, stimulants, or tranquilizers.<br>- Logistic regression was used to model drug use measures as a function of exposure to permissive MLDA during adolescence, adjusting for state and birth-year fixed effects, demographics, and salient state characteristics.	The time period in which between-state and cross-year differences in minimum legal drinking age policies were present (participants were 18–20 years old in 1967–1992).	- Exposure to permissive MLDA laws was not significantly associated with drug use or abuse/dependence in adulthood.<br>- Men exposed to permissive MLDA laws were at 20% increased odds of past year illicit drug use.	- The move toward lower legal drinking ages occurred during a period when age-of-majority laws were being liberalized, such as age of birth control access. Thus, MLDA associations may be confounded with these other policy changes, which may also have influenced drinking and drug use behaviors. However, the increases in MLDA that occurred between 1977 and 1988 were in response to public health concerns and occurred after the move toward lower age-of-majority laws. Stratifying our sample into two cohorts (one in which they primarily experienced decreases and one in which they experienced increases) showed that, in general, effects did not differ substantially between these two cohorts. Thus, MLDA associations do not appear to be confounded by other changes in age-of-majority policies.	**INCLUDE.**<br><br>- Data from the National Survey of Drug Use and Health (NSDUH) were used.

[56]	**First author, year, country**	**General aim**	**Study design**	**The policy measure(s) or intervention(s) at hand**	**Measured policy effects on MLDA**	**Reflection on policy and other occurrences by authors**	**Comments**
	Laixuthai, 1993, USA.	This paper examines the frequency of youth drinking and heavy drinking in 1982 and 1989 to 1) investigate the effects of MLDA and alcoholic beverage prices on youth drinking, and 2) investigate the effects of a uniform age of 21 on the price sensitivity of youth alcohol use.	- Dichotomous probit methods are used for analysis.<br>- Categorical information on the number of drinking occasions in the last 30 days, the last year, and in the respondent's lifetime, as well as the number of times in the last two weeks that he or she had five or more drinks in one occasion is obtained. This information was rearranged in comparable variables for analysis.	- The MLDA and federal excise taxes on beer (in 1982 and 1989).	- Increased alcoholic beverage prices and/or minimum legal drinking ages reduce the frequency of alcohol consumption and heavy drinking by youths (sig.).<br>- Reductions in drinking are not limited to infrequent drinkers only, even larger reductions occur in the numbers of youths who drink frequently or fairly frequently.<br>- The price sensitivity of youth alcohol use fell after the change to a uniform legal drinking age of 21.<br>- Underage youth who live within 25 miles of a state with a lower MLDA apparently cross the border to obtain alcoholic beverages.	The full price of consuming alcohol for a youth is the sum of both the money price and the indirect costs (the legal obstacles to consumption, such as a higher MLDA). Other indirect costs include the money and time to obtain a fake ID, to obtain the alcohol itself and others. Increased MLDA and taxes both increase the full price of drinking for underage youth. In 1989, high school seniors face a greater indirect cost of obtaining alcohol than they did in 1982 due to the uniform MLDA of 21. Therefore, the same increase in tax has a smaller impact on full price in 1989 (and consequently, consumption), than in 1982.	**INCLUDE.**<br><br>- Data from the 1982 and 1989 Monitoring the Future (MTF) surveys.<br>- Data from the American Chamber of Commerce Researchers Association is used for the tax variable.

[57]	First author, year, country	General aim	Study design	The policy measure(s) or intervention(s) at hand	Measured policy effects on MLDA	Reflection on policy and other occurrences by authors	Comments
	Miron, 2009, USA.	We challenge the view that MLDAs reduce traffic fatalities based on three findings. First, the overall impact estimated in earlier research is driven by states that increased their MLDA prior to any inducement from the federal government. Second, even in early-adopting states, the impact of the MLDA did not persist much past the year of adoption. Third, the MLDA has at most a minor impact on teen drinking.	- We examine the relation between MLDAs and traffic fatalities using aggregate road facilities data of the complete population and of 15-24-year-olds and state-level panel data, reconstructing the analysis of Dee (1999) (using FARS to construct a panel data set for the 48 contiguous states over the period 1977–1992) and extending it to include Alaska, Hawaii, and Washington DC, and the years 1976 and 1993–2005. We focus on 18- to 20-yr-old fatalities.<br>- Using MTF data, we employ the two specific measures common in the literature, ‘‘drinker’’ (having any drink of alcohol in the last month) and ‘‘heavy episodic drinker’’ (having five or more drinks in a row at some point in the last 2 weeks). We also examine the number of motor vehicle accidents that respondents report as occurring after consuming alcohol. We estimate regressions similar to the traffic fatality calculations with these dependent variables. The measure of the MLDA is identical to that used in previous literature, a dummy for having a drinking age of 18 years.	- Changes in de MLDA over the period 1976-2005.<br>- Mandatory seatbelt law.<br>- The BAC limit for legal driving.<br>- Beer taxes.<br><br>We omit several potentially relevant policies, in part because of data availability, in part to conform with Dee (1999), and in part because previous studies have found limited evidence of any impact on TFRs. These variables include dram shop liability laws, mandatory sentences for driving under the influence, sobriety check points, anti-plea-bargaining statutes, changes in tort liability laws that place greater responsibility with intoxicated drivers, happy hour regulations, enforcement (however, enforcement is too low to have any impact on the results examined) and alcohol education programs.	- The MLDA fails to have the fatality-reducing effects that previous articles have reported; trends between 1910 and 2000 in aggregate data of road facility rates show no difference in trends between 15-24-year-olds and that of the entire population.<br>- The declines in the total and 15–24 TFR that began around 1969 long precede the adoptions of an MLDA of 21 in the mid-1980s. The key fact about TFRs, therefore, is that they have been trending downward for decades and have been poorly correlated with the MLDAs.<br>- State-level panel data for the past 30 years show that any nationwide impact of the MLDA is driven by states that increased their MLDA prior to any inducement from the federal government (FUDAA). Even in early-adopting states, the impact of the MLDA did not persist much past the year of adoption.<br>- The MLDA appears to have only a minor impact on teen drinking (sig.), these reductions derive mainly from states that adopted the MLDA21 before enactment of the FUDAA.<br>- Nevertheless, when the number of accidents post-alcohol consumption was analyzed, the panel estimates reveal that movement away from an MLDA of 18 is associated with a statistically insignificant change in reporting of alcohol-related traffic accidents.	- These results suggest that, at most, the MLDA21 reduced TFR18–20 in states that adopted the policy on their own. This raises the question of endogeneity. The MLDA21 in these states may have been enacted in response to grassroots concern against drunk driving or implemented alongside other efforts to reduce traffic fatalities. Relatedly, states that adopted on their own may have been states that devoted significant resources to enforcement.<br>- The limited effects found in non-early adopting additionally challenges the desirability of coercive federalism.<br>- Landmark improvements in the accident avoidance and crash protection features of passenger cars (in place around 1970), and control for advances in medical technology might explain the drastic reductions in traffic fatalities over the past half century and should be made to additionally measure and control for as they are likely to be major determinants of the declining traffic fatality trends.	**INCLUDE.**<br><br>- Data from the Fatal Accident Reporting System (FARS) were used.<br>- Data from Monitoring the Future (MTF) surveys were used.

[58]	First author, year, country	General aim	Study design	The policy measure(s) or intervention(s) at hand	Measured policy effects on MLDA	Reflection on policy and other occurrences by authors	Comments
	Nelson, 2003, USA.	This study analyses the importance of several restrictive alcohol regulations on alcohol consumption. In contrast to previous research, the study allows for substitution among beverages as a response to a regulation that targets a specific beverage (a restrictive law that applies to only one beverage (or one form of advertising) can result in substitution toward other beverages (or other forms of advertising)).	- A panel of 45 states is used for the period 1982–1997, resulting in a longitudinal sample of 720 observations.<br>- Alcohol consumption per capita is measured in equivalent units of pure alcohol/ethanol, alcohol prices are obtained from quarterly surveys. Altered fixed-effects (FE) econometric models are used for analysis.	Examine patterns of substitution due to state/beverage laws for:<br>(1) Monopoly control of retail sale of alcohol (distribution laws);<br>(2) advertising bans for billboards and beverage prices (information laws);<br>(3) minimum legal drinking ages by beverage (usage laws).	- The empirical results demonstrate that monopoly control of spirits reduces consumption of that beverage, and increases consumption of wine.<br>- The effect on beer is positive, but is not statistically significant.<br>- The net effect of monopoly control on total alcohol is significantly negative.<br>- Higher minimum legal drinking age laws have negative effects on beverage and total alcohol consumption (sig.), in other words, for the regulatory variables, the MLDA results for 1982-88 indicate that higher legal drinking ages are an effective way to reduce alcohol consumption by youth.<br>- Bans of advertising do not reduce total alcohol consumption, which partly reflects substitution effects.	- This study finds that billboard bans may have unintended consequences on alcohol consumption. The empirical results indicate that bans of billboard advertising increase consumption of spirits and wine, and reduce the demand for beer. The net effect on total alcohol demand is positive prior to 1989, and zero thereafter. One reason for this small effect is that billboards account for only 8 percent of total alcohol advertising. Hence, the elimination of this media would not be expected to substantially affect alcohol consumption, which implies that such bans may be merely symbolic policies.<br>- Prior to 1996, some states instituted bans of price advertising of distilled spirits. The empirical results indicate that states with these bans had lower consumption of spirits and wine, but higher consumption of beer. The net effect on total alcohol consumption was not statistically significant after 1988, which reflects in part substitution effects associated with restrictive laws and regulations.	**INCLUDE.**<br><br>- This study uses a longitudinal sample of 45 states for the period 1982–1997, alcohol consumption per capita were measured in equivalent units of pure alcohol or ethanol (NIAAA, 1999), alcohol prices are obtained from quarterly surveys conducted by the American Chamber of Commerce Researchers Association (ACCRA, 1997; Young and Bielinska-Kwapisz, 2002).

[59]	First author, year, country	General aim	Study design	The policy measure(s) or intervention(s) at hand	Measured policy effects on MLDA	Reflection on policy and other occurrences by authors	Comments
	O'Malley, 1991, USA.	This purpose of this study; 1) to delineate cross-sectional differences in drinking behaviour among US high school seniors and young adults that may be due to variations in recent years in state-level MLDA laws, and 2) to examine the effects of recent changes in MLDA on alcohol consumption and other relevant attitudes and behaviours.	- Analyses used existing data collected by the MTF project, a separate coordinated study used time-series analyses of official reports to examine effects of increases in the MLDA in several states on rates of fatal crashes.<br>- The consumption of alcohol is measured by asking respondents for the number of drinking occasions during the last 30 days and the last two weeks (having five or more drinks in a row).<br>- Time-series results were compared with findings from self-reported data.	- Differing MLDA between 1976 and 1987.	- Higher MLDA were associated with lower levels of alcohol use among high school seniors and recent high school graduates (even after multivariate controls, MLDA remains a significant and substantive predictor of alcohol use).<br>- Combined across all states that increased the MLDA from 18 (to 19, 20 or 21), there was a 13.3% decrease in drinking in the past 30 days.<br>- Alcohol involved highway crashes decline among the 18- to 20-year-old population (sig.), and the present research makes it clear that the decline is directly a result of lower levels of consumption (and because the under-21 group spend less time in bars and taverns when the MLDA is 21).<br>- The lower levels of use persisted into the early 20s, even after all respondents were of legal age and the alcohol is legally accessible.	- Time trends in alcohol use in the constant-21 states are not monotonic. This shows that overall declines in alcohol use were not attributable solely to changes in MLDA. However, some declines appear due to the effect of changes in the laws, because the states that increased their MLDA showed larger declines.<br>- The most common alternative explanation for differences in drinking behavior by high school seniors associated with different MLDA, is that states also differ on other factors, such as standards of religion, anti-alcohol attitudes, or whether states changed their laws voluntarily, or in response to external forces (federal action).	**INCLUDE.**<br><br>- Data from Monitoring the Future (MTF) surveys were used.<br>- Data from the Fatal Accident Reporting System (FARS) were used.

[60]	First author, year, country	General aim	Study design	The policy measure(s) or intervention(s) at hand	Measured policy effects on MLDA	Reflection on policy and other occurrences by authors	Comments
	Plunk, 2013, USA.	This present study uses changes in MLDA laws during the 1970s and 1980s as a natural experiment to investigate the potential impact of permissive MLDA exposure on average alcohol consumption, frequency of drinking, and patterns of binging and more moderate, nonheavy drinking.	- Policy exposure data were paired with alcohol use data, including past-year drinkers born between 1948 and 1972 (n = 24,088). Average daily intake, overall drinking frequency, and frequency of both binge episodes (5+ drinks) and days without a binge episode (nonheavy drinking) for the previous year at the time of interview were tracked for each respondent.<br>- We extend the 2-by-2 analysis to multiple groups and times by including state and birth-year fixed- effects categorical variables to all models.<br>- Multinomial logistic regression was used for the main analyses, wherein we modelled the relative odds of the 3 drinking frequency categories for binge and nonheavy drinking occasions related to permissive MLDA exposure. Logistic, linear, and negative binomial regressions were used for ancillary analyses, to investigate the potential effect of MLDA exposure on drinking status (e.g., lifetime abstainer vs. past-year drinker), average ounces of alcohol per day, and total drinking days, respectively.	A period of MLDA change that occurred between the early 1970s and mid-1980s.	- Exposure to permissive MLDAs was associated with higher odds to report frequent binging (15% higher odds to binge more than once per month, compared with the odds of having had no such occasion) and to report any moderate drinking (sig.) (in other words, that frequent binge drinking becomes more common, while any nonheavy drinking behavior among drinkers becomes less frequent).<br>- These associations were largely driven by men and those who did not attend college (sig.).<br>- Overall drinking frequency and average alcohol consumption were not affected by MLDA exposure (not sig.).	- Exposure to permissive <21 MLDA laws seems to be associated with a certain pattern of drinking behavior that persists into later adulthood, namely, that frequent binge drinking becomes more common, while any nonheavy drinking behavior among drinkers becomes less frequent.<br>- Going to college was associated with a decreased potential MLDA exposure effect. Binge drinking has decreased in the general population, but has remained common on college campuses with the campus environment—characterized by easy access to alcohol coupled with a culture that promotes drinking—likely insulating against policies aimed at restricting underage access to alcohol. We propose that our findings offer support for this campus insulation effect because of ease of alcohol availability, whereby policy exposure for those individuals who attended college would have been substantively different compared with their noncollege peers of the same age.<br>- We should not overly focus on college students when assessing how MLDA affects youth and young adult drinking behavior. While college campuses are arguably conducive to heavy drinking irrespective of policies intended to curb underage alcohol use, in our sample, individuals outside the college environment seem to have been greatly affected by changes in MLDA.<br>- Laws intended to penalize or curb youth possession or consumption in other ways then the ability to legally purchase alcohol were not included in our analyses. There were also no allowances made for smaller jurisdictions within a state that might have had different policies that could further limit access (e.g., dry counties). There could also be other factors influencing lifetime drinking patterns (e.g., cross-state migration).	**INCLUDE.**<br><br>- The 1991–1992 National Longitudinal Alcohol Epidemiological Survey (NLAES).<br>- The 2001–2002 National Epidemiological Survey on Alcohol and Related Conditions (NESARC).

[61]	First author, year, country	General aim	Study design	The policy measure(s) or intervention(s) at hand	Measured policy effects on MLDA	Reflection on policy and other occurrences by authors	Comments
	Smart, 1985, Canada.	The aims of the present study are to compare: (i) changes in the proportions of drinkers and heavy drinkers among both adults and students between 1977 and 1983-1984; and (ii) changes in the frequency of alcohol use for adults and students, and rate of drinking problems among students from 1977 to 1983-1984.	- Adult data were collected in two household-interview studies in 1977 and 1984 with similar questions and sampling methods, designed to produce an approximation of the adult civilian population, 18 years and older. A total of 1772 adults were interviewed in 1977 and 1051 in 1984.<br>- Drinking questions for adults included: drinking in the past year; frequency of drinking; and two questions on heavy drinking, i.e. whether five or more drinks were taken on a single occasion; and, whether the respondent became 'tight' or 'high' because of drinking.<br>- Samples of Ontario students in grades 7,9,11 and 13 have been surveyed every 2 years from 1977 to 1983 on the use of alcohol and drugs, using an anonymous self-administered questionnaire. The questionnaire was completed by 4687 students in the 1977 survey, and by 5835 students in the 1983 survey. In this study, only the data from 1977 and 1983 are compared.<br>- The student-questionnaire included items describing demographic, alcohol use and drug use characteristics. Alcohol use variables included frequency of drinking in the prior year, perceived availability of alcohol and three measures of heavy drinking: the number of times in the prior month drinking alcohol resulted in feeling 'tight', 'drunk' or 'five or more drinks have been consumed on the same occasion'.	- In several jurisdictions, including Ontario (in 1979), the legal drinking age was raised to 19 from 18, after previously being lowered to 18 years in the early 1970%.<br>- Alcohol education programs are introduced in recent years in schools.	- Survey data indicate that the number of drinkers in the adult population increased slightly. There were no increases in the proportions of drinkers at any level of alcohol use and the anticipated reduction in frequent drinkers was not found.<br>- Between 1977 and 1983 there was a significant reduction in the proportion of young drinkers, an increase in infrequent drinkers, and a significant decrease for more frequent drinkers. In addition, fewer students reported drinking five or more drinks on a single occasion in 1983 than in 1977 (sig.).	- The results of this study illustrate the difficulties in clearly attributing a decrease in alcohol consumption to specific preventive efforts. During any period, such as that covered by this study (1977-1983), numerous events impinging on alcohol consumption will have occurred and attributing a change in drinking to any of them exclusively will be difficult.<br>- It is interesting to speculate about why the reduction in drinking, and heavy drinking for young people, in Ontario is greater than for adults. Some general forces, such as the economic recession, should affect both groups and consequently have led to a stabilization in real income in the years from about 1977 to 1983-1984. Currently, important social values emphasizing fitness, exercise and healthy living should also affect both groups.<br>- In 1979 the law was changed in Ontario to increase the drinking age from 18 to 19 years. It may be that the early impact of the law has been sustained. Also, it might have contributed to a change in attitude which views underage drinking more negatively.<br>- Another important development in Ontario has been in alcohol education for schools. Several programs for alcohol education have been developed and evaluated. The evaluation indicates that these programs improve students' knowledge about alcohol, have mixed effects on attitudes and produce decreases in students' reported alcohol use and expected alcohol use.	**INCLUDE.**<br><br>- Preliminary Report of Alcohol and Other Drug Use Among Ontario Students in 1983 and Trends Since 1977, Addiction Research Foundation (student sample).<br>- Alcohol and Drug Use Among Ontario Adults in 1984 and Changes Since 1982, Addiction Research Foundation (adult sample).

[62]	First author, year, country	General aim	Study design	The policy measure(s) or intervention(s) at hand	Measured policy effects on MLDA	Reflection on policy and other occurrences by authors	Comments
	Smith, 1984, USA.	The effects on the raised MLDA in Massachusetts is examined on drinking, drinking and driving, and nonfatal and fatal crash involvement of 16-17-year-olds (teenagers immediately younger than those targeted by the law).	- Data from Massachusetts are compared with those from New York, where the MLDA remained at 18.<br>- A total of 3 years of survey data from the two states and 6 years of data from FARS were used for pre- and post-law comparisons.<br>- An anonymous random digit-dialing telephone survey was conducted in Massachusetts prior to enactment of the law in 1979, asking teenagers about personal characteristics, drinking practices, procurement of alcohol, use of psychoactive drugs, driving after drinking and nonfatal accident involvement. A similar survey was conducted in the New York area (respondents were asked for their drinking behavior and behavior on how they obtained their alcohol comparable to the New York sample).<br>- Twice at yearly intervals following enactment of the law, surveys of similar size were repeated in each state.<br>- Log-linear analysis was used on the survey data.<br>- In addition, FARS data was used for both states from 1976 to 1982 (single-vehicle nighttime accidents were examined separately).<br>- Data were fitted to a log-linear model, an analysis of variance and an analysis of covariance.	The 1979 Massachusetts law raising the MLDA from 18 to 20.	- The findings suggest that raising the MLDA had minimal effects on the drinking behavior of Massachusetts teenagers.<br>- There was a significant decline in Massachusetts in the number of teenagers reporting drinking at a bar/club/restaurant or in an automobile, an increase of teenagers drinking at parties and a decrease in the number of teenagers purchasing alcohol at liquor stores after the raise of the MLDA and compared to New York.<br>- Massachusetts 16-17-year-olds were more likely in each of the 2 post-law years to have had others purchasing alcohol for them (compared to New York).<br>- After the enactment of the law, driving after drinking declined significantly in Massachusetts relative to New York.<br>- Our analysis did not reveal a significant difference in single-vehicle nighttime fatal accidents (and total fatal accidents) among 16-17-year-olds in Massachusetts and New York after the enactment of the law.	- The present findings suggest that the effect of the MLDA changed where these teenagers drink and how they obtain alcohol.<br>- It is interesting that both before and after enactment of the law, 16-17-year-olds in New York drove less frequently after drinking compared to Massachusetts teenagers. Perhaps, this is the result of having a stable (but lower) drinking age in New York over several decades.<br>- Changes in drinking age may offer some reduction in teenage traffic crash involvement, but teenage drinking and teenage driving after drinking remain serious problems, even in states that raise their MLDA.<br>- The present study indicates lawbreaking among young people, perhaps fostering cynicism toward the legislative process and disregard for law enforcement. As long as teenagers are never asked for ID or have others purchase alcohol for them, significant declines in harm are unlikely.	**INCLUDE.**<br><br>- Data from the Fatal Accident Reporting System (FARS).<br><br>- Hingson et al. (1983) examined the first 2 post-law years in Massachusetts [44].

[63]	First author, year, country	General aim	Study design	The policy measure(s) or intervention(s) at hand	Measured policy effects on MLDA	Reflection on policy and other occurrences by authors	Comments
	Subbaraman, 2013, USA.	The primary aim of this study was to evaluate the effects of both raising and lowering the MLDA on per capita ethanol (EtOH) consumption in longer and more accurate time series panel than any previous study.	Generalized least squares model specifications controlling for income, unemployment rates, and population characteristics were implemented using MLDA and aggregate EtOH consumption data from U.S. states from 1950 to 2002.	The effects of both lowering and raising the MLDA.	- Estimates from the full 1950 to 2002 period which include both the lowering and raising of the MLDA indicate that raising the MLDA from 18 to 21 decreased total consumption by 1.51%, beer consumption by 2.31%, and spirits consumption by 1.86% across drinkers of all ages, *implying* substantial changes among underage drinkers (the effects observed relate the MLDA to total alcohol sales across drinkers of all ages, not just among 18- to 20-year-olds).<br>- Results for the later 1976 to 2002 period do corroborate that the MLDA effects observed for the entire 1950 to 2002 period may be largely attributed to raising the MLDA. Increasing the MLDA by 3 years (i.e., from 18 to 21, as was generally done) during this period significantly predicted a 2.25% decrease in per capita total consumption, a 1.8% decrease in beer consumption, and a 3.36% decrease in spirits consumption. These estimates are quite substantial considering that only those under the age of 21 were impacted by the law.	- These results add to the mounting evidence that increasing the MLDA decreases total EtOH consumption as well as alcohol-related harms.<br>- Others have proposed that alcohol policies may interact advantageously. Ponicki and colleagues (2007) reported that raising the MLDA and raising beer taxes independently appeared to reduce fatal motor vehicle accidents in 48 U.S. states from 1975 to 2001 and that increasing the MLDA appeared to reduce proportionately more accidents when taxes were high compared with when taxes are low, suggesting that alcohol policies may work synergistically.	**INCLUDE.**<br><br>- Government and industry beverage-specific sales volume data and used year- and state-specific estimates of mean EtOH content for each beverage type.<br>- MLDA data came from the Prevention Research Center's Statewide Availability Data System.

[64]	**First author, year, country**	**General aim**	**Study design**	**The policy measure(s) or intervention(s) at hand**	**Measured policy effects on MLDA**	**Reflection on policy and other occurrences by authors**	**Comments**
	Buijs (chapter 3, in Van Havere), 2017, Belgium.	The analyses carried out in this chapter were set out to investigate four research questions.<br>- Firstly, it was investigated whether or not the proportion of life-time abstainers, weekly drinkers and binge drinkers evolved differently between adolescents aged under 16 and those older, at the regional and national level.<br>- Secondly, the evolution of life-time and weekly consumption of distilled spirits among adolescents under the age of 18 and those older was investigated at the regional and national level.<br>- A third research question widened the perspective by investigating trends in life-time and weekly alcohol consumption as well as life-time drunkenness among adolescents aged 11-15 years old in 30 countries. This was done in order to analyze the impact of the minimum legal drinking age, as well as a wider array of policies and alcohol affordability, on the outcome measures.<br>- The fourth research question was investigated at the national and international level and concerned the role of socioeconomic status on the different outcome measures.	-	- The impact of the minimum legal drinking age in Belgium. - In December 2009, the Belgian government amended its existing alcohol legislation, to prohibit access to distilled alcoholic beverages to adolescents under the age of 18 and access to all (both distilled and fermented) alcoholic beverages to adolescents under the age of 16. This restriction applied to both the possession by adolescents, as well as alcohol sales by retailers, bars and restaurants.	- Concerning research questions 1 and 2, the analyses at the Flemish and Belgian level showed significant statistical interactions between age and time. This indicates that over the research period, alcohol consumption patterns in the different age groups (under 16 versus older for fermented drinks, under 18 versus older for distilled drinks) have evolved differently, in the sense that the odds of consuming alcohol decreased at a higher pace for those under the respective age limit. This could indicate towards an effect of the law on minimum legal drinking age.<br>- At the international level, similar relations were found between alcohol consumption, age and gender in the group of 11-15 years old. Moreover, the significant time trend, which was observed in Belgium, was also found in the international sample, indicating that overall, alcohol consumption has decreased in the 2002-2014 period. Therefore, no specific evidence was found that supports an impact of the Belgian drinking age legislation on adolescent alcohol consumption.<br>- Furthermore, supplemented analysis on the Belgian MLDA investigating “what works” to reduce alcohol consumption in adolescents younger than 16 years old found mixed results. The minimum legal drinking age was found to be non-significant in relation to life-time and weekly alcohol consumption and positively related to life-time drunkenness indicating that countries with higher minimum legal drinking ages typically have a higher proportion of life-time drunkenness.<br>- When evaluating a wider array of policies that restrict alcohol availability, a significant relation was found between stricter policies and lower odds of weekly alcohol consumption.<br>- The effect of marketing restrictions and affordability changes were also measured. Current marketing restrictions were not found to have a significant effect on alcohol outcome measures.<br>- Affordability changes on the other hand were highly significant with increased affordability being linked to higher odds of alcohol consumption: when alcohol is cheaper, adolescents tend to drink more.	- Although indications towards an effect of the MLDA law were found, it could also be due to other events (e.g. 2008 financial crisis) or increased alcohol prices.<br>- It remains uncertain what the Belgian consumption trend would have looked like in the absence of the 2009 minimum legal drinking age legislation.<br>- It is important to note that in the mixed results in the “what works” analyses, reversed causality could play a role, i.e. that countries which have a higher proportion of life-time drunkenness institute higher minimum legal drinking ages.<br>- An important caveat in the wider array of analyses on policy, is the failure to incorporate enforcement of the above policies into the regression analysis. Such information was not systematically available for the complete international sample.<br>- The results also show that the combination of policy measures (the Total Policy Index represents the mean of the availability index, the affordability index and the marketing restrictions index) can be effective in the reduction of both life time and weekly alcohol consumption.	**INCLUDE.**<br><br>- methodological report describing study design not found.

[65]	First author, year, country	General aim	Study design	The policy measure(s) or intervention(s) at hand	Measured policy effects on MLDA	Reflection on policy and other occurrences by authors	Comments
	Wagenaar, 1982, USA.	The present study assessed the effects on aggregate beer and wine sales of lowering the drinking age and subsequently returning to the original higher drinking age and a mandatory deposit law in the state of Michigan.	- Time series analyses of beverage alcohol distribution in Michigan from 1969 through 1980.<br>- Box-Jenkins-Box-Tiao intervention analysis methods were used, combining iterative univariate Auto-Regressive Integrated Moving Average model identification, estimation, and evaluation techniques with simple transfer functions.	- MLDA, reduced from 21 to 18 for the purchase and consumption in 1972, raised from 18 to 21 for all beverages in 1978 in Michigan.<br>- A prohibition of beer sales in non-returnable containers, implemented in 1978. A deposit of at least 5 cents per container is required on purchase of bottled or canned beer and is refunded when the container is returned.	- A statistically significant temporary increase in aggregate draft beer sales in Michigan is associated with the reduction in legal minimum drinking age from 21 to 18 in 1972. No significant changes in total beer, package beer, or wine sales were associated with the lowered drinking age.<br>- Significant decreases in total beer and package beer distribution and large increases in draft beer distribution occurred in 1979-80, after the legal drinking age was raised from 18 to 21 and a mandatory beverage container deposit law was implemented.<br>- No significant change in wine distribution was observed.	- The significant increase in draft beer distribution in 1972, immediately after the drinking age was reduced from 21 to 18, can be interpreted as a result of the sudden expansion in the population of legal drinkers. A plausible hypothesis is that other factors, unrelated to the lowered drinking age, caused draft beer sales to decrease in 1973.<br>- The substantial decrease in package beer distribution in 1979-80, when the drinking age was raised and nonreturnable containers were banned, also has multiple explanations. Although the raised drinking age may account for a portion of the decrease, the magnitude suggests other factors were operating (e.g., mandatory container deposit laws and associated price jump), affecting the entire population of consumers.<br>- Because both laws were implemented simultaneously, and because other factors expected to influence the demand for beer and wine, such as a major economic recession in Michigan, a causal interpretation of observed aggregate beer consumption changes is difficult. The raised drinking age may account for a portion of the decrease, the magnitude suggests that other factors, affecting the entire population of package beer consumers, were operating.	**INCLUDE.**<br><br>- The Michigan Beer and Wine Wholesalers Association

[66]	First author, year, country	General aim	Study design	The policy measure(s) or intervention(s) at hand	Measured policy effects on MLDA	Reflection on policy and other occurrences by authors	Comments
	Coate, 1987, USA.	This article presents the first set of estimates of the responsiveness of youth alcohol use and motor vehicle death rates to variations in the price of alcohol. In addition, it examines the sensitivity of these two outcome measures to changes in the legal drinking age.	- Two survey datasets are used (NHANES I and II) between May 1971 and June 1974, and between February 1976 and February 1980.<br>- In studying youth demand for alcoholic beverages, we focus on alcohol use by youths aged 16 through 21.<br>- Infrequent and frequent beer consumption was measured by asking for the frequency of drinking beer in a week (no more information is provided).<br>- Data (vehicle accidents) based on a time series of state cross sections for the period from 1975 through 1981.<br>- There age groups were focused on: youths aged 15 through 17, 18 through 20 and 21 through 24.<br>- For both aims, estimates were obtained using variants of multiple regression analysis (controlling for multiple variables, e.g., border-hopping, age, family income, vehicle miles traveled per licensed driver).	- The upward trend in state MLDA for the purchase and consumption of alcoholic beverages.<br>- Increased taxation of alcoholic beverages (mainly focusing on beer).	- Use of alcohol by youths declines when either the price of alcoholic beverages or the MLDA increases. Youth alcohol consumption is inversely related at a statistically significant level to beverage price and MLDA, for both infrequent and frequent/heavy drinkers.<br>- Analysis indicate a statistically significant decline in the motor vehicle accident mortality rate with the effects of a real beer tax for youths aged 15 through 17, 18 through 20 and 21 through 24.<br>- Changes in the MLDA also produced statistically significant inverse effects on accident mortality for youths aged 18 through 20 (affecting the mortality of only those young people who might have legally purchased alcoholic beverages at the lower MLDA).<br>- Positive and significant coefficients of the border variable were obtained for the 18-through-20-year-old cohort.	- If reductions in youth alcohol consumption and motor vehicle accident deaths are desired, the preceding figures suggest that both a uniform MLDA of 21 and an increase in the Federal excise tax rate on beer are effective policies to accomplish this goal.<br>- They also suggest that the tax policy may be more potent than the MLDA policy.<br>- An excise tax increase lowers the death rates of youths between the ages of 15 and 17 and between the ages of 21 and 24. These age groups do not receive the same benefits from a rise in the MLDA, and also reduce fatal crashes involving adult drivers. However, a tax hike may greatly stimulate the demand for illegally produced beer.<br>- Young drivers may respond to increasingly severe penalties for offenses only if the possibility of apprehension and conviction is not trivial. If substantial resources must be allocated to increasing the probability of arrest and conviction, a policy of increased excise taxes may be preferable to or complementary with a system of penalties.	**INCLUDE.**<br><br>- the first and second National Health and Nutrition Examination Surveys (NHANES I and NHANES II).<br>- Data on youth motor vehicle accidents mortality (FARS).

[67]	First author, year, country	General aim	Study design	The policy measure(s) or intervention(s) at hand	Measured policy effects on MLDA	Reflection on policy and other occurrences by authors	Comments
	Wagenaar, 1982, USA.	- The objective of the present study was to assess the relationship between changes in minimum drinking age in Maine and aggregate beer, wine, and distilled spirits consumption.<br>- To help distinguish effects of the drinking age from effects of the container law, beer sales in New Hampshire, the state adjacent to Maine, and beer sales for the United States as a whole were also analyzed.	- The simple interrupted time-series design was used to assess the effects of Maine's lowered and raised drinking age.<br>- Data obtained from the Maine Bureau of Alcoholic Beverages were used to construct monthly time series of the aggregate volumes of beer, wine, and distilled spirits sold in the state of Maine. Similar data for package and draft beer sales in New Hampshire and the United States as a whole were obtained from the U.S. Brewers association, for comparison with Maine.<br>- Long-term trend, seasonal, and other autocorrelation patterns in beverage distribution were controlled using the iterative Auto-Regressive Integrated Moving Average (ARIMA) model identification, estimation, and evaluation analysis strategy of Box and Jenkins.	- Maine raised the drinking age from 18 to 20 for all alcoholic beverages in 1977.<br>- Maine implemented a mandatory container law in 1978.	- Controlling for the effects of long-term trends and regular seasonal patterns, the present study found significant changes in aggregate beer sales concomitant with modifications in the legal minimum drinking age (in Maine, an increase in drinking age from 18 to 20 was associated with a significant decrease in beer sales).<br>- In both states examined, beer, the beverage of choice among young drinkers, was the beverage category affected by changes in drinking age.	- The most reasonable conclusion is that reduced beer sales in Maine in the late 1970s were due to a combination of reductions in beer consumption among young drinkers after availability was reduced by raising the legal purchase age, and reductions in beer consumption among all drinkers as a result of higher prices that followed implementation of a beverage container deposit law.<br>- Results of analyses of nationwide beer sales for comparison with the state-specific analyses revealed that beer sales changes associated with modifications in legal drinking age in New Hampshire and Maine were not a reflection of national trends, strengthening the argument that observed relationships between drinking age and beer sales reflect a causal effect of the drinking age.<br>- While it appears that reduced beer sales in Maine were not a result of increased cross-border purchase of beer by Maine residents (to New Hampshire), the container deposit law and resulting price increases may have caused a significant reduction in beer consumption.<br>- A final consideration in interpreting the identified decrease in beer sales after the drinking age was raised is that 18-19-year-olds are only a small proportion of the total population of drinkers. Therefore, 18-19-year-olds would have had to dramatically reduce their consumption to account for the 1,114-kiloliter-per-month decrease. These calculations illustrate the implausibility of attributing the entire decrease in beer sales beginning in late 1977-early 1978 to the increase in legal minimum drinking age.	**INCLUDE.**<br><br>- The Maine Bureau of Alcoholic Beverages.<br>- The U.S. Brewers association.

[68]	**First author, year, country**	**General aim**	**Study design**	**The policy measure(s) or intervention(s) at hand**	**Measured policy effects on MLDA**	**Reflection on policy and other occurrences by authors**	**Comments**
	Williams, 1988, USA.	Utilizing survey data for one year before, one year after and 3 years after New York raised its purchase age from 18 to 19 years, this study examines the short-term alcohol purchasing and long-term purchasing and consumption patterns of 16- to 20-year-olds.	A three-stage, stratified proportionate random sampling design was used to select approximately 1,800 16- to 20-year-old New Yorkers living in households.<br>- Anonymous telephone interviews were conducted in 1982 before New York raised its minimum legal purchase age for alcoholic beverages to 19 years. A second sample was interviewed in 1983, approximately one year after the new purchase age law and a third sample utilizing the same design was interviewed in 1985, approximately 3 years after the new purchase age went into effect.<br>- Alcohol purchasing was determined by analyzing survey questions that asked respondents, "In the past 4 weeks did you purchase any beer (liquor) in bars, clubs or restaurants (stores)?" Consumption prevalence levels were established by analyzing survey questions that asked respondents: "How many days in the past 4 weeks did you have a drink?<br>- All comparisons are tested using a proportions test which yields a z-score by comparing the appropriate prevalence measures.	The raise of the MLDA of the state of New York from 18 to 19 in 1982.	- In 1983, the year following the purchase age increase, purchasing prevalence measures for 18- year-olds were significantly lower when compared to 19- and 20-year-old measures for all categories of purchasing.<br>- Eighteen-year-old purchasing measures decreased significantly for all categories from 1982 to 1983.<br>- On the long term, for all purchasing categories, 18-year-old prevalence was significantly lower than the comparable measure for 19- and 20-year-olds in 1985.<br>- Consumption measures of eighteen-year-olds were lower than measures for 19- and 20- year-olds for all measures, and the differences were significant in seven out of 10 comparisons.	- New York's purchase age increase resulted in significant short-term decreases for reported alcohol purchasing by the age group directly affected by the law change and adds support to earlier findings related to alcohol consumption.<br>- The long-term influence of the higher purchase age was maintained since 18-year-olds continued to show significantly lower prevalence rates of alcohol consumption and purchasing in 1985 when compared to 19- and 20- year-olds.<br>- The short-term changes and patterns for alcohol purchasing among 18-year-olds compared to age groups slightly younger and older strongly suggested a purchase-age-specific effect as opposed to a general youth phenomenon.<br>- Age-specific influence was operating on *purchasing* by 16-, 17- and 18-year-olds and was not evident for reported *consumption*. One such potential influence is the implementation of photo licenses. In 1984 all new licenses as well as all renewals were in the form of a special coated card to prevent tampering and included a photograph of the driver's face. This may have further diminished the capacity of illegal purchasers to obtain alcohol.	**INCLUDE.**<br><br>-

[69]	**First author, year, country**	**General aim**	**Study design**	**The policy measure(s) or intervention(s) at hand**	**Measured policy effects on MLDA**	**Reflection on policy and other occurrences by authors**	**Comments**
	Dee, 1999, USA.	This empirical study presents new evidence on the efficacy of state alcohol policies (i.e. excise taxes on beer and minimum legal drinking ages) in reducing the prevalence of teen drinking and its key related outcome, youth traffic fatalities.	- The evaluations presented here are based on data set that consists of pooled cross-sections from the 1977–92 Monitoring the Future (MTF) surveys of high school seniors.<br>- Consumption behavior was categorized into ‘‘drinkers’’ (any drink of alcohol in the last month), ‘‘moderate drinkers’’ (10 or more drinks of alcohol in the past month) and ‘‘heavy drinkers’’ (5 or more drinks in a row sometime in the last 2 weeks).<br>- The other key evidence linking state alcohol policies and teen drinking by evaluating empirical models of youth traffic fatalities are based on reduced-form models of youth traffic fatalities based on a relatively long panel (1977–92) of annual state-level data.	Excise taxes on beer and minimum legal drinking ages between 1977-92.	- Beer taxes have a relatively small and statistically insignificant impact on teen drinking. Also, the empirical models of traffic fatalities demonstrated that the conventional link between beer taxes and youth traffic fatalities is not a robust one.<br>- The results of these estimations indicate that the movement to higher minimum legal drinking ages substantially reduced teen drinking and led to large and statistically significant reductions in youth traffic fatalities (estimates suggest that the movement to higher MLDA reduced heavy teen drinking by at least 8% and traffic fatalities by at least 9%).	The success of higher minimum legal drinking ages in reducing the prevalence and social consequences of teen drinking suggests that current and future efforts to curb teen alcohol use should recognize the probable efficacy of policies that further increase the non-pecuniary cost to teens of acquiring alcohol.	**INCLUDE.**<br><br>- Data from Monitoring the Future (MTF) surveys.<br>- Data from Fatal Accident Reporting System (FARS).

[70]	First author, year, country	General aim	Study design	The policy measure(s) or intervention(s) at hand	Measured policy effects on MLDA	Reflection on policy and other occurrences by authors	Comments
	Kaestner, 2000, USA.	This study reexamines the effect of MLDA laws on youth alcohol consumption.	- The data of the NLSY (longitudinal survey) are used for 1982 and 1985, using respondents between the ages of 17 and 21 in 1982 and respondents between the ages of 20 and 21 in 1985.<br>- Alcohol consumption was measured using three variables: 1) the number of occasions in the past 30 days that the respondent had six or more drinks, 2) the number of days in the past week the respondent drank, and 3) the number of beers the respondent consumed in the last week.<br>- The effects of MLDA on alcohol consumption are reexamined by using two quasi-experimental statistical methodologies (a OLS (ordinary least squares) method and a 'difference-in-differences-in-differences (DDD) method is used), that control for unmeasured factors.<br>- Separate models are estimated by gender to investigate whether the effect of MLDA differs by sex.	Changes in MLDA in the U.S.	- Estimates obtained using OLS methods and a pooled sample of men and woman indicated that raising the MLDA reduced alcohol consumption.<br>- However, when separating for sex, OLS estimates were inconsistent.<br>- All the estimates from DDD regression models indicated that MLDA had no statistically significant effect on youth alcohol consumption.	- The estimates of the effect of MLDA on youth alcohol consumption presented in this article lack a degree of robustness necessary to conclude that MLDA effectively reduce alcohol consumption.<br>- The lack of statistical significance of the fixed-effects and DDD estimates are consistent with other studies that control for unmeasured state factors that affect alcohol consumption.<br>- How can MLDA affect driving fatalities if not by reducing alcohol consumption? One potential explanation of this paradox is that MLDA have a larger effect on drinking and driving than on consumption. MLDA may affect where and how youths drink and not how much they drink (e.g., keeping youths out of bars and restaurants, not other places they drink).	**INCLUDE.**<br><br>- The National Longitudinal Survey of Youth.

[71]	**First author, year, country**	**General aim**	**Study design**	**The policy measure(s) or intervention(s) at hand**	**Measured policy effects on MLDA**	**Reflection on policy and other occurrences by authors**	**Comments**
	Coate, 1988, USA.	A primary purpose of this article is to investigate the sensitivity of alcoholic beverage consumption, particularly excessive consumption, to price among sixteen- through twenty-one-year-olds in the United States. We also examine the effect of an increase in the legal drinking age on youth alcohol use.	- Our empirical research is based on surveys (NHANES II) conducted between 1976 and 1980, investigating substantial differences in MLDA among states in the period of NHANES II and on substantial differences in the prices of alcoholic beverages among states that were due primarily to differences in state excise tax rates on these beverages.<br>- Alcohol consumption was measures asking for the number of drinking occasions per week in the past three months (4-7 times a week, 1-3 times a week, less than once a week, or never).<br>- We have limited our demand function estimates to sixteen- through twenty-one-year-olds because of our interest in the sensitivity of alcohol consumption of older youths to price and to legal drinking age.<br>- Maximum likelihood estimates of multinomial logit frequency equations are used.	- Changes in (beer and liquor) MLDA in the U.S.<br>- Federal excise tax (price) on alcohol beverages.	- The frequency of the consumption of beer is inversely related to the real price of beer and to the minimum legal age for its purchase and consumption (sig.).<br>- The fractions of youths who consume beer fairly frequently (one to three times a week) and frequently (four to seven times a week) fall more in absolute or percentage terms than the fraction of infrequent drinkers when price or the drinking age rises.<br>- (an estimate) A federal policy that simultaneously taxes would have reduced the number of youths who drink beer frequently (approximately 11 percent of all youths) by 32 percent during the period of NHANES II and would have reduced the number of fairly frequent beer drinkers (approximately 28 percent of all youths) by 24 percent.<br>- (an estimate) The enactment of a minimum uniform drinking age of twenty-one in all states would have reduced the number of frequent drinkers by 28 percent and the number of fairly frequent drinkers by 11 percent.	- These figures (estimations of taxes and MLDA at 21) suggest that the tax policy may be more potent than the drinking age policy.<br>- Excise tax hikes impose welfare costs on all segments of the population, while a drinking age policy is targeted at the group in the population that accounts for a disproportionate share of motor vehicle accidents and deaths.<br>- The enforcement and administrative costs associated with a uniform MLDA of 21 may exceed those associated with the tax policy.<br>- An excise tax increase may reduce excessive alcohol consumption by adults as well as by youths.<br>- A substantial tax hike may stimulate the demand for illegally produced beer.<br>- The optimal way for a society to deter offenses is via a system of severe and fairly certain punishments. In the case of drunk driving, loss of driving privileges, mandatory community service, enrollment in alcohol rehabilitation programs, and prison sentences for repeat offenders are possible.<br>- Youthful drunk drivers may respond to an increase in the penalty of this offense only if the probabilities of apprehension and conviction are nontrivial.	**INCLUDE.**<br><br>- The second National Health and Nutrition Examination Survey (NHANES II), which was conducted by the National Center for Health Statistics (NCHS).

[72]	First author, year, country	General aim	Study design	The policy measure(s) or intervention(s) at hand	Measured policy effects on MLDA	Reflection on policy and other occurrences by authors	Comments
	Kaestner, 2009, USA.	We examine whether adult alcohol consumption and traffic fatalities (adults with an average age of 35) are associated with the legal drinking environment when a person was between the ages of 18 and 20.	- The two outcomes we examine are alcohol use and traffic fatalities.<br>- Data on alcohol use comes from the BRFSS, from the years 1995 to 2005. We limited the sample to individuals from those survey years born between 1960 and 1975 because these individuals were in their late teens in the early to late 80s when most states raised the MLDA to 21.<br>- The BRFSS contains self-reported information about alcohol use in the past month. Several measures were constructed: 1) the proportion of days drank alcohol in past month, 2) number of drinks in past month, 3) whether binge drank in past months (5 or more drinks at one time), and 4) the proportion of days binge drank in past month.<br>- Data on fatal traffic accidents were taken from FARS, from the years 1995 to 2006, focusing on nighttime fatalities.<br>- Regression analyses were conducted.	- Changes in MLDA in the U.S.<br>- Federal and state beer taxes.	- Men who grew up in an environment that allowed them to drink between the ages of 18 and 20 reported drinking a greater number of drinks in the past month (sig.) and more episodes of binge drinking in the past month (sig.) than men who grew up in an environment that prohibited them from drinking during late adolescence. Magnitudes of the associations were non-trivial suggesting differences of 20 to 30 percent in these measures of alcohol use, and even larger for low-educated males.<br>- Again, for males, traffic fatalities (per 100,000) of those who were always able to legally drink between ages 18 and 20 are between 2.6 (9 percent) and 3.1 (11 percent) higher than for those who were never able to legally drink between ages 18 and 20 (sig.).<br>- A similar result is obtained in the case of evening accidents (also for males); those always able to legally drink had approximately 10 percent more accidents than those never able to legally drink (sig.).<br>- There were no statistically significant or practically important associations between the legal drinking environment when young and adult female alcohol consumption and driving fatalities.	To summarize, we found that a minimum legal drinking age of 21, versus a minimum age of 18, is associated with a 20 to 30 percent reduction in adult male alcohol use and a ten percent reduction in fatal traffic accidents with adult male drivers. These estimates support arguments made by those in favor of keeping the minimum legal drinking age at 21 that such laws have long term, beneficial consequences. Importantly, the evidence we present is a direct assessment of that argument and does not rely on hypotheses of long-term effects of such laws derived from neurobiological studies of how alcohol use affects adolescent brain development.	**INCLUDE.**<br><br>- Behavioral Risk Factor Surveillance System (BRFSS).<br>- Data on fatal traffic accidents were taken from the Fatality Analysis Reporting System (FARS).

[73]	First author, year, country	General aim	Study design	The policy measure(s) or intervention(s) at hand	Measured policy effects on MLDA	Reflection on policy and other occurrences by authors	Comments
	Ornstein, 1985, USA.	- This article tests the social marketing effectiveness of alcohol control laws designed to reduce the consumption of alcoholic beverages using state-level historical data.	- We postulated a multiplicative demand model because we expected the economic, sociodemographic and regulatory variables to have nonlinear, interactive effects on consumption.<br>- The sample was constructed from observations made on 50 states plus the District of Columbia during 1974 – 1978.<br>- Parameter estimation was done using ordinary least squares (OLS), ordinary least squares with dummy variables (OLSDV) and variance components (VC) methods.	- Retail availability (MLDA, legality of Sunday sales, legality of drug store and grocery store sales and the existence of local option votes in dry vs. wet counties).<br>- Price controls (mandatory resale price maintenance).<br>- Advertising restrictions (state control of print and outdoor advertising).<br>- Ownership control (monopoly or license state).	- The main determinants of interstate differences in per capita consumption of *distilled spirits* are price, income, and interstate travel – not differences in alcohol-control laws.<br>- Control laws are either unrelated to distilled spirits consumption, as in the cases of MLDA and Sunday sales, or are related but with very low elasticities, as in the cases of resale price maintenance and print/billboard price advertising.<br>- In the case of *beer*, the primary influence on demand is the youthfulness of the population, control laws with the strongest relationship to beer are MLDA and Sunday sales.<br>- Price and income are far more inelastic for beer compared to distilled spirits.	- Control laws affecting price have the greatest impact on consumption.<br>- Control laws influencing price will have a relatively lesser effect on beer consumption than on spirits consumption.<br>- Regarding availability of alcohol, for both beer and distilled spirits, the influence of control measures is small relative to that of sociodemographic and economic variables that affect consumers overall attitudes toward drinking.	**INCLUDE.**<br><br>- Distilled Spirit Council of the United States – multiple reports, summary's and reviews.

[74]	First author, year, country	General aim	Study design	The policy measure(s) or intervention(s) at hand	Measured policy effects on MLDA	Reflection on policy and other occurrences by authors	Comments
	Zhang, 2011, USA.	This study examined: (1) the relationship between alcohol-control environment during young women's early childbearing years, indicated by the MLDA laws at their 14th birthday, and infant health indicators; (2) whether changes in birth composition, drinking behaviors, or formation of drinking habits contributed to change in infant health; and (3) whether racial differences relative to the above issues existed.	- State- and year-fixed-effects models are used to analyze the relationship between MLDA, drinking behaviors, and birth outcomes.<br>- Regarding drinking behavior, two variables were constructed accordingly: drinking prevalence and the incidence of at least five alcoholic beverages per occasion (i.e., binge drinking) during the previous month.<br>- We studied the effects of different MLDA (age 18, 19, 20, or 21 years) when potential mothers were 14 years old by merging two population-based datasets between 1985 and 2002.	- State-specific variations of MLDA (18, 19, 20 or 21) when mothers were 14 years old.<br>- Real beer taxes (federal plus state) were added to the analyses, since beer taxes changed between 1970 and 1989.	- A MLDA of 18 years old (when potential mothers were 14 years old) increased the prevalence of low birth weight, low Apgar scores, and premature births (all sig.).<br>- Effects were stronger among children born to black women compared with white women (sig.).<br>- An MLDA at age 21 years decreased the probability of alcohol drinking among black women by 2–8% points (sig.).<br>- Those who lived in a state where the MLDA was age-18, were 2.2 percentage points (sig.) more likely to binge than those who lived in states with an older MLDA. Exposure to age-20 or -21 MLDA reduced the binge drinking by about 6.6 percentage points (sig.).	- These results indicate that the MLDA changed drinking behaviors among white and black women in a different way. For black women, a movement away from age-18 as the drinking age decreased overall drinking probability to a relatively modest degree and decreased binge drinking substantially more. Apparently, an increase in the legal drinking age modified the formation of habitual drinking by effectively reducing the availability of alcoholic beverages. For white women, an older MLDA modestly reduced overall drinking, but not binge drinking. This suggested that it was moderate white drinkers rather than heavy ones who were sensitive to changes in MLDA.<br>- Not all policies, such as expansion of Medicaid for pregnant women, were accounted for in this study. In addition, some other factors like insurance status, utilization and frequency of pre-natal care may influence infant health, but were not included in the analysis. Hence, the omitted variable bias may still exist.	**INCLUDE.**<br><br>- The Vital Statistics Natality Detailed Files from 1970 to 1992.<br>- The Behavioral Risk Factor Surveillance System from 1985 to 2002.<br>- The Behavioral Risk Factor Surveillance System (BRFSS) from 1985 to 2002 to investigate women's health behaviors.

[75]	First author, year, country	General aim	Study design	The policy measure(s) or intervention(s) at hand	Measured policy effects on MLDA	Reflection on policy and other occurrences by authors	Comments
	Lillis, 1987, USA.	Assessing the impact of increasing the legal drinking age on drinking and driving behavior.	- A multiple-indicator, pre-comparison and post-comparison design was used.<br>- Changes in indicators for 18-year-olds were compared with changes for other age groups.<br>- Three independent data sources related to drinking and driving were utilized: 1) the accident records submitted by police agencies to the Department of Motor Vehicles (data from two periods was used; 12/4/81 to 12/3/82 and 12/4/82 to 12/3/83), 2) arrest records maintained by the New York State Police, and 3) self-reported drinking-and-driving data (measuring 'driving after feeling the effects of alcohol' at least once in the 28 days prior to the survey), and questions regarding the purchase behavior of specific beverages (i.e., beer, wine or liquor) in the preceding 28 days at specific locations (i.e., bars of stores)) culled from the Youth Alcohol Survey (using telephone interviews comprising 2000 16- to 20-year-old New Yorkers conducted in 1982 (before the increase) and 1983 (after the increase)).	The legal minimum purchase age for alcohol beverages in New York State that increased from 18 to 19 years old in 1982.	- Analyses of three independent indicators of drinking and driving provide strong support for the hypothesis that the increase in the legal purchase age in New York State from 18 to 19 years resulted in decreases in drinking-and-driving behavior by 18-year-olds.<br>- Changes in the rates of drinking-and-driving indicators showed a 21% decrease in fatal and injury-causing crashes and a 39% decrease in fatal crashes for 18-year-olds (sig.).<br>- Non-crash DWI arrests of 18-year-old drivers decreased 35% (sig.).<br>- A 46% decrease in self-reported drinking and driving from the survey were found for 18-year-olds (sig.) following the increase in the purchase age to 19.<br>- For all measures, the rate of decrease for 18-year-olds was significantly greater than the rate of decrease for drivers 21 years old and older.<br>- After the increase, the rate of beer purchasing by 18-year-olds was significantly smaller than the rate for 19- or 20-year-olds (33% vs. 51% and 47%, respectively), and decreased significantly for 18-year-olds following the increase (from 52% to 33%, respectively).	- The findings support the theory that the 19-year-old purchase age in New York has had a major immediate impact on 18-year-olds in the state.<br>- Opponents of purchase-age increases have argued that any changes in drinking-driving crash rates are due to the STOP-DWI legislation and programs, and not to purchase age policies. However, none of the laws and few programs have been aimed specifically at young drivers (e.g., 18-year-olds). Efforts of STOP-DWI cannot explain the differences between the rate of change for 18-year-olds.	INCLUDE.<br><br>- The accident records submitted by police agencies to the Department of Motor Vehicles.<br>- Arrest records maintained by the New York State Police.<br>- The Youth Alcohol Survey.

[76]	First author, year, country	General aim	Study design	The policy measure(s) or intervention(s) at hand	Measured policy effects on MLDA	Reflection on policy and other occurrences by authors	Comments
	Wilkinson, 1987, USA.	This study compares the relative effectiveness of multiple policies in terms of reduced fatalities, so policy makers are able to allocate financial and political resources more effectively.	- Data were collected for all 50 states and the District of Columbia for the year 1976-1980. The final sample consists of 221 observations.<br>- State is the level of analysis in this paper, individual characteristics are subsumed into state-level categories.<br>- Equations are assumed having multiplicative form, therefore natural logs of continuous variables were used in regression analyses on a) annual fatalities per 1000 persons >15 years and b) annual consumption of ethanol in gallons per person >15 years.	- These policies include: 1) raising the minimum legal drinking age, 2) increasing legal sanctions and enforcement, and 3) increasing the price and 4) reducing the availability of alcoholic beverages.	- The results indicate that increasing the minimum legal drinking age (MLDA) reduces alcohol consumption. Raising the age from 18 to 19 or 20 has a negligible effect. However, if the limit is raised to 21, alcohol consumption falls by 4 percent (sig.).<br>- Estimation of the parameters indicates that roadway and driver characteristics are important determinants of fatalities.<br>- Alcohol consumption is significant, indicating that fatalities can be reduced by reducing alcohol consumption.<br>- An important result of this paper is that, at current levels, drunken driving deterrence policies appear to have no marginal effect on the demand for alcohol or the level of fatalities.	- An alternative explanation of the positive coefficients for the deterrence variables that these variables are endogenous and the causality runs from fatalities to deterrence. If the police and courts increase the likelihood of arrest and conviction in response to an increase in fatalities or alcohol consumption or both, then positive coefficients on these variables would be possible. Perhaps, the deterrent treat is too low to be credible for many drinking drivers.<br>- That deterrence policies appear to have no marginal effects should not be interpreted as a suggestion to eliminate enforcement of anti-drunk driving statutes. Many people may be deterred or otherwise decide not to drink and drive because of current policies. Rather the results suggest that adding resources to their enforcement will not deter more persons from drunken driving. The resources would be more effective if used otherwise.<br>- It is important to realize that large effects do not necessarily imply the "best" policies. The optimal strategy is to use policies up to the point where their marginal cost is just equal to their marginal benefit. Every policy imposes costs. These costs may be direct costs, such as policemen's salaries, or indirect costs such as the loss of benefits of programs displaced by drunken driving policies or the reduced welfare of those who become too young to drink legally. Because these costs will vary across localities, the optimal policy mix will vary also.	**INCLUDE.**<br><br>-

[77]	First author, year, country	General aim	Study design	The policy measure(s) or intervention(s) at hand	Measured policy effects on MLDA	Reflection on policy and other occurrences by authors	Comments
	Baccini, 2014, Europe.	This paper focuses on the association between alcohol consumption and the introduction of five control policy measures (within the AMPHORA project) in 12 European countries, considering contextual socioeconomic factors.	Using data from the AMPHORA project (time series and aggregate data), first, country-specific analyses were performed in a regression model (using a logarithmic scale) to obtain country-specific estimates of the association between each kind of policy and alcohol consumption, adjusted for contextual unplanned factors. Second, the country-specific estimates were compared and combined in a random-effects meta-analysis.	The five defined typologies were the following:<br>- Restrictive advertising policies: policies that introduced limitations in alcohol advertising.<br>- Restrictive availability policies: restrictive policies acting on licensing rules and trading hour sales.<br>- Permissive availability policies: permissive policies acting on licensing rules and trading hour sales.<br>- Changes of the legal minimum age to buy alcohol.<br>- Changes of the legal blood alcohol concentration (BAC) limit when driving.	- The present analysis showed that the association between policy measures and total alcohol consumption in Europe during the study period was very heterogeneous.<br>- Policies on restricting alcohol availability and on enhancing the minimum age for alcohol purchase appeared to be related to decreasing alcohol consumption.<br>- Increasing the minimum age limit by 1 year was associated with a decrease of 9.8% in alcohol consumption (90% CI: 15.4%, 4.2%).	- Part of the heterogeneity found between countries could be explained by the fact that policies of the same typology have been introduced in very different calendar periods, depending on the country.<br>- In interpreting the results, we should account that sometimes, interventions of different typology have been implemented within a few years. In addition, sometimes the selected policies consisted of a set of measures, and we classified them, according to the defined partition, on the basis of the measure that appeared predominant. In these cases, a great effect may be estimated due to the complex intervention implemented in that year, but the estimated association should not be completely attributed to a specific policy typology.	**INCLUDE.**<br><br>- The AMPHORA project examined the pattern over time of alcohol consumption, as related to policy measures and time-varying unplanned factors, in 12 European countries during 1960s to 2000s.

[78]	First author, year, country	General aim	Study design	The policy measure(s) or intervention(s) at hand	Measured policy effects on MLDA	Reflection on policy and other occurrences by authors	Comments
	Carpenter, 2007, USA.	This study provides the first historical comparative analysis of the effects of Minimum Legal Drinking Ages (MLDA), beer taxes, and “Zero Tolerance” (ZT) underage drunk driving laws on the drinking behaviors of high school seniors using confidential area-identified data from the 1976-2003 waves of the Monitoring the Future (MTF) Surveys.	- Confidential area-identified data from the 1976-2003 waves of the MTF surveys were used. The effects of all three policies were estimated in reduced form models for each outcome variable (similar to the standard difference-in-differences approach).<br>- Consumption is measured on past month alcohol use (called ‘drinker’), and on the consumption of five or more drinks in a row the last two weeks (called ‘heavy episodic drinker’).	Relevant variation in each of the policies occurs in different decades: the majority of the changes in state MLDA took place in the late 1970s and early 1980s, adoption of tough (ZT) drunk driving laws in the 1980s and 1990s and various direct price increases, including a doubling of the federal excise tax on beer, occurred around 1991.	- Strong evidence is found that exposure to a MLDA of 18 was associated with large and statistically significant increases in drinking participation and heavy episodic drinking by high school seniors, on the order of 2-3 percentage points.<br>- Our findings confirm that nationwide increases in the MLDA in the late 1970s and 1980s and adoption of ZT laws in the 1990s both significantly reduced alcohol consumption by high school seniors with larger effects for the MLDA than for ZT laws.<br>- More specifically (related to the MLDA-effect), we estimated that nationwide increases in the MLDA (i.e. movements away from the most permissive age of 18) reduced youth drinking by about four percent relative to pre-existing levels.	- ZT laws were adopted in a period when states had already set their MLDA at 21. This makes the reductions in teen drinking and heavy episodic consumption associated with ZT laws all the more remarkable, since underage youths who drank in the 1990s were already in violation of laws concerning the MLDA. This suggests that MLDA laws were not completely effective and that youths remain quite sensitive to other interventions related to, for example, their driving privileges or to the money price of alcohol.<br>- Underage drinking rates remain at or above 50 percent, even after all states have made such activity illegal. While our estimates provide strong evidence that youth drinking behavior is responsive to a variety of types of government interventions, they also tell a cautionary tale concerning the limits of regulation. Further research on the competing roles of family, peer, and state influences in youth substance use would be particularly useful in this regard.<br>- In future policy, further toughen state drunk driving laws targeted at youths (such as imposing harsher penalties, longer license revocations, and/or requiring all states to set the ZT limit at .00 BAC (i.e. “not a drop”)), and adopting additional sizable beer tax increases could reduce youth drinking further. Because nearly all high school seniors are below the age of 21, further increases in the legal drinking age are unlikely to directly affect consumption.	**INCLUDE.**<br><br>- Confidential area-identified data from the 1976-2003 waves of the Monitoring the Future (MTF) surveys were used.

[79]	**First author, year, country**	**General aim**	**Study design**	**The policy measure(s) or intervention(s) at hand**	**Measured policy effects on MLDA**	**Reflection on policy and other occurrences by authors**	**Comments**
	Cheng, 2019, USA.	To investigate the potential primary prevention effects on precocious drug use and to clarify lag-time issues, we estimated incidence rates for specified intervals anticipating and lagging after drug policy enactment.	- The study population is 12–23-year-old US civilians.<br>- Estimates are from 30 community samples drawn to be nationally representative for the US National Surveys on Drug Use and Health 1979–2015.<br>- Estimates were year-by-year annual incidence rates for alcohol drinking and tobacco smoking by 12–23-year-olds, age by age.<br>- These items assessed the participant's age when they had their first drink or smoked their first cigarette, as well as recency of use, and lifetime history of use. In this study, incidence was conceptualized as the number of newly incident users who started drinking or smoking at a specific age rising from the "at risk" population comprised of never users assessed at a specific age and newly incident users.<br>- Meta-regressions estimate age-specific incidence over time.	Two separate US nation-level policy changes are the focus, clearly intended to have beneficial primary prevention effects on precocious or otherwise underage drug use: The National Minimum Drinking Age Act (NMDAA) and the Synar amendment (prohibited sales or distribution of tobacco products to individuals before the 18th birthday).	- In general, once nation-level policies affecting drug sales to minors are enacted, one might have to wait almost a decade before seeing tangible policy effects on drug use incidence rates.<br>- Declines in incidence of adolescent alcohol drinking after the onset of the National Minimum Drinking Age Act (NMDAA) appear as a continuation of a decreasing trend that started before the NMDAA. The rise among 21-year-olds did not emerge until approximately 10 years after NMDAA. Two decades after NMDAA, a second decline occurred among adolescents.<br>*- Eight years after the Synar amendment enactment, evidence of reduced smoking incidence started to emerge. Among 18-year-old's, a slight increase in tobacco smoking incidence occurred about 10 years after the Synar amendment.*	Many changes related to alcohol drinking and tobacco smoking co-occurred with NMDAA and Synar. For example, there were numerous nation-, state-, and local-level policies and movements, including excise taxes on tobacco and alcohol products, state-level MLDA or minimum legal tobacco purchasing age, mass media campaigns, and grassroots movements throughout the study period, as well as variations in the implementation of state-level policies over time. The observed changes are results of the national NMDAA and Synar combined with these various factors.	**INCLUDE.**<br><br>- Data was used from National Household Survey on Drug Abuse (NHSDA), which changed to National Survey on Drug Use and Health (NSDUH) in 2002.

[80]	First author, year, country	General aim	Study design	The policy measure(s) or intervention(s) at hand	Measured policy effects on MLDA	Reflection on policy and other occurrences by authors	Comments
	Dee, 2003, USA.	This study examines the effects of teen alcohol use and availability on educational attainment, demonstrating effects of changes in MLDA on drinking and educational attainment.	- The first section of this article uses the NELS-88 to establish an empirical baseline: teens who drink (measured as past month alcohol use called 'drinkers', drinking 10 or more drinks within the past month called 'moderate drinkers, and the consumption of five or more drinks in a row the last two weeks is called 'heavy drinker') are less likely to complete high school and less likely to enter college using linear probability estimates / weighted OLS regressions.<br>- Increases in MLDA during the 1980s are used as exogenous determinants of teen drinking (using data from MTF-surveys in Weighted Least Squares Estimates).<br>- The last section tests whether educational attainment within states increased after the MLDAs were increased, using PUMS data and Reduced-Form and Two-Sample Instrumental Variables Estimates.	This study used the increases in MLDA during the 1980s and state excise taxes on beer.	- Teens who drink are less likely to complete high school and less likely to enter college.<br>- We demonstrate that teens who faced an MLDA of 18 were substantially more likely to drink than teens who faced a higher drinking age.<br>- Teen exposure to an MLDA of 18 had small and statistically insignificant effects on indicators for high school completion, college entrance, and college persistence.	- Estimates of the policy determinants of teen drinking demonstrated that, though the cross-state variation in beer taxes correlates with teen drinking, the within-state variation does not. Therefore, frequent recommendations for increased beer taxes appear to be based on what may only be a spurious correlation generated by unobserved state heterogeneity.<br>- The within-state increases in MLDA, which significantly affected all levels of teen drinking, provided a source of exogenous variation for identifying the true effect of teen alcohol consumption on educational attainment.<br>- By focusing on the magnitudes of the links among alcohol policy, teen drinking, and educational attainment, this identification strategy has also underscored the fact that alcohol control policies could at best be a fairly weak policy lever for improving the levels of schooling among youth.<br>- Other policy interventions with larger and more direct links to the schooling decisions made by teens should be able to promote a greater improvement in the accumulation of human capital.	**INCLUDE.**<br><br>- National Education Longitudinal Study of 1988 (NELS-88)<br>- Data from the 1977–92 Monitoring the Future (MTF) surveys<br>- Data from the 1960–69 birth cohorts in the Census Bureau's 1990 5% Public-Use Microdata Sample (PUMS).

[81]	First author, year, country	General aim	Study design	The policy measure(s) or intervention(s) at hand	Measured policy effects on MLDA	Reflection on policy and other occurrences by authors	Comments
	DiNardo, 1992, USA.	This paper analyzes the impact of increases in the minimum drinking age on the prevalence of alcohol and marijuana consumption among high school seniors in the United States.	- The empirical analysis is based on a large sample of students from 43 states over the years 1980-1989, using log-linear model estimates and a structural probit model estimate.<br>- Consumption is measured as the past 30 day alcohol (and marijuana) participation.	This study used the increases in MLDA between 1980-89 in 43 sample states.	- We find that higher minimum drinking ages reduces the prevalence of alcohol consumption.<br>- Increased legal minimum drinking ages had the unintended consequence of increasing the prevalence of marijuana consumption. The unintended consequence is attributable to standard substitution effects.	- An increased drinking age helps create a climate of societal disapproval for all drug use, not only alcohol. In the case of marijuana, this change in societal 'climate' is not sufficient to offset the large substitution induced by the decreased prevalence of alcohol consumption.	**INCLUDE.**<br><br>- Data from the 1980–89 Monitoring the Future (MTF) surveys

Third path: Secondary societal impact without considering a bridging variable

[37]	First author, year, country	General aim	Study design	The policy measure(s) or intervention(s) at hand	Measured policy effects on MLDA	Reflection on policy and other occurrences by authors	Comments
	Wagenaar, 1981, USA.	The purpose of this investigation was to determine whether the raised drinking age resulted in a significant decrease in alcohol-related motor vehicle crashes among young drivers, that is, whether it reversed the effect of the lowered drinking age.	- Nonequivalent multiple time series design was used to assess the effects of the increase in drinking age.<br>- The dependent variables were monthly time series *based on a 20% random sample* of all reported motor vehicle crashes in the state of Michigan from 1972 through 1979. The condition of the driver reported by police as 'had been drinking' or 'had not been drinking' was used as an indicator for alcohol use.<br>- Box-Jenkins time-series analysis methods were used (using Auto-Regressive Integrated Moving Average; ARIMA).	- Raising the MLDA in Michigan in 1978 from 18 to 21.<br>- *Reduced national maximum speed limit was added to the analyses to control for extraneous sources of variance.*	- The first main finding is that nonalcohol-related (police-reported) motor vehicle crash involvement is down slightly for all age groups in 1979.<br>- The second main finding is that alcohol-related crash involvement, as measured both by the 'had-been-drinking' and three-factor surrogate indicators, was up slightly in 1979 for all drivers aged twenty-one to twenty-four and twenty-five to forty-five.<br>- The third principal finding is that the frequency of police-reported 'had-been-drinking' crash involvement among drivers aged eighteen to twenty is 30.7% lower in 1979 than one would have expected, had there been no change in the drinking age; the frequency of three-factor surrogate crash involvement is down 17.7% (sig.).	- The patterns evident in the raw time series, along with the time-series modeling estimation results, provide unmistakable support that raising the legal drinking age causes a reduction in alcohol-related crash involvement among young drivers.<br>- These results are even more surprising when the low level of enforcement of the raised drinking age is considered.<br>- Lowering the drinking age does not has a greater effect on crash involvement among youth than a subsequent return to a higher drinking age.<br>- Determination of the MLDA involves numerous considerations in addition to the public health consequences of alternative policies, such as the individual freedom of young people, long-term effects of alternative drinking ages and potential deleterious side effects of a high drinking age.	**INCLUDE.**<br><br>-

[38]	**First author, year, country**	**General aim**	**Study design**	**The policy measure(s) or intervention(s) at hand**	**Measured policy effects on MLDA**	**Reflection on policy and other occurrences by authors**	**Comments**
	Wagenaar, 1983, USA.	The purpose of this study was to assess the effect of raising the drinking age in Maine on fatal, injury and property damage crash involvement among young drivers.	- Monthly frequencies of motor vehicle crashes among drivers aged 18-45 in the states of Maine and Pennsylvania (comparison state without legislative changes) from 1972 through 1979 were examined.<br>- Information was gathered from Maine accident report forms provided by police officers investigating crashes (whether the driver had been drinking was used as one indicator of alcohol-related crash involvement).<br>- Additional data on single-vehicle nighttime male crashes were used as a comparison for police reported data.<br>- A multiple time series design was used, controlling for the effects of long-term trends, seasonal cycles and other factors with Box-Jenkins time series models.	In 1977, the legal drinking age in Maine was raised from 18 to 20.	- Raising the legal drinking age in Maine resulted in significant reductions in youthful alcohol-related property damage crashes (measured both by police reports and comparison analyses, an estimated 16.8% and 21.5% fewer 18- to 19-year-old drivers were involved in alcohol-related property damage crashes after the drinking age was raised), but had no demonstrable effect on the incidence of alcohol-related injury and fatal crashes among young drivers.	A plausible assumption for not finding effect on the incidence of injury and fatal crashes could be that young drinking drivers involved in injury and fatal crashes are likely to be heavier drinkers than drinking drivers involved in property damage chases. One could then speculate that the findings of the present study indicate that the drinking-driving behavior of heavy drinkers is less affected by the raised drinking age than drinking driving behavior of moderate drinkers.	**INCLUDE.**<br><br>-

[39]	First author, year, country	General aim	Study design	The policy measure(s) or intervention(s) at hand	Measured policy effects on MLDA	Reflection on policy and other occurrences by authors	Comments
	Asch, 1987, USA.	This study examines determinants of a variety of traffic fatality rates at the state level for 1978 (because few MLDA changes had occurred during the previous three years), with particular attention to drinking age and drinking experience.	This paper reports on a cross-state examination of the role of MLDA in traffic fatalities, drinking experience effects are considered in the presence of other determinants of traffic fatalities in an analysis of such effects in a pure cross-section data base for a single year (1978).	The MLDA-situation in 50 states in the US measured in 1978 (introducing drinking age measures into conventional regression equations explaining traffic fatality rates).	Our findings suggest that the minimum legal drinking age is not a significant, or even a perceptible factor in the fatality experience of all drivers or of young drivers, inexperience in drinking offers a more likely candidate to explain the peculiar fatality risk of young drivers, although a precise definition of its role will require further attention.	- Perhaps, the legal drinking environments of states, including both levels of and changes in MLDA, are correlated with underlying determinants of traffic hazard in ways that were not controlled in this study.<br>- Policy alternatives, such as educational programs, increased traffic safety enforcement, and use of heavier penalties for certain violations, may deserve more emphasis in our efforts to curb the tragic toll of drunk driving.<br>- The current legal environment, under which consumption of alcohol is prohibited until attainment of an arbitrarily specified age and permitted from that moment on, may itself create heavy costs in terms of driving risk. Some observers have suggested the desirability of introducing people at relatively early ages to "responsible" patterns of alcohol consumption. Conceivably, it might even prove desirable to allow people to start drinking before they start driving, either by lowering the drinking age, raising the driving age, or both. Our findings do not speak directly to such suggestions, but the evidence certainly indicates clearly that our current policies toward alcohol consumption are not successful from a public safety perspective.	**INCLUDE.**<br><br>- All fatality data were obtained from the Fatal Accident Reporting (FARS) of the National Highway Traffic Safety Administration.

[66]	First author, year, country	General aim	Study design	The policy measure(s) or intervention(s) at hand	Measured policy effects on MLDA	Reflection on policy and other occurrences by authors	Comments
	Coate, 1987, USA.	This article presents the first set of estimates of the responsiveness of youth alcohol use and motor vehicle death rates to variations in the price of alcohol. In addition, it examines the sensitivity of these two outcome measures to changes in the legal drinking age.	- Two survey datasets are used (NHANES I and II) between May 1971 and June 1974, and between February 1976 and February 1980.<br>- In studying youth demand for alcoholic beverages, we focus on alcohol use by youths aged 16 through 21.<br>- Infrequent and frequent beer consumption was measured by asking for the frequency of drinking beer in a week (no more information is provided).<br>- Data (vehicle accidents) based on a time series of state cross sections for the period from 1975 through 1981.<br>- There age groups were focused on: youths aged 15 through 17, 18 through 20 and 21 through 24.<br>- For both aims, estimates were obtained using variants of multiple regression analysis (controlling for multiple variables, e.g., border-hopping, age, family income, vehicle miles traveled per licensed driver).	- The upward trend in state MLDA for the purchase and consumption of alcoholic beverages.<br>- Increased taxation of alcoholic beverages (mainly focusing on beer).	- Use of alcohol by youths declines when either the price of alcoholic beverages or the MLDA increases. Youth alcohol consumption is inversely related at a statistically significant level to beverage price and MLDA, for both infrequent and frequent/heavy drinkers.<br>- Analysis indicate a statistically significant decline in the motor vehicle accident mortality rate with the effects of a real beer tax for youths aged 15 through 17, 18 through 20 and 21 through 24.<br>- Changes in the MLDA also produced statistically significant inverse effects on accident mortality for youths aged 18 through 20 (affecting the mortality of only those young people who might have legally purchased alcoholic beverages at the lower MLDA).<br>- Positive and significant coefficients of the border variable were obtained for the 18-through-20-year-old cohort.	- If reductions in youth alcohol consumption and motor vehicle accident deaths are desired, the preceding figures suggest that both a uniform MLDA of 21 and an increase in the Federal excise tax rate on beer are effective policies to accomplish this goal.<br>- They also suggest that the tax policy may be more potent than the MLDA policy.<br>- An excise tax increase lowers the death rates of youths between the ages of 15 and 17 and between the ages of 21 and 24. These age groups do not receive the same benefits from a rise in the MLDA, and also reduce fatal crashes involving adult drivers. However, a tax hike may greatly stimulate the demand for illegally produced beer.<br>- Young drivers may respond to increasingly severe penalties for offenses only if the possibility of apprehension and conviction is not trivial. If substantial resources must be allocated to increasing the probability of arrest and conviction, a policy of increased excise taxes may be preferable to or complementary with a system of penalties.	**INCLUDE.**<br><br>- The first and second National Health and Nutrition Examination Surveys (NHANES I and NHANES II).<br>- Data on youth motor vehicle accidents mortality (FARS).

[69]	**First author, year, country**	**General aim**	**Study design**	**The policy measure(s) or intervention(s) at hand**	**Measured policy effects on MLDA**	**Reflection on policy and other occurrences by authors**	**Comments**
	Dee, 1999, USA.	This empirical study presents new evidence on the efficacy of state alcohol policies (i.e. excise taxes on beer and minimum legal drinking ages) in reducing the prevalence of teen drinking and its key related outcome, youth traffic fatalities.	- The evaluations presented here are based on data set that consists of pooled cross-sections from the 1977–92 Monitoring the Future (MTF) surveys of high school seniors.<br>- Consumption behavior was categorized into ‘‘drinkers’’ (any drink of alcohol in the last month), ‘‘moderate drinkers’’ (10 or more drinks of alcohol in the past month) and ‘‘heavy drinkers’’ (5 or more drinks in a row sometime in the last 2 weeks).<br>- The other key evidence linking state alcohol policies and teen drinking by evaluating empirical models of youth traffic fatalities are based on reduced-form models of youth traffic fatalities based on a relatively long panel (1977–92) of annual state-level data.	Excise taxes on beer and minimum legal drinking ages between 1977-92.	- Beer taxes have a relatively small and statistically insignificant impact on teen drinking. Also, the empirical models of traffic fatalities demonstrated that the conventional link between beer taxes and youth traffic fatalities is not a robust one.<br>- The results of these estimations indicate that the movement to higher minimum legal drinking ages substantially reduced teen drinking and led to large and statistically significant reductions in youth traffic fatalities (estimates suggest that the movement to higher MLDA reduced heavy teen drinking by at least 8% and traffic fatalities by at least 9%).	The success of higher minimum legal drinking ages in reducing the prevalence and social consequences of teen drinking suggests that current and future efforts to curb teen alcohol use should recognize the probable efficacy of policies that further increase the non-pecuniary cost to teens of acquiring alcohol.	**INCLUDE.**<br><br>- Data from Monitoring the Future (MTF) surveys.<br>- Data from Fatal Accident Reporting System (FARS).

[72]	First author, year, country	General aim	Study design	The policy measure(s) or intervention(s) at hand	Measured policy effects on MLDA	Reflection on policy and other occurrences by authors	Comments
	Kaestner, 2009, USA.	We examine whether adult alcohol consumption and traffic fatalities (adults with an average age of 35) are associated with the legal drinking environment when a person was between the ages of 18 and 20.	- The two outcomes we examine are alcohol use and traffic fatalities.<br>- Data on alcohol use comes from the BRFSS, from the years 1995 to 2005. We limited the sample to individuals from those survey years born between 1960 and 1975 because these individuals were in their late teens in the early to late 80s when most states raised the MLDA to 21.<br>- The BRFSS contains self-reported information about alcohol use in the past month. Several measures were constructed: 1) the proportion of days drank alcohol in past month, 2) number of drinks in past month, 3) whether binge drank in past months (5 or more drinks at one time), and 4) the proportion of days binge drank in past month.<br>- Data on fatal traffic accidents were taken from FARS, from the years 1995 to 2006, focusing on nighttime fatalities.<br>- Regression analyses were conducted.	- Changes in MLDA in the U.S.<br>- Federal and state beer taxes.	- Men who grew up in an environment that allowed them to drink between the ages of 18 and 20 reported drinking a greater number of drinks in the past month (sig.) and more episodes of binge drinking in the past month (sig.) than men who grew up in an environment that prohibited them from drinking during late adolescence. Magnitudes of the associations were non-trivial suggesting differences of 20 to 30 percent in these measures of alcohol use, and even larger for low-educated males.<br>- Again, for males, traffic fatalities (per 100,000) of those who were always able to legally drink between ages 18 and 20 are between 2.6 (9 percent) and 3.1 (11 percent) higher than for those who were never able to legally drink between ages 18 and 20 (sig.).<br>- A similar result is obtained in the case of evening accidents (also for males); those always able to legally drink had approximately 10 percent more accidents than those never able to legally drink (sig.).<br>- There were no statistically significant or practically important associations between the legal drinking environment when young and adult female alcohol consumption and driving fatalities.	To summarize, we found that a minimum legal drinking age of 21, versus a minimum age of 18, is associated with a 20 to 30 percent reduction in adult male alcohol use and a ten percent reduction in fatal traffic accidents with adult male drivers. These estimates support arguments made by those in favor of keeping the minimum legal drinking age at 21 that such laws have long term, beneficial consequences. Importantly, the evidence we present is a direct assessment of that argument and does not rely on hypotheses of long-term effects of such laws derived from neurobiological studies of how alcohol use affects adolescent brain development.	**INCLUDE.**<br><br>- Behavioral Risk Factor Surveillance System (BRFSS).<br>- Data on fatal traffic accidents were taken from the Fatality Analysis Reporting System (FARS).

[74]	First author, year, country	General aim	Study design	The policy measure(s) or intervention(s) at hand	Measured policy effects on MLDA	Reflection on policy and other occurrences by authors	Comments
	Zhang, 2011, USA.	This study examined: (1) the relationship between alcohol-control environment during young women's early childbearing years, indicated by the MLDA laws at their 14th birthday, and infant health indicators; (2) whether changes in birth composition, drinking behaviors, or formation of drinking habits contributed to change in infant health; and (3) whether racial differences relative to the above issues existed.	- State- and year-fixed-effects models are used to analyze the relationship between MLDA, drinking behaviors, and birth outcomes.<br>- Regarding drinking behavior, two variables were constructed accordingly: drinking prevalence and the incidence of at least five alcoholic beverages per occasion (i.e., binge drinking) during the previous month.<br>- We studied the effects of different MLDA (age 18, 19, 20, or 21 years) when potential mothers were 14 years old by merging two population-based datasets between 1985 and 2002.	- State-specific variations of MLDA (18, 19, 20 or 21) when mothers were 14 years old.<br>- Real beer taxes (federal plus state) were added to the analyses, since beer taxes changed between 1970 and 1989.	- A MLDA of 18 years old (when potential mothers were 14 years old) increased the prevalence of low birth weight, low Apgar scores, and premature births (all sig.).<br>- Effects were stronger among children born to black women compared with white women (sig.).<br>- An MLDA at age 21 years decreased the probability of alcohol drinking among black women by 2–8% points (sig.).<br>- Those who lived in a state where the MLDA was age-18, were 2.2 percentage points (sig.) more likely to binge than those who lived in states with an older MLDA. Exposure to age-20 or -21 MLDA reduced the binge drinking by about 6.6 percentage points (sig.).	- These results indicate that the MLDA changed drinking behaviors among white and black women in a different way. For black women, a movement away from age-18 as the drinking age decreased overall drinking probability to a relatively modest degree and decreased binge drinking substantially more. Apparently, an increase in the legal drinking age modified the formation of habitual drinking by effectively reducing the availability of alcoholic beverages. For white women, an older MLDA modestly reduced overall drinking, but not binge drinking. This suggested that it was moderate white drinkers rather than heavy ones who were sensitive to changes in MLDA.<br>- Not all policies, such as expansion of Medicaid for pregnant women, were accounted for in this study. In addition, some other factors like insurance status, utilization and frequency of pre-natal care may influence infant health, but were not included in the analysis. Hence, the omitted variable bias may still exist.	**INCLUDE.**<br><br>- The Vital Statistics Natality Detailed Files from 1970 to 1992.<br>- The Behavioral Risk Factor Surveillance System from 1985 to 2002.<br>- The Behavioral Risk Factor Surveillance System (BRFSS) from 1985 to 2002 to investigate women's health behaviors.

[75]	First author, year, country	General aim	Study design	The policy measure(s) or intervention(s) at hand	Measured policy effects on MLDA	Reflection on policy and other occurrences by authors	Comments
	Lillis, 1987, USA.	Assessing the impact of increasing the legal drinking age on drinking and driving behavior.	- A multiple-indicator, pre-comparison and post-comparison design was used.<br>- Changes in indicators for 18-year-olds were compared with changes for other age groups.<br>- Three independent data sources related to drinking and driving were utilized: 1) the accident records submitted by police agencies to the Department of Motor Vehicles (data from two periods was used; 12/4/81 to 12/3/82 and 12/4/82 to 12/3/83), 2) arrest records maintained by the New York State Police, and 3) self-reported drinking-and-driving data (measuring 'driving after feeling the effects of alcohol' at least once in the 28 days prior to the survey), and questions regarding the purchase behavior of specific beverages (i.e., beer, wine or liquor) in the preceding 28 days at specific locations (i.e., bars of stores)) culled from the Youth Alcohol Survey (using telephone interviews comprising 2000 16- to 20-year-old New Yorkers conducted in 1982 (before the increase) and 1983 (after the increase)).	The legal minimum purchase age for alcohol beverages in New York State that increased from 18 to 19 years old in 1982.	- Analyses of three independent indicators of drinking and driving provide strong support for the hypothesis that the increase in the legal purchase age in New York State from 18 to 19 years resulted in decreases in drinking-and-driving behavior by 18-year-olds.<br>- Changes in the rates of drinking-and-driving indicators showed a 21% decrease in fatal and injury-causing crashes and a 39% decrease in fatal crashes for 18-year-olds (sig.).<br>- Non-crash DWI arrests of 18-year-old drivers decreased 35% (sig.).<br>- A 46% decrease in self-reported drinking and driving from the survey were found for 18-year-olds (sig.) following the increase in the purchase age to 19.<br>- For all measures, the rate of decrease for 18-year-olds was significantly greater than the rate of decrease for drivers 21 years old and older.<br>- After the increase, the rate of beer purchasing by 18-year-olds was significantly smaller than the rate for 19- or 20-year-olds (33% vs. 51% and 47%, respectively), and decreased significantly for 18-year-olds following the increase (from 52% to 33%, respectively).	- The findings support the theory that the 19-year-old purchase age in New York has had a major immediate impact on 18-year-olds in the state.<br>- Opponents of purchase-age increases have argued that any changes in drinking-driving crash rates are due to the STOP-DWI legislation and programs, and not to purchase age policies. However, none of the laws and few programs have been aimed specifically at young drivers (e.g., 18-year-olds). Efforts of STOP-DWI cannot explain the differences between the rate of change for 18-year-olds.	INCLUDE.<br><br>- The accident records submitted by police agencies to the Department of Motor Vehicles.<br>- Arrest records maintained by the New York State Police.<br>- The Youth Alcohol Survey.

[82]	First author, year, country	General aim	Study design	The policy measure(s) or intervention(s) at hand	Measured policy effects on MLDA	Reflection on policy and other occurrences by authors	Comments
	Decker, 1988, USA.	- It is essential to know whether raising the MLDA to 21 can materially reduce the number of deaths among persons aged 15 through 24 years caused by motor vehicle crashes (MVCs).	- Analyzed the Tennessee MVC data (FARS) for 1980 through 1986. Additionally, a count of licensed drivers by county, population and deaths counts by county, an estimate of total miles driven in Tennessee, an estimate of the total and nighttime miles driven and estimates of the prevalence of self-reported DUI were used.<br>- Three age groups were considered; those aged 15 through 18 years, 19 through 20 years and those aged 21 through 24 years.<br>- Focus was on single-vehicle nighttime (SVN) fatal crashes to obtain a sensitive measure for the extent of alcohol involvement in MVCs.<br>- Rates within a given age stratums were compared by using the incidence density ration method, to compare rates among age groups, two-tailed t-tests were performed.	- In 1984, Tennessee raised its MLDA for possession or purchase of alcohol from 19 to 21 years (the age-law).<br>- In 1982, Tennessee adopted legislation that markedly increased the likelihood of imposition and the severity of penalties for conviction for DUI (the penalty-law).	*(only focusing on effects subsequent to the age law)*<br>- There was a significant and persistent decline (38%) in the *SVN fatality rate* among drivers aged 19 through 20 years following implementation of the age-law (no border effects were found).<br>- Among persons aged 15 through 18 years, in 1985, the year following the introduction of the age-law, the *overall* MVC death rate dropped 24% (sig.).<br>- Similarly, in 1985, the year following introduction of the age law, the *overall* MVC death rate dropped 24% (sig.) among persons aged 19 through 20 years.<br>- During 1980 through 1982, the *crude death rate* for persons aged 15 through 18 years declined 13% for 1883 through 1986 (sig.).<br>- *The mean BAC* in 1985 for youth 15 through 18 years was 48% lower than all other years in the study period (sig.). After implementation of the age-law, the mean BAC for 19 through 20-year-olds showed a 35% reduction (sig.) and 20% (sig.) for drivers aged 21 through 24 years.	- The implementation of a law denying alcohol to persons aged 19 through 20 years caused a sudden and dramatic decline in drunk driving among that age group, an effect still present at the end of the study period.<br>- Stiffened penalties or increased enforcement directed against DUI has been associated with declines in DUI fatality rates, however, beneficial effects began before the legal changes, parallel with increasing publicity and social disapproval stimulating those legal changes, and beneficial effects almost invariably disappeared within a few years.<br>- Our data suggest that publicity and other social influences may have played a particularly important role in producing the prolonged reduction in alcohol related MVC mortality seen in the 15-through 18-year-old age group.<br>- Our data indicate that laws raising the MLDA to 21 can be highly effective in reducing alcohol-related MVCs among drivers aged 19 through 20 years, a group apparently quite resistant to the effects of increased DUI penalties end anti-DUI publicity.<br>- We cannot clearly apportion the responsibility for the benefits among influences link DUI laws or a new MLDA, but each appears contributory.	**INCLUDE.**<br><br>- Data on fatal traffic accidents were taken from the Fatality Analysis Reporting System (FARS).

[83]	**First author, year, country**	**General aim**	**Study design**	**The policy measure(s) or intervention(s) at hand**	**Measured policy effects on MLDA**	**Reflection on policy and other occurrences by authors**	**Comments**
	Figlio, 1995, USA.	Monthly Wisconsin time-series data from 1976 to 1993 were used to estimate the effects of increased minimum drinking ages on alcohol-related crashes involving teenagers.	- This study analyzes 18-year time series data with monthly observations of alcohol-related crashes in the state of Wisconsin, stratified by age, from 1976 to 1993.<br>- The long time-series were used to utilize Box-Jenkins time series techniques to gauge the effects of the introduction of Wisconsin's minimum drinking age laws.<br>- The over-21 age group whose members could drink legally over the entire time period was used as a control group. - A two-way random effects model was used to determine the extent of border hopping as a result of interstate drinking age differences.	Wisconsin, the state in question in this study, raised its drinking age to 19 in 1984 and to 21 in 1986. Prior to 1986, Wisconsin had a lower drinking age than did its neighbors, suggesting that border hopping may have occurred.	- Raising the drinking age has resulted in substantially lower alcohol-related crash rates involving teenagers (sig.).<br>- Crashes increased in years in which Wisconsin's drinking age was lower than those of its neighbors, suggesting that "border hopping" resulted from interjurisdictional policy differences (sig.). Counties with moderate border traffic and interstate drinking age differentials have substantially more alcohol-related crashes than other counties, all else constant.	- A uniform, 21-year drinking age does, as critics argue, reduce state-level autonomy in determining alcohol policy.<br>- In addition, to the extent that a large percentage of teenagers do not drink and drive, increasing the drinking age to 21 does present a dilemma with respect to fairness. Forbidding all teenagers from drinking may indeed penalize many for the actions of a few.	**INCLUDE.**<br><br>-

[84]	First author, year, country	General aim	Study design	The policy measure(s) or intervention(s) at hand	Measured policy effects on MLDA	Reflection on policy and other occurrences by authors	Comments
	Lovenheim, 2010, USA.	An important but unexamined policy parameter is the degree to which cross-state differences in MLDAs induce teenage drunk driving. Most of the research addresses the effect of raising (in a majority of states) the drinking age to 21, while little attention has been paid to the equalization of the drinking ages. This paper adds to the literature by examining the effect of MLDA evasion across states with different alcohol restrictions from 1977 to 2002.	We use Geographic Information System (GIS) software to match with each U.S. county the closest locality in which an 18, 19, or 20-year-old legally can purchase alcohol and measure the population-weighted average distance from the county to that locality. Then, using data from FARS covering 1977–2002, which contains information on every fatal accident in the United States, we show that accidents involving only older drivers vary systematically with MLDA changes and with the distance to lower-MLDA borders. This variation suggests a difference-in-difference methodology is necessary to control for spurious fatal accident variation that is correlated with the timing of MLDA increases. We then estimate such a difference-in-difference model, which identifies how the likelihood that an 18, 19 or 20-year-old driver is involved in a fatal accident relative to older drivers varies with MLDA law changes and distance to lower-MLDA borders (using a longitudinal design).	Changes in MLDA between 1977 to 1988, and the equalization from 1988 onwards.<br><br>Controlling for:<br>- 0.08 per se laws.<br>- 0.02 zero tolerance laws.<br>- primary/secondary seatbelt law.<br>- BAC Law.<br>- Beer Tax.	- The effect of restricting alcohol by raising the MLDA locally increases the likelihood that an 18 or 19-year-old (but not a 20-year- old) driver is involved in a fatal accident (relative to all drivers over 25 years old) caused by the proximity of lower MLDAs.<br>- For counties more than 25 miles from a lower MLDA border, raising the drinking age within a state has a negative and statistically significant effect on the likelihood that a teenage driver is involved in a fatal accident. Thus, for countries more than 25 miles from a lower MLDA border, the results were consistent with previous literature that MLDA restrictions are effective in reducing accident fatalities.<br>- The effect of changes in the MLDA is quite heterogeneous across states, depending on the fraction of a state's population that need not travel far to reach a state with a lower MLDA.	Our results suggest that, by ignoring MLDA evasion, previous studies have underestimated the total effect of MLDA increases on teenage drunk driving. Unequal policies across unmonitored borders can induce the very behaviors the restrictions are meant to eliminate.	**INCLUDE.**<br><br>- Data from the Fatal Accident Reporting System (FARS) were used.

[85]	**First author, year, country**	**General aim**	**Study design**	**The policy measure(s) or intervention(s) at hand**	**Measured policy effects on MLDA**	**Reflection on policy and other occurrences by authors**	**Comments**
	MacKinnon, 1986, USA.	To evaluate the effect of raising the minimum drinking age on driver fatalities in Illinois, Massachusetts, and Michigan.	Time series analysis was used to obtain statistical tests of the impact of raising the drinking age on monthly driver fatalities in Illinois, Michigan and Massachusetts. An interrupted control series design permitted comparison between younger drivers (21 or less years) and older drivers (25 and older) within states where the minimum drinking age was raised. Control states (Missouri, Connecticut and Ohio) were selected to permit a comparison between driver fatalities of the young age group (21 or less) in states with the law change and young drivers in states without the law change.	Changes in MLDA in Illinois, Massachusetts and Michigan from 1975 to 1981.	- Significant immediate reduction in fatalities among 21 and younger drivers in Illinois and Michigan were observed after these states raised their MLDA.<br>- No significant reductions in any control series were observed.<br>- A linear decrease in young driver fatalities was observed after the drinking age was raised in Massachusetts.<br>- There was also a significant linear decrease in young driver fatalities in the Connecticut control series.	- It appears implausible that the change in driver fatalities is due to changes in road or weather conditions, or driving habits in the law-change states, since older drivers in those states did not experience a significant shift in driver fatalities.	**INCLUDE.**<br><br>- Data from the Fatal Accident Reporting System (FARS) were used.

[86]	First author, year, country	General aim	Study design	The policy measure(s) or intervention(s) at hand	Measured policy effects on MLDA	Reflection on policy and other occurrences by authors	Comments
	Males, 1986, USA.	- In this essay, the merits and probable long-term effects of the 1984 federal law raising the MLPA to 21 are discussed and examined for the entire age group they affect (under 21 drivers in general).<br>- The aim was to repeat the analysis of the Insurance Institute Study (IIHS) using mostly comparable, but slightly different variables and techniques to investigate the robustness of these findings; If MLPA increases are really the cause of the decline in nighttime fatal crashes among drivers in the affected age group as reported in the IIHS study, then that decline should show up consistently even when a slightly different data base is used.<br>- Another aim was to test the hypothesis that the first year of legal drinking is associated with a disproportionately high number of fatal crashes.	To analyze the effects of MLPA increases over time on fatal car accidents, several types of comparisons were made between overall and nighttime fatal crash data from 1975 through 1983 from different age groups (using drivers aged 21-24 as a control group) between 14 states which raised their MLPA during 1976-81 and matched control states (in which no MLPA raise took place in this period).	Data from 1975 through 1983 are used to examine states that raised their MLPAs.	- Efforts by the author to duplicate the IIHS findings found no savings in lives attributable to raised drinking ages.<br>- Raising MLPAs may not reduce fatalities from drunken driving.<br>- A state MLPA is associated with an increase averaging 5 percent in fatal crashes involving drivers in their first year after they reach the purchase age, suggesting that the first year of legal drinking is associated with a disproportionately higher number of fatal crashes.<br>- When a state raises its MLPA, there is a corresponding, and unequal, shift in increased fatal crashes from a lower to a higher age group. The net decrease in fatal crashes among drivers of the age corresponding to the old MLPA averages 6 percent. The net increase among drivers whose age corresponds to the new MLPA level averages 15 percent.<br>- States that did not raise their MLPAs during the 1976-81 period experienced a slightly larger decrease in fatal crashes among drivers under twenty-one years of age than did those states that did raise their MLPAs. The difference is not large enough to be called significant, but it does suggest that raised drinking ages are not associated with any net reductions in fatal crashes by young drivers.	- The conclusions from Table 2 of this study are that MLPA increases have no effect on young drivers and that any decrease in nighttime fatal crashes in the MLPA-increase states was due to a general trend that affected drivers in the just-older control group as well as those in the affected age group.<br>- The IIHS study and the NTSB projections both drastically overestimated the life-saving potential of a national MLPA of twenty-one. The IIHS findings have been extended beyond their proper limits. The conclusions consequently reached have been flawed because the IIHS failed to examine raised MLPA effects using a long enough period of time and a stable enough data base.<br>- There is a national interest involved in preventing border-hopping by youths driving from states with higher MLPAs to states with lower MLPAs. This interest could be better served, and the negative consequences of raised MLPAs avoided, by a national MLPA of eighteen. There is no point in subjecting millions of eighteen-, nineteen-, and twenty-year-old's to arrest, exclusion from entertainment, and other social restrictions for engaging in the otherwise legal activity of buying and drinking alcoholic beverages in public unless large benefits to them and society can be firmly demonstrated.<br>- A graduated drinking age appears worth designing and implementing, not only on scientific grounds, but because it carries the prospect of reducing or eliminating the higher death rates of youths in their first year of legal drinking.	**INCLUDE.**<br><br>- Data from the Fatal Accident Reporting System (FARS) were used.<br>- Findings from the National Transportation Safety Board (NTSB) are projected.

[87]	First author, year, country	General aim	Study design	The policy measure(s) or intervention(s) at hand	Measured policy effects on MLDA	Reflection on policy and other occurrences by authors	Comments
	Ponicki, 2007, USA.	The current study tests explicitly for interdependence between the impacts of MLDA and beer taxes in models of traffic fatalities. It is anticipated that the impacts of MLDA will diminish as one looks at age groups more distant to the 18- to 20-year olds more directly affected by these laws. The current study thus compares the interacted MLDA and tax effects across various youth and adult age groups.	The interdependence between the impacts of MLDA and taxes is investigated using a panel of 48 US states over the period 1975 to 2001. This is accomplished by introducing a multiplicative interaction term between MLDA and beer tax rates. All age-group–specific regression models control for numerous other variables previously shown to affect vehicle fatalities, as well as fixed effects to account for unexplained cross-sectional and time-series variation. The dependent variable in each model is the number of age-specific vehicle-occupant fatalities occurring in a state divided by the number (in thousands) of state residents of the relevant age group.	The current analyses concentrate on 2 forms of limitations on availability that have been shown to affect youth traffic fatalities: minimum legal drinking age (MLDA) laws and beer taxes.	- The present analyses indicate that traffic fatalities among those aged 18 to 20 can be effectively reduced by elevating the MLDA and by increasing beer tax rates (i.e., fatalities decline with higher MLDA and beer tax rates have a negative impact on youth fatalities) and MLDA has the largest and most-significant impact on fatalities aged 18 to 20.<br>- However, the direct negative impact of the beer tax variable on youth fatalities drops by half and is only marginally significant when the interaction term is introduced.<br>- The variable measuring the interaction between MLDA and beer taxation has a significant impact in the anticipated negative direction, indicating that each limitation on alcohol availability has a smaller impact on fatalities as the other form of availability is more restricted.<br>- As expected, a given change in MLDA causes a larger proportional change in fatalities when beer taxes are low than when they are high.	- These findings suggest that a community's expected benefit from a proposed limitation on alcohol availability depends on its current regulatory environment. Specifically, communities with relatively strong existing policies might expect smaller impacts than suggested by prior research, while places with weak current regulations might expect larger benefits from the same policy initiative.<br>- Higher MLDA are responsible for a smaller impact of beer taxes on youth drinking and fatalities, and strengthening drunken driving policies may have reduced the marginal impact of taxes and prices, which could explain the failure of many recent US studies to find significant tax effects on traffic crashes.<br>- The current study's findings suggest that the effectiveness of any given policy will vary over time and place, and more specifically will be more or less effective based on how much other regulation a community has (i.e., the current analyses indicate that local characteristics must be taken into account when predicting the impact of a proposed policy change).	**INCLUDE.**<br><br>- Data is derived from the annual micro-data sets of the Fatal Accident Reporting System (FARS).

[88]	First author, year, country	General aim	Study design	The policy measure(s) or intervention(s) at hand	Measured policy effects on MLDA	Reflection on policy and other occurrences by authors	Comments
	Wagenaar, 1982, USA.	The evaluation reported below examined all reported crash-involved drivers in Michigan from January 1972 through December 1979.	- The nonequivalent multiple time- series design was used to evaluate the effects of Michigan's increase in legal drinking age. The postulated causal relationship was between changing the legal drinking age and traffic crashes.<br>- The broadest of the three levels of comparisons was an analysis of two different states, one that had raised the drinking age in the late 1970s and one with no such legal change (New York, with a drinking age at 18 throughout the study period).<br>- Box-Jenkins time series analysis was used, along with the Auto Regressive Integrated Moving Average (ARIMA) modeling strategy.	- Raising the MLDA in Michigan in 1978 from 18 to 21.	- Significant 11 to 28% reductions in alcohol-related crash involvements are shown to be attributable to the raised drinking age in Michigan.<br>- No unequivocal effects of the raised drinking age on underage (16-17) drivers were found the first year after the change.<br>- The conclusion that these reductions are due to the drinking age is strengthened by finding no significant changes in alcohol-related or non-alcohol-related crashes among drivers ages 21-23 or 24-45.<br>- The analyses were repeated for the state of New York, which did not change its legal minimum drinking age during the 1970s. There were no significant decreases in alcohol-related crash involvement among young drivers at the time Michigan raised the drinking age.<br>- The substantial decrease in alcohol-related crash involvement among 18-20-year-old drivers from 1978 to 1979 (long-term) is again clearly evident.	- Alcohol-related crash involvement among young drivers clearly decreased after the legal minimum drinking age was raised in Michigan.<br>- It is not clear whether the beneficial effects of higher drinking ages are due to: (1) reduced quantity of alcohol consumed by youth on each drinking occasion; (2) fewer drinking occasions; (3) changes in drinking locations so that less driving while impaired is required; (4) more cautious driving while impaired; or (5) some combination of these effects.<br>- The reductions in alcohol-related crash involvement for 1980 and 1981, which occurred for drivers of all ages (data not shown), were probably a result of the severe economic recession in Michigan, as well as other highway safety programs during those years.<br>- The legal minimum drinking age substantially reduces alcohol-related crash involvement among young drivers; that it does not eliminate this serious problem is no reason to reject minimum drinking age as one component of a broader prevention effort.	**INCLUDE.**<br><br>- Same data and U.S. state (Michigan) as study 68, only now using a full sample (and not a 20% random sample) and more investigated years.

[89]	First author, year, country	General aim	Study design	The policy measure(s) or intervention(s) at hand	Measured policy effects on MLDA	Reflection on policy and other occurrences by authors	Comments
	Wagenaar, 1986, USA.	Results of a 6-year follow-up of previous research evaluating the effects of Michigan's 1978 increase in the legal drinking age from 18 to 21 are reported.	- The current study examined 6 years of post-law traffic crash data in the state of Michigan from 1976 through 1984, using Box-Jenkins intervention analysis methods to assess the long-term effects of the raised drinking age.<br>- Auto-Regressive Integrated Moving Average (ARIMA) models were developed using iterative identification, estimation and diagnosis strategies.	- Raising the MLDA in Michigan in 1978 from 18 to 21.	- Over the 6-year follow-up period, the rate of involvement in injury-producing single-vehicle nighttime crashes among drivers aged 18-20 was 16% lower than the level expected, had the drinking age law not changed (sig.).<br>- Police-reported drinking driver crash involvement was down 19% (sig.).<br>- The long-term effects of Michigan's increase in legal drinking age are not significantly different from the short-term effects identified in earlier research.	- In contrast to many alcohol-impaired driving countermeasures, the raised legal drinking age appears to have a long-term effect in reducing motor vehicle crash involvement among young drivers.<br>- Despite safety benefits of a higher drinking age, some argue in favor of a lower drinking age based on equity, difficulty in enforcement and other considerations not related to health and safety. Given such low enforcement levels, some argue that the higher drinking age may foster disrespect for the law among youth, and that the lack of resolve to enforce this law indicates that other strategies may be more effective in reducing crashes among drivers of all ages. Although all of these issues deserve attention in the policy debates surrounding the drinking age, they should not obscure the research evidence that an increase in drinking age from 18 to 20 or 21 lowers the incidence of traffic crashes among young alcohol-impaired drivers.<br>- The legal age for drinking should not be viewed as an isolated policy. Rather, it is one example of an approach to the prevention of alcohol-related problems that focuses on restricting the availability and distribution of alcoholic beverages.<br>- Public policies on other dimensions of alcohol availability not limited to one specific age group (e.g., retail price of alcohol; design, location, number, and density of alcohol outlets; and selling, serving, and marketing practices) should be examined for their utility in the prevention of alcohol-related health and social problems.	**INCLUDE.**<br><br>- Same data and U.S. state (Michigan) as study 68 and 74, only now investigating long-term effects.

[90]	First author, year, country	General aim	Study design	The policy measure(s) or intervention(s) at hand	Measured policy effects on MLDA	Reflection on policy and other occurrences by authors	Comments
	Wagenaar, 1986, USA.	- The goal of the current study was to measure the effects of a 1-year increase in the legal age for purchase of alcoholic beverages on youth crash involvement in Texas.<br>- The short- and intermediate-term effects of that law were assessed, using data on motor vehicle crashes occurring between 1978 and 1984.	- Using an interrupted time-series design, rates of single-vehicle-nighttime (SVN) and non-SVN crashes per 100,000 licensed drivers from 1978 through 1984 were examined for three levels of crash severity (serious injury, minor injury, property damage only) and four age groups (16-17, 18, 19-20, 21 and over).<br>- Effects were examined using Box-Jenkins interrupted time-series intervention analyses, conducting iterative Auto-Regressive Integrated Moving Average (ARIMA) model identification, estimation and evaluation techniques.	Texas raised its legal age for drinking from 18 to 19 for all types of alcoholic beverages effective 1981.	- Results revealed significant reductions in SVN crashes for the 18-year-old target population across all levels of crash severity: serious injury, down 10.8%; minor injury, down 14.3%; and property damage only, down 12.8%.<br>- In comparison, no significant changes in SVN crashes among drivers age 21 and over were found.<br>- When the effects of macroeconomic conditions on crash rates were controlled statistically, no change in the estimated effect of the legal age law was seen.	- It is clear that the 1-year increase in legal age in Texas had a significant effect on youth crash involvement.<br>- Results of the current study support the contention that efforts to prevent alcohol-related motor vehicle crashes among youth should focus on changes in broader social and policy environments, not on attempts to modify the behavior of individual drinking drivers.<br>- Although not directly examined in this paper, an alternative policy that may have a large effect on youth drinking/driving is in- creasing the price of alcohol. A price policy would apply to all drinkers and not be limited to a single age group.<br>- The advantages of a price policy do not eliminate the benefits of the minimum legal drinking age. Multiple prevention avenues are required to achieve substantial reductions in the major public health problems associated with the use of alcoholic beverages.	**INCLUDE.**<br><br>- The study used data on fatalities in crashes with police-reported alcohol involvement supplied by the National Highway Traffic Safety Administration's (NHTSA) Fatal Accident Reporting System (FARS).

[91]	First author, year, country	General aim	Study design	The policy measure(s) or intervention(s) at hand	Measured policy effects on MLDA	Reflection on policy and other occurrences by authors	Comments
	Asch, 1990, USA.	This study tests the hypotheses that 'new drinking' per se creates an increased driving fatality risk, a risk that is at least partially independent of the legal drinking age.	A covariance model is employed to examine the effects of legal drinking age and drinking experience of traffic fatality rates. The model pools data from 47 states for the period 1975-1984, estimating separate equations for each age category 18 through 21.	The changes in MLDA in 47 states in the US between 1975 and 1984.	Our results are consistent with the hypothesis that drinking experience, or the lack thereof, plays an important role in traffic fatalities. Once inexperience is considered, the safety effect of drinking age laws is unclear.	- Raising the drinking age seems primarily to postpone fatalities, although the possibility of some net lifesaving effect cannot be ruled out.<br>- Further attention should be directed to the role of driving experience and to the analysis of more direct measures of alcohol-involved accidents and fatalities.<br>- Public policy might focus more profitably on educational efforts or taxes to discourage alcohol consumption more generally and traffic enforcement programs aimed specifically at new drivers of new drinking drivers.	**INCLUDE.**<br><br>Data were obtained from the Fatal Accident Reporting System (FARS) of the National Highway Traffic Safety Administration (NHTSA).

[92]	**First author, year, country**	**General aim**	**Study design**	**The policy measure(s) or intervention(s) at hand**	**Measured policy effects on MLDA**	**Reflection on policy and other occurrences by authors**	**Comments**
	Dumochel, 1987, USA.	The present study was undertaken to assess longer-term effects of raising the alcohol purchase age. It has the advantage of including the experience of additional states that have enacted such legislation.	We compared changes in fatal crash involvement among affected drivers before and after the age changes with the experience of drivers not affected by the age change in those same states. The fatal crash involvements among affected drivers were also compared with those of same-age and other-age drivers in states that did not change their laws in the years 1975-84. The statistical analysis used produces regression coefficients that estimate the proportional reduction in driver fatal crash involvement rates associated with the prohibition of alcohol from drivers in particular state-age-year combinations.	The data available for this study include the years 1975-84, and it was possible to study twenty-six states that changed their laws during this period.	- Based on the 87,153 nighttime driver fatal crash involvements that occurred during 1975-84, raising the minimum legal alcohol purchase age was estimated to produce a 13 percent reduction in nighttime driver fatal crash involvements (sig.).<br>- In states with several years of experience with the raised purchase age law, no significant differences in the effects of the age change were observed after the first years of the change.<br>- The effect of the hazardousness of the first year of legal alcohol purchase was negligible and after a rerun, nonsignificant.	One possible interpretation of these results is that the law changes had the effect of reducing fatality rates not only for drivers whose legal ability to purchase alcohol is affected by the laws but for younger and older drivers (including "beginning" drinkers) as well.	**INCLUDE.**<br><br>- Fatal Accident Reporting System (FARS) data is used, based on drivers aged sixteen through twenty-four who were in crashes in which someone was killed during 1975-84 in 48 states in the US.<br>- Population estimates for each state were obtained from the U.S. Bureau of the Census.

[93]	First author, year, country	General aim	Study design	The policy measure(s) or intervention(s) at hand	Measured policy effects on MLDA	Reflection on policy and other occurrences by authors	Comments
	Durant, 1993, USA.	- The purposes of this study are twofold: (1) to demonstrate how multiple interrupted time series with nonequivalent control variables can afford a more rigorous and statistically sophisticated approach to the study of policy design; (2) to assess the promise and pitfalls of the policy tools approach as a vehicle for developing mid-range theories of social regulatory policy.<br>- To these ends, we use traffic safety reform as a policy window for examining over time the independent comparative and conjoint impacts from 1975 through 1987 of three traffic safety reforms in Michigan.	We used interrupted time series analysis to estimate the effects of the various social regulatory tools. Because of seasonality and autocorrelation problems in each series, we employed Autoregressive Integrated Moving Average (ARIMA) analysis.	- Elevating the minimum-level drinking age (MLDA) from 18 to 21 in 1978.<br>- Increasing the certainty of penalty for driving while impaired by alcohol in 1983.<br>- Passing a mandatory seat belt law (MSBL) effective in 1985.	- To assess the actual impact of the three regulatory tools, controlling simultaneously for the effects of the others (plus the two control variables), we computed the estimates of intervention. Analysis revealed that neither the seat belt law nor miles driven exhibited statistically significant impacts on fatalities in any time series.<br>- After identifying the appropriate model and estimating it *for all drivers*, the impact of the MLDA and drinking-driving reforms appeared as statistically significant determinants of traffic fatalities (for drivers of all ages).<br>- Moreover, Michigan's MLDA demonstrated a surprisingly strong and long-term effect across all age groups.	- Most importantly, changing the MLDA was the first step Michigan took to improve highway safety. The MLDA was the first regulatory tool employed in Michigan, it passed many years before drinking-and-driving became a hot issue in other states, and it raised the minimum drinking age comprehensively rather than incrementally. Moreover, the MLDA's diminution of fatalities across all age categories and not just its younger target group is explained simply: young intoxicated drivers kill persons in all age groups, not just their own.<br>- To be most fully effective in reducing fatalities among young drivers, a high MLDA may have to be accompanied by effector tools which enhance the certainty of punishment afforded by regulators.	**INCLUDE.**<br><br>- Fatal Accident Reporting System (FARS) data is used from 1975 to 1987 in Michigan.

[94]	**First author, year, country**	**General aim**	**Study design**	**The policy measure(s) or intervention(s) at hand**	**Measured policy effects on MLDA**	**Reflection on policy and other occurrences by authors**	**Comments**
	Hoskin, 1986, USA.	The study reported below was conducted to determine whether recent increases in the legal drinking age in 10 states have had a significant effect on fatal motor-vehicle accidents among drivers affected by the law changes.	The effects of the legal drinking age changes were determined by testing the difference between the ratios of single-vehicle nighttime fatalities to licensed drivers before and after the laws were changed. In addition, ratios for licensed drivers aged 25 to 29 years (individuals not affected by an increase) were examined to determine whether other factors could have influenced the results. The Wilcoxon matched-pairs signed-ranks test, and the t-test was used.	Raises of MLDA in 10 states between 1977 and 1980.	- Results indicated that a statistically significant difference existed between the ratios before and after the drinking age increases, nine of the ten states had decreased single-vehicle nighttime fatalities (SVNF) to 1,000 licensed-drivers-ratios after the change (sig.).<br>- Comparable results on licensed drivers aged 25 to 29 (who were not affected by the law change) showed no statistically significant difference in ratios before and after the law change.<br>- A t-test of the changes in ratios for the affected versus the comparison drivers indicated a statistically significant difference, the differences in ratios from before to after the law change were significantly greater for drivers in the affected age group than for those in the comparison group.	- Using a different age group rather than a matched state as a comparison group is a better control for historical differences between the groups. Other law changes or special enforcement efforts should affect both age groups equally within a state, whereas there may be substantial differences between states.<br>- It should be noted that 4 of the 13 states and the District of Columbia have varying minimums, so the full estimated reduction in the fatality rate may not result. For instance, Colorado allows 3.2% beer at age 18 and other alcoholic beverages at age 21.	**INCLUDE.**<br><br>Data on motor-vehicle fatalities were obtained from the Fatal Accident Reporting System (FARS).

[95]	First author, year, country	General aim	Study design	The policy measure(s) or intervention(s) at hand	Measured policy effects on MLDA	Reflection on policy and other occurrences by authors	Comments
	Legge, 1990, USA.	To explore the impact of four types of policy interventions on traffic safety in New York State between 1975 and 1987.	- Autoregressive Integrated Moving Average (ARIMA) analysis with a "step" function is employed to measure the effect of each intervention.<br>- With regard to analyzing the effect of the DWI reform, single- vehicle fatal crashes involving male drivers at night will be compared to total daytime fatalities.	- The 28 November 1981 law which standardized and increased penalties for drinking and driving.<br>- The legislation which went into effect on 1 December 1982 raising the MLDA from 18 to 19 years of age.<br>- The mandatory seat belt law, effective 1 January 1985.<br>-The legislation which raised the MLDA from 19 to 21 years of age, effective 1 December 1985.	- The strongest impact was demonstrated by the 28 November 1981 DWI reform.<br>- While the effects of the age change laws were not significant (even when subdividing populations and time and day of the fatality), the impact of the seat belt law shows some strength.	- The most significant conclusion which challenges the literature to date, is that a strong law against drinking and driving is not necessarily confined to a short-term effect.<br>- The success of the law is best explained by a combination of strong public support, rigorous law enforcement, and the financing provisions of STOP-DWI. The new law raised the certainty of both conviction and more rigorous financial punishment. In addition, the program is popular because local governments are given considerable liberty in the usage of collected fines, and hence became strong advocates of the law. Another reason why this reform triggered a more permanent change is that groups such as MADD and other alcohol-conscious groups helped to create a climate which placed potential gains to public safety over the benefits of social drinking.<br>- The most disappointing results of the study concern the MLDA reforms. One possible explanation for the poor performance is New York's gradual approach to implementing change (in contrast to a state such as Michigan, which abruptly increased the drinking age from 18 to 21 in 1978). Another explanation could be that given the power of the DWI and seat belt reforms, it may have been difficult to reduce fatalities much further.	**INCLUDE.**<br><br>- Data from the Fatal Accident Reporting System (FARS) were used for measuring traffic fatalities.

[96]	First author, year, country	General aim	Study design	The policy measure(s) or intervention(s) at hand	Measured policy effects on MLDA	Reflection on policy and other occurrences by authors	Comments
	Ruhm, 1995, USA.	- This study investigates the impact of beer taxes and a variety of alcohol-control policies on motor vehicle fatality rates, using fixed-effect models with data for the 48 contiguous states over the 1982 through 1988 time period. Special attention is paid to omitted variables biases resulting from failing to adequately control for grassroots efforts to reduce drunk driving, the enactment of other laws which simultaneously operate to reduce highway fatalities, and the economic conditions existing at the time of the legislation.	The econometric analysis (using fixed-effects models and grouped data logit models) uses data for the 48 contiguous states over the 1982 through 1988 time period. The total vehicle fatality rate, the night-time vehicle fatality rate and night-time vehicle fatality rate of 18 to 20-year-olds are investigated.	- The minimum legal drinking age.<br>- The tax rate on beer.<br>- Preliminary breath test laws.<br>- Dram shop legislation.<br>- Administrative per se regulation.<br>- Implied consent legislation.<br>- Legislation mandating jail or community service for the first DUI conviction.	- Legal drinking ages are strongly negatively related to the fatalities of 18 to 20-year-olds and these estimates appear to be robust to changes in model specifications (sig.).<br>- Higher beer taxes appear to significantly reduce vehicle deaths and the parameter estimates obtained from fixed-effect models are relatively insensitive to the choice of specification.<br>- The predicted reduction in vehicle death rates from raising the minimum drinking age from 18 to 21 declines by 70%, and becomes statistically insignificant, with the addition of covariates for per capita incomes, unemployment rates and five DUI statutes.<br>- In the most fully specified model, dram shop laws are the only regulatory variable with a statistically significant negative impact on traffic mortality.	- This does not imply (dram shop laws being the only regulatory variable with a statistically significant negative impact) that all alcohol-control policies are necessarily ineffective. Future research needs to more carefully control for a comprehensive set of regulatory variables and to account for grassroots activities (e.g., Mothers Against Drunk-Driving).<br>- Stricter alcohol laws, unless draconian in nature, are unlikely to yield a significant further decline in traffic fatalities. The U.S. now has a uniform 21 MLDA and most states have already instituted stringent policies designed to deter drunk-driving. By contrast, substantial decreases in vehicle deaths probably could be obtained by increasing alcohol tax rates, which remain low by historical standards.	**INCLUDE.**<br><br>- Information on traffic mortality was obtained from the National Highway Traffic Safety Administration's Fatal Accident Reporting System (FARS).

[40]	**First author, year, country**	**General aim**	**Study design**	**The policy measure(s) or intervention(s) at hand**	**Measured policy effects on MLDA**	**Reflection on policy and other occurrences by authors**	**Comments**
	Rock, 1991, USA.	The impact on traffic accidents of changes in the drinking age in Illinois was studied.	- A data set suitable to analyze the impact of these changes is compiled monthly by the IDOT. For the entire state from 1970 to mid- 1989, traffic accident totals (also broken down by fatal, injury, or property damage only), age of drivers involved, and road conditions were selected.<br>- Using auto-regressive integrated moving average (ARIMA) techniques and total accidents by month over a 20-year period, the experience in Illinois is reexamined.	Illinois lowered their drinking age for beer and wine from 1973 to 1980 to 19 and raised it in 1980 from 19 to a uniform MLDA of 21.	- The lower drinking age in Illinois was responsible for an increase of more than 5,000 accidents per month, a 14 percent increase.<br>- When the age was raised back to 21 in 1980, the figures reversed a similar amount (sig.).	- The Illinois data set could be used to examine other areas of interest and controversy. For example, it is alleged that those immediately under the drinking age are more likely to obtain alcohol illegally than those who are farther below. With an increase of the drinking age to 21, one could examine the percentage reductions in accidents in the younger age groups.<br>- A variety of other public policies could be examined. Laws enacted in Illinois have allowed right turn on red (1973), lowered the maximum speed limit to 55 mph (1974), raised the maximum speed limit on most Interstate highway segments to 65 mph (1987), and mandated the use of seat belts (1985).<br>- Changes in the drinking age in bordering states are relevant; for a number of years the lower drinking age in Wisconsin attracted many younger drivers from Illinois.<br>- The short- versus long-term effects can differ: short-term impacts can dissipate if below-age drinkers obtain alternative sources of alcohol, or they can be reinforced if new cohorts of younger drivers do not develop patterns of driving after drinking.<br>- An alternative explanation, raising the drinking age does not reduce fatalities, more lives are lost among older drivers than are saved among younger drivers. An extension to this was that inexperience in drinking, independent of age, was the major hazard.	**INCLUDE.**<br><br>- The Illinois Department of Transportation (IDOT) data was used.

[42]	First author, year, country	General aim	Study design	The policy measure(s) or intervention(s) at hand	Measured policy effects on MLDA	Reflection on policy and other occurrences by authors	Comments
	Bolotin, 1985, USA.	The third objective of this paper is to evaluate the health impacts of a national MLDA by attempting to provide a critical test of the suggested 'proven solution' (i.e., health impact of a national MLDA) based upon an evaluation of previous studies and our own analyses. To estimate the likely effects, we focus on the subject of teenage alcohol-related traffic accidents.	- Data are analyzed across the states for annual time points from 1977-1984. Based upon these analyses, it will be possible to compare patterns of behavior among states which raised the drinking age during this time period and states which maintained a constant drinking age for all of the relevant years.<br>- Focus will be on fatal crashes which are classified as being alcohol-related (as a yearly figure).<br>- Of primary interest will be fatalities among the affected population group (ages 18-20), the group which is affected directly by an increase in the MLDA.<br>- 21-25-year-olds are used as a control group.<br>- Those under 18 are used as a comparison group.<br>- Each state which changed its drinking age is paired with a state which maintained a constant drinking age throughout this period, selected on geographical and cultural proximity to the changes state.	States raising the MLDA between 1977 and 1984.	- The highest percentage of alcohol-related traffic deaths involved drivers in the 21-25-year-old age group. The nationalization of the MLDA of 21 will not affect the group (18-20-year-olds) which contributes most to the problem.<br>- Nationwide (summing up all 24 states), the percent of alcohol-related fatalities in the affected group increased very slightly (from 41 to 43) after the raise.<br>- Only in the states of New York and Texas, the desired decline after a raise of the MLDA occurred (and differences between affected and unaffected groups are in the desired direction).	- The establishment of a national MLDA of 21 does not seem to provide even a partial solution to the problem of drunk driving, at least not based on the results of this study. The analyses do not distinguish any significant declines in alcohol-related traffic deaths for the 18-20-year-olds affected by the increase of the MLDA.<br>- Prior studies have concentrated their analyses on numerical, as contrasted with proportional (as is conducted in this study), measures of the problem. Therefore, the relationship between an increase in MLDA and the number of fatalities may be spurious.<br>- Prior studies not consider alcohol-related fatalities, but rather, the time of day when the accident occurs (nighttime fatal crashes). By relying on this approach, the authors simply analyze the actual number of fatal accidents that occur at this time.<br>- We recommend policies which are more general in their application (i.e., not only 18-20-year-olds). Also, a more systematic investigation of alternative policy strategies is needed (e.g., investigating mandatory imprisonment or license suspension laws).	INCLUDE.<br><br>- Data from the Fatal Accident Reporting System (FARS).<br><br>- Only the third main aim of this study is extracted, since the first two aims involved a political strategy analysis.

[44]	First author, year, country	General aim	Study design	The policy measure(s) or intervention(s) at hand	Measured policy effects on MLDA	Reflection on policy and other occurrences by authors	Comments
	Hingson, 1983, USA.	- This paper examines the impact of raising the drinking age on teenage drinking, driving after drinking, and non-fatal accident involvement in Massachusetts prior to the law's enactment and twice at yearly intervals after the law enactment.<br>- Massachusetts was compared with New York State, who retained an 18-year-old drinking age.	- Random telephone surveys with approximately 1,000 16-19-year-olds in each state were undertaken prior to the law's enactment and twice at yearly intervals after the law, to assess the law's impact on teenage drinking, driving after drinking, and non-fatal accident involvement.<br>- Respondents were asked where they most often obtained their alcohol (e.g., liquor/grocery store, bar/club, at home) and where they drank > 5 consumptions in the last month (e.g., party, bar).<br>- Drinking was measured asking for 'any drinking' in the last month and 'drinking 6+ drinks at one time' in the last month.<br>- Log-linear analysis was used on the survey data.<br>- Fatal crash data reported to the US Department of Transportation by each state from 1976-1981 were also analyzed.<br>- To assess law enforcement practices and problems, interviews were conducted with over 50 Massachusetts police officers representing all levels of command in urban, rural, and suburban jurisdictions.	- Legislation raising the legal drinking age in Massachusetts from 18 to 20 in 1979.<br>- At the time Massachusetts raised its legal drinking age from 18 to 20, the two states had similar laws regarding age of driving licensure and penalties for driving while intoxicated.	- After the law, the frequency of teenage drinking in bars and clubs and the percentage of teenagers reporting they most often obtained their alcohol in liquor stores and groceries dropped in Massachusetts compared to New York. (sig.).<br>- During the two years after the law, the proportion of Massachusetts teenagers who had someone else purchase alcohol for them or who most often obtained alcohol from their homes nearly doubled (sig.).<br>- The average daily consumption of alcohol in the 16-19-year-old age group during the two years after the law did not decline in Massachusetts compared to New York. Nor did teenagers report shifts to the use of other psychoactive drugs.<br>- After the law, the proportion of teenagers who reported driving after drinking heavily (six or more drinks) did not decline in either state. However, the frequency that teenagers reported they drove after any drinking declined significantly more in Massachusetts.<br>- The three methods of statistical analysis indicated no significant difference between the two states in the overall teenage fatal accident trends (not sig.).<br>- The frequency with which teenagers were arrested for driving under the influence did not significantly change in Massachusetts during the first year after the law compared to the previous two years.<br>- Enforcement of the law focusing on the sellers was minimal and sporadic.<br>- The intensity of enforcement varied widely from community to community.<br>- The reasons most often cited for the variability in enforcement of the law among communities across the state was the lack of personnel and competing priorities, particularly in some high crime inner-city jurisdictions. Moreover, many officers did not perceive teenage purchasing of alcohol or drinking per se as a sufficiently serious crime to stigmatize<br>Juveniles by putting an arrest on their records.	- The study examined the first two years following enactment of the law. During this time period, the 18 and 19-year-old age groups who had previously been allowed to drink had that privilege revoked. One could hypothesize that the previous drinking habits of this group would be resistant to change.<br>- The state's law provides a symbolic statement to teenagers that its citizens disapprove of their drinking, and fears the accidents they may cause when they drive after drinking. The study results prompt us to ask whether the law could have had a greater impact among all Massachusetts teenagers if enforcement efforts were more consistent and if greater attention were paid to preventing the common practices of non-teenagers purchasing alcohol for teenagers or liquor outlets not requiring age identification. Without sufficient resources and coordination of enforcement efforts, those police who actively strive to enforce the law in one community may find their efforts negated by minimal enforcement in the next.<br>- Lack of community resources and variable willingness to enforce laws focused on teenagers raise questions about whether alternative strategies such as increased enforcement of the drunk driving and traffic safety laws aimed at all drivers, or requirements for safer cars and improved road design would yield greater reductions in nonfatal and fatal accidents both among teenagers and non-teenagers.	**INCLUDE.**<br><br>- In addition to telephone surveys, data from the Fatality Analysis Reporting System (FARS) was used.

[45]	First author, year, country	General aim	Study design	The policy measure(s) or intervention(s) at hand	Measured policy effects on MLDA	Reflection on policy and other occurrences by authors	Comments
	Vingilis, 1981, Canada.	This paper reports four types of data which are relevant to the study of the effects of the increase in the MLDA.	Data are from:<br>1) surveys of drinking and drinking problems among high school students (measuring: 1) alcohol use in the month prior, 2) feeling 'tight' at least once in the month prior, 3) 'drunk' at least once in the month prior, 4) 5 or more drinks on a singes occasion at least once in the month prior). Conducted in 1977 and 1979, stratified proportional sample in the province. Log linear analyses.<br>2) a study (surveys) of perceptions of vice-principals. Conducted in 1972 and 1980.<br>3) a trend analyses of young drinking offender charges; and<br>4) trend analyses of drinking-driving statistics and driver fatalities. Two types of drinking-driving statistics were obtained: monthly Ontario drinking-driving convictions form 1977 and 1979 for 16-21-year-olds and monthly Ontario accident fatalities from 1973 to 1979 for 16-21-year-olds. Time series analyses were used.	The increase of the MLDA in 1979 in Ontario (from 18 to 19).	1) the proportion of drinkers among 18-19-year-olds decreased from 1977 to 1979 (as well as a decrease in the proportion feeling 'tight'). Comparable effects were observed for younger drinkers (under 18) in the exact opposite direction.<br>2) The findings from this study indicate that the vice-principals have perceived either no change or less student drinking and alcohol-related problems at their school since the increase in legal drinking age (between 20 per cent and 30 per cent of the vice-principals reported a decrease in drink-related behaviors).<br>4) There were no significant differences between the pre-intervention and post-intervention time periods for both types of drinking-driving statistics.	- The results of the four studies indicate few statistically significant changes, however, these findings seem to tentatively suggest a minimal effect for 18-19-year-old high school students, but not for the regular (once a week or more) or younger drinkers.<br>- The fact that regular drinkers reported no changes in their drinking habits could partially explain why major changes were not observed for the more general methods of measurement, that is, the rates of accidents, charges and convictions and the perceptions of vice-principals.<br>- Additionally, a large proportion of Ontario youths are not in school and these non-students may be heavier drinkers. Conviction and fatality statistics are not sensitive enough measures, as it seems that too few high-accident-and-arrest-risk youths were changing their drinking patterns for an effect to emerge from these statistics.<br>- The minimal Ontario effect was expected, as it was felt that the one-year increase was not sufficient to cause a major impact on youthful drinking behavior.	INCLUDE.<br><br>- Data from the Fatal Accident Reporting System (FARS).<br><br>- Only 1, 2 and 4 approved criteria and are extracted.

[51]	First author, year, country	General aim	Study design	The policy measure(s) or intervention(s) at hand	Measured policy effects on MLDA	Reflection on policy and other occurrences by authors	Comments
	Hingson, 1985, USA.	This study explores whether, when Massachusetts raised its legal drinking age from 18 to 20 in early 1979, significant declines occurred in that state in 1) teenage drinking and in turn, 2) homicide rates, 3) suicide rates, and 4) deaths from nontraffic accidents in 1980, 1981 and 1982 relative to New York State, where the legal drinking age remained at 18 during that time.	- An anonymous random digit dialing telephone survey of 16 to 19-year-olds was conducted in Massachusetts and New York (control), prior to enactment of the law in 1979, asking teenagers about their personal characteristics, drinking practices (average drinks daily), procurement of alcohol, use of psychoactive drugs and possible experiences with police enforcement when obtaining alcohol. Twice and yearly intervals following the law were conducted (six waves for each state). Log linear analyses was used to test the impact of the law on dependent variables.<br>- Interviews were conducted with 50 Massachusetts police officers and inspectors in all levels of command and context, asking them about enforcement practices before and after the drinking age change.<br>- Arrest data from the Uniform Crime Reporting system were evaluated before and after the law change for both states.<br>- The number of fatalities from non-traffic accidents, suicides, and homicides were analyzed separately, collating data into pre- and post-law groups. The number of fatalities was fit to a log linear model.	Raise of the legal drinking age from 18 to 20 in early 1979 in Massachusetts.	- After the law, the frequency of teenage drinking in bars-clubs-restaurants and the percentage of teenagers reporting they most often obtained their alcohol in liquor stores and groceries dropped in Massachusetts compared with New York (sig.).<br>- One third attempting to purchase liquor indicated they were never asked for ID, 5% were stopped by the police just once, none were arrested the first year after the law.<br>- Two years after the law, the proportion of Massachusetts teenagers who had someone else purchase alcohol for them nearly doubled (sig.).<br>- The average daily consumption of alcohol in the target population (15 to 19-year-olds) two years after the law did not significantly decline in Massachusetts compared to New York.<br>- After the law change, arrests among the target population rose over 150% (for multiple alcohol-related offenses).<br>- The intensity of enforcement was varied between communities caused by lack of personnel and competing priorities (as the main reasons). Also, many officers did not perceive underage drinking or purchasing alcohol as a serious crime.<br>- During the three years after Massachusetts raised its drinking age, compared with New York, there were no significant changes among the target population in the number of deaths from: 1) accidental injury other than traffic accidents, 2) the number of suicides or 3) homicide deaths.	- This study found no reductions in non-motor vehicle accident deaths, homicides, or suicides relative to New York among 15 to 19-year-olds after Massachusetts raised its drinking age from 18 to 20.<br>- It is possible that some types of nontraffic accidents, homicides, and suicide are also more likely than others to involve alcohol, but the possible relations are not well established and hence the specific subcategories of violent death are not monitored over time.<br>- Despite similarities between states, it is possible that one of the two experienced a change that could confound a change in violent death rates.<br>- The actual contribution that drinking makes is quite small relative to other independent predictors of other causes of death.<br>- It is conceivable that changes in location of drinking (e.g., form bars to drinking at home) might reduce the frequency of nighttime travel and rates of fatal crashes.	INCLUDE.<br><br>- An anonymous random digit dialing telephone survey of 16 to 19-year-olds.<br>- Interviews with 50 Massachusetts police officers and inspectors.<br>- Arrest data from the Uniform Crime Reporting system.<br>- The number of fatalities from non-traffic accidents, suicides, and homicides.

[57]	First author, year, country	General aim	Study design	The policy measure(s) or intervention(s) at hand	Measured policy effects on MLDA	Reflection on policy and other occurrences by authors	Comments
	Miron, 2009, USA.	We challenge the view that MLDAs reduce traffic fatalities based on three findings. First, the overall impact estimated in earlier research is driven by states that increased their MLDA prior to any inducement from the federal government. Second, even in early-adopting states, the impact of the MLDA did not persist much past the year of adoption. Third, the MLDA has at most a minor impact on teen drinking.	- We examine the relation between MLDAs and traffic fatalities using aggregate road facilities data of the complete population and of 15-24-year-olds and state-level panel data, reconstructing the analysis of Dee (1999) (using FARS to construct a panel data set for the 48 contiguous states over the period 1977–1992) and extending it to include Alaska, Hawaii, and Washington DC, and the years 1976 and 1993–2005. We focus on 18- to 20-yr-old fatalities.<br>- Using MTF data, we employ the two specific measures common in the literature, ‘‘drinker’’ (having any drink of alcohol in the last month) and ‘‘heavy episodic drinker’’ (having five or more drinks in a row at some point in the last 2 weeks). We also examine the number of motor vehicle accidents that respondents report as occurring after consuming alcohol. We estimate regressions similar to the traffic fatality calculations with these dependent variables. The measure of the MLDA is identical to that used in previous literature, a dummy for having a drinking age of 18 years.	- Changes in de MLDA over the period 1976-2005.<br>- Mandatory seatbelt law.<br>- The BAC limit for legal driving.<br>- Beer taxes.<br><br>We omit several potentially relevant policies, in part because of data availability, in part to conform with Dee (1999), and in part because previous studies have found limited evidence of any impact on TFRs. These variables include dram shop liability laws, mandatory sentences for driving under the influence, sobriety check points, anti-plea-bargaining statutes, changes in tort liability laws that place greater responsibility with intoxicated drivers, happy hour regulations, enforcement (however, enforcement is too low to have any impact on the results examined) and alcohol education programs.	- The MLDA fails to have the fatality-reducing effects that previous articles have reported; trends between 1910 and 2000 in aggregate data of road facility rates show no difference in trends between 15-24-year-olds and that of the entire population.<br>- The declines in the total and 15–24 TFR that began around 1969 long precede the adoptions of an MLDA of 21 in the mid-1980s. The key fact about TFRs, therefore, is that they have been trending downward for decades and have been poorly correlated with the MLDAs.<br>- State-level panel data for the past 30 years show that any nationwide impact of the MLDA is driven by states that increased their MLDA prior to any inducement from the federal government (FUDAA). Even in early-adopting states, the impact of the MLDA did not persist much past the year of adoption.<br>- The MLDA appears to have only a minor impact on teen drinking (sig.), these reductions derive mainly from states that adopted the MLDA21 before enactment of the FUDAA.<br>- Nevertheless, when the number of accidents post-alcohol consumption was analyzed, the panel estimates reveal that movement away from an MLDA of 18 is associated with a statistically insignificant change in reporting of alcohol-related traffic accidents.	- These results suggest that, at most, the MLDA21 reduced TFR18–20 in states that adopted the policy on their own. This raises the question of endogeneity. The MLDA21 in these states may have been enacted in response to grassroots concern against drunk driving or implemented alongside other efforts to reduce traffic fatalities. Relatedly, states that adopted on their own may have been states that devoted significant resources to enforcement.<br>- The limited effects found in non-early adopting additionally challenges the desirability of coercive federalism.<br>- Landmark improvements in the accident avoidance and crash protection features of passenger cars (in place around 1970), and control for advances in medical technology might explain the drastic reductions in traffic fatalities over the past half century and should be made to additionally measure and control for as they are likely to be major determinants of the declining traffic fatality trends.	**INCLUDE.**<br><br>- Data from the Fatal Accident Reporting System (FARS) were used.<br>- Data from Monitoring the Future (MTF) surveys were used.

[62]	First author, year, country	General aim	Study design	The policy measure(s) or intervention(s) at hand	Measured policy effects on MLDA	Reflection on policy and other occurrences by authors	Comments
	Smith, 1984, USA.	The effects on the raised MLDA in Massachusetts is examined on drinking, drinking and driving, and nonfatal and fatal crash involvement of 16-17-year-olds (teenagers immediately younger than those targeted by the law).	- Data from Massachusetts are compared with those from New York, where the MLDA remained at 18.<br>- A total of 3 years of survey data from the two states and 6 years of data from FARS were used for pre- and post-law comparisons.<br>- An anonymous random digit-dialing telephone survey was conducted in Massachusetts prior to enactment of the law in 1979, asking teenagers about personal characteristics, drinking practices, procurement of alcohol, use of psychoactive drugs, driving after drinking and nonfatal accident involvement. A similar survey was conducted in the New York area (respondents were asked for their drinking behavior and behavior on how they obtained their alcohol comparable to the New York sample).<br>- Twice at yearly intervals following enactment of the law, surveys of similar size were repeated in each state.<br>- Log-linear analysis was used on the survey data.<br>- In addition, FARS data was used for both states from 1976 to 1982 (single-vehicle nighttime accidents were examined separately).<br>- Data were fitted to a log-linear model, an analysis of variance and an analysis of covariance.	The 1979 Massachusetts law raising the MLDA from 18 to 20.	- The findings suggest that raising the MLDA had minimal effects on the drinking behavior of Massachusetts teenagers.<br>- There was a significant decline in Massachusetts in the number of teenagers reporting drinking at a bar/club/restaurant or in an automobile, an increase of teenagers drinking at parties and a decrease in the number of teenagers purchasing alcohol at liquor stores after the raise of the MLDA and compared to New York.<br>- Massachusetts 16-17-year-olds were more likely in each of the 2 post-law years to have had others purchasing alcohol for them (compared to New York).<br>- After the enactment of the law, driving after drinking declined significantly in Massachusetts relative to New York.<br>- Our analysis did not reveal a significant difference in single-vehicle nighttime fatal accidents (and total fatal accidents) among 16-17-year-olds in Massachusetts and New York after the enactment of the law.	- The present findings suggest that the effect of the MLDA changed where these teenagers drink and how they obtain alcohol.<br>- It is interesting that both before and after enactment of the law, 16-17-year-olds in New York drove less frequently after drinking compared to Massachusetts teenagers. Perhaps, this is the result of having a stable (but lower) drinking age in New York over several decades.<br>- Changes in drinking age may offer some reduction in teenage traffic crash involvement, but teenage drinking and teenage driving after drinking remain serious problems, even in states that raise their MLDA.<br>- The present study indicates lawbreaking among young people, perhaps fostering cynicism toward the legislative process and disregard for law enforcement. As long as teenagers are never asked for ID or have others purchase alcohol for them, significant declines in harm are unlikely.	**INCLUDE.**<br><br>- Data from the Fatal Accident Reporting System (FARS).<br><br>- Hingson et al. (1983) examined the first 2 post-law years in Massachusetts [44].

[97]	First author, year, country	General aim	Study design	The policy measure(s) or intervention(s) at hand	Measured policy effects on MLDA	Reflection on policy and other occurrences by authors	Comments
	Grossman, 1986, USA.	- A primary purpose of this paper is to investigate the responsiveness of motor vehicle death rates of youths aged 15 through 24 to variations in the cost of beer as reflected by differences in state excise tax rates on beer.<br>- We also examined the effect of an increase in the legal drinking age on youth motor vehicle deaths.	The empirical research is based on a time series of state cross sections for the period from 1975 through 1981. Logit motor vehicle death rate regressions are obtained for three age groups: youths aged 15-17, youths aged 18-20, and youths aged 21-24.	- Real beer tax (sum of Federal and state excise taxes; price of alcohol).<br>- Beer legal drinking age.	- Negative and statistically significant real beer tax effects are obtained for youths aged 15 through 17, 18 through 20, and 21 through 24 (the elasticity of the death rate with respect to the real beer tax is -.09 for the youngest age group and -.17 for the other two age groups).<br>- Negative and statistically significant legal drinking age effects are obtained for youths aged 18 through 20<br>- Effects of taxes and MLDA are not affected by the inclusion of drinking sentiment (e.g., 'wet' counties permitting the sale of alcoholic beverages and religious background) proxies.	- That effects are not affected by drinking sentiment proxies means that the tax and MLDA effects emphasized here are not artifacts of the endogeneity of state laws and decision-making.<br>- If reductions in youth motor vehicle accident deaths are desired, both uniform MLDA of 21 and increase in the federal excise tax rate on beer are effective policies to accomplish this goal.<br>- Results also suggest that the tax policy may be more potent than the drinking age policy and may additionally reduce fatal crashes involving adults (as opposed to MLDA primarily focusing on youths).<br>- Excise tax hikes impose welfare costs on all segments of the population, MLDA is targeted at the group that accounts for a disproportionate share of motor vehicle accidents and deaths. However, enforcement and administrative costs associated with a uniform MLDA of 21 may exceed those associated with the tax policy.<br>- If substantial resources must be allocated to raising these probabilities (deterring offenses of drunken drivers), the excise tax policy may be preferable to or complementary with a system of large fines.	**INCLUDE.**<br><br>- Information on traffic mortality was obtained from the National Highway Traffic Safety Administration's Fatal Accident Reporting System (FARS).

[98]	**First author, year, country**	**General aim**	**Study design**	**The policy measure(s) or intervention(s) at hand**	**Measured policy effects on MLDA**	**Reflection on policy and other occurrences by authors**	**Comments**
	Saffer, 1986, USA.	The purpose of this paper is to estimate the responsiveness of youth motor vehicle fatality rates to increases in the legal drinking age and to variations in the cost of beer.	The data set employed is time series, from 1975 to 1981, of cross sections of the 48 contiguous states. Separate regressions for 15 to 17-year-olds 18 to 20-year-olds and 21 to 24-year-olds are presented (and a simultaneous estimation model is used to account for the endogeneity of the drinking age).	- Real beer tax (sum of Federal and state excise taxes; price of alcohol).<br>- Beer legal drinking age.	- The econometric results show that the drinking age and beer tax both have a significant influence on youth motor vehicle mortality.<br>- The drinking age is assumed to be endogenous in the methodology used to compute these estimates.	- The econometric results show that mortality has a significant causal effect on the drinking age and that ignoring the problem of endogeneity results in underestimation of the effects of this policy variable.	**INCLUDE.**<br><br>- Information on traffic mortality was obtained from the National Highway Traffic Safety Administration's Fatal Accident Reporting System (FARS).

[99]	First author, year, country	General aim	Study design	The policy measure(s) or intervention(s) at hand	Measured policy effects on MLDA	Reflection on policy and other occurrences by authors	Comments
	Williams, 1983, USA.	In this paper, a study of the effect of raising the drinking age on fatal crashes involving teenage drivers is reported.	- Data on driver involvement in fatal crashes from 1975 through 1980 were obtained.<br>- The duration of post law periods studied ranged from nine months (Illinois) to three years (Minnesota).<br>- Each of the nine states was paired with a comparison state in which the legal minimum drinking age remained unchanged during the study period. Comparison states were chosen on the basis of geographic proximity to law-change states and comparability with law-change states with respect to numbers of crash fatalities.	Nine states, all of which raised their legal minimum drinking ages between 1976 and 1980, were studied.	- The results of the present study indicate that when states raise the drinking age, there is a corresponding decrease in fatal crashes among law-affected drivers (estimated reductions in nighttime fatal crashes ranged from 18 to 28 percent; all estimates were statistically significant).<br>- There is some evidence that raising the drinking age also affects younger drivers, but the reductions in the involvement of younger drivers in fatal crashes were not statistically significant.	The societal benefits achieved in states that have raised their drinking ages are substantial; the benefits achievable if additional states raise their drinking ages would be even more substantial. Raising the legal minimum drinking age to 21 in all states would go far toward reducing the annual toll of motor vehicle deaths in the United States, particularly the deaths of young people and of others with whom they are involved in crashes.	**INCLUDE.**<br><br>- The National Highway Traffic Safety Administration's Fatal Accident Reporting System (FARS) were used.

[100]	First author, year, country	General aim	Study design	The policy measure(s) or intervention(s) at hand	Measured policy effects on MLDA	Reflection on policy and other occurrences by authors	Comments
	Filkins, 1981, USA.	- Twelve years of Michigan accident data, assess the impact of lowering the minimum legal drinking age from 21 to 18 in 1972, and subsequently raising it back to age 21 in 1978.<br>- DUIL (Driving Under the Influence of Liquor) arrest data were also obtained and analyzed for 1978 and 1979.	- All accidents occurring in Michigan that were investigated by police agencies and reported on official forms were obtained for the twelve years from<br>1968 through 1979.<br>- Driver age and the presence or absence of drinking in the accident were the key variables, and partitioning the chi-square statistic into its degrees of freedom was the primary analytic technique.	Lowering the minimum legal drinking age from 21 to 18 in 1972, and subsequently raising it back to age 21 in 1978, and DUIL arrest data (for 1978 and 1979).	- The results show clearly that the minimum legal drinking age influences drinking-driving patterns among the affected age groups. Alcohol related accidents increased among 18- to 20-year-old drivers when the legal drinking age was reduced to 18, and non-fatal accidents decreased when it was later increased to 21 (sig.).<br>- Analysis of the DUIL arrest data also demonstrates clearly that the recently increased legal drinking age altered drinking-driving practices among the affected drivers (not sig.). The 18-, 19-, and 20-year-old drivers, whether considered singly or as a group, consistently showed fewer arrests in 1979 than in 1978.	In addition, there have been changes in the larger context which influence drinking and driving patterns, both singly and in combination. One can cite the energy crisis of the mid-1970's and the recent economic downturn. These perturbations, of course, preclude simple before-after comparisons of only the affected age groups in the analytical and inferential work, but they cannot in any sense be used to dismiss the findings out of hand.	**INCLUDE.**<br><br>- Michigan accident data.<br>- Arrest data for DUIL.

[101]	First author, year, country	General aim	Study design	The policy measure(s) or intervention(s) at hand	Measured policy effects on MLDA	Reflection on policy and other occurrences by authors	Comments
	Legge, 1994, USA.	The purpose of this paper is to provide a pooled cross-sectional time series analysis of state policies to deter alcohol-impaired driving.	A cross-sectional regression model pooled across three years (1980, 1984 and 1987) was used encompassing both environmental and policy variables.	- Per se laws.<br>- Preliminary breath tests.<br>- Mandatory jail sentences for the first offense.<br>- Fines for first time offenders.<br>- Mandatory administrative license suspension for the first offense.<br>- MLDA.<br>- Mandatory seat belt laws.<br>- Motor vehicle safety inspection laws.	- All policy variables impacting single-vehicle nighttime fatalities are concentrated outside of the severity component.<br>- The findings indicate that less punitive but more certain and swift punishments have had the largest effective impact on alcohol-related crashes (for instance, the per se laws and administrative license suspensions, as opposed to mandatory jail terms, increased fines and license suspensions).<br>- The MLDA has a strong and significant effect on alcohol-related crashes (even when analyses are restricted to those fatalities involving drivers under 21).	- In addition to laws intended to deter alcohol-impaired driving, traffic casualties may be determined by other public policies as well as environmental factors, some of which are beyond the direct control of policymakers. Each state has its own traffic patterns and safety which may expose citizens to varying risks for accidents.<br>- The limited impact of the 1980s legislation is that most laws were not part of a comprehensive package, but were passed in a piecemeal fashion with limited information on the potential effectiveness of alternative laws.<br>- What was missing in nearly all states was a coordinated and carefully planned policy design and implementation strategy to impact the highway safety problem.<br>- An alternative approach is to focus on specific deterrence rather than passing laws targeted at society as a whole, for instance, considering drunk driving as being due to alcohol abuse as opposed to social drinking.	**INCLUDE.**<br><br>- Data from the Fatal Accident Reporting System (FARS) in 1980, 1984 and 1987 were used as a proxy measure for alcohol-related fatalities.

[102]	**First author, year, country**	**General aim**	**Study design**	**The policy measure(s) or intervention(s) at hand**	**Measured policy effects on MLDA**	**Reflection on policy and other occurrences by authors**	**Comments**
	Colon, 1984, USA.	The relationship between the alcohol beverage purchase age and single-vehicle fatalities is examined.	- The 50 states and the District of Columbia were used in cross-sectional analyses for the year 1976. Two separate analyses were performed: a bivariate analysis that cross- tabulated the beverage purchase age and single-vehicle fatalities and a multiple regression analysis in which the single vehicle fatality rate was the dependent variable.	The MLDA in 50 states and the District of Columbia in the year 1976.	- Support was found for a positive association between the purchase age and single motor vehicle fatalities. Since the purchase age variable and the purchase age concept are inversely related (i.e., as the purchase age is reduced, the drinking age population increases), the hypothesis of a positive relationship between purchase age and traffic fatalities is supported.	- An alternative explanation for this finding is that minors residing in a locality with a high purchase age drive to adjacent localities where they can purchase alcoholic beverages legally.<br>- It appears that the present state of affairs, where each locality sets its own purchase age independently, exacerbates highway fatalities. A uniform national purchase age is suggested as a means of dealing with this problem.	**INCLUDE.**<br><br>**-**

[103]	First author, year, country	General aim	Study design	The policy measure(s) or intervention(s) at hand	Measured policy effects on MLDA	Reflection on policy and other occurrences by authors	Comments
	Arnold, 1985, USA.	This paper reports the changes in annual driver involvements in fatal crashes that accompanied states that raised their legal drinking age.	Comparing the number of observed fatal accident involvements per year per affected driver after the law change to the number before the law change, using young drivers not affected by the law change as control.	- Raises of the minimum legal drinking ages during 1975 through 1982 in 13 states in the US.<br>- Prior to the 1980 law change, home-rule units in Illinois had the authority to deviate from the State provision which was: 19 for beer and wine, 21 for distilled spirits. After the law change the minimum age was uniformly 21 for all beverages.<br>- In Iowa, Minnesota and New Jersey, a grandfather clause permitted 18-year-olds to drink if they were 18 before the law went into effect.	An effect of raising the legal minimum drinking age is estimated to be a reduction of about 13% in annual driver involvements in fatal accidents per licensed driver affected by the law change (95% CI of 6% and 19%). In three states, changes were statistically significant.	-	**INCLUDE.**<br>- The number of driver involvements in each class is from the Fatal Accident Reporting System (FARS).<br>- The number of licensed drivers is from a compilation by the Federal Highway Administration of data provided by the states.

[104]	**First author, year, country**	**General aim**	**Study design**	**The policy measure(s) or intervention(s) at hand**	**Measured policy effects on MLDA**	**Reflection on policy and other occurrences by authors**	**Comments**
	Saffer, 1987, USA.	The purpose of this paper is to estimate the responsiveness of youth motor vehicle fatality rates to increases in the MLDA and to variations in the cost of beer.	- This study uses time-series data of 48 state cross-sections from 1975 through 1981 and estimates separate fatality regressions for youths fifteen to seventeen, eighteen to twenty and twenty-one to twenty-four.	- The study employs a sample period in which states raised their MLDA.<br>- Real beer tax.	- The results for the single equation mortality model show that the real beer tax and the MLDA are negative and significant on motor vehicle death rates *for youths eighteen to twenty.*<br>- Using comparable analysis, the MLDA is significant in the *fifteen- to seventeen-year-old group* as well.<br>- For *twenty-one to twenty-four-year-olds*, the higher MLDA is insignificant.<br>- For the abovementioned two age groups, the real beer tax is negative and significant.	- The econometric results show that mortality has a significant causal effect on the MLDA and that ignoring the problem of endogeneity results in underestimation of the effects of this policy variable (the empirical evidence supports the endogeneity assumption).<br>- It should be noted that while MLDA and beer tax policies can reduce highway mortality, both policies also subject the innocent to punitive action. Economists have shown that the optimal method of deterring offenses is a program of certain and appropriate sanctions against the guilty. However, high cost of increasing the probability of apprehension could limit the desirability of a program of specific sanctions.	**INCLUDE.**<br><br>- Data from the Fatal Accident Reporting System (FARS).

[105]	**First author, year, country**	**General aim**	**Study design**	**The policy measure(s) or intervention(s) at hand**	**Measured policy effects on MLDA**	**Reflection on policy and other occurrences by authors**	**Comments**
	Maxwell, 1981, USA.	This report contains an analysis of the impact of the raised legal drinking age in Illinois on traffic accidents.	- Monthly single vehicle night male driver involvements (in traffic accidents) from 1977-1980 were analyzed, as the impact measure.<br>- Box-Tiao Intervention Analysis was used (using auto-regressive integrated moving average (ARIMA)) to develop impact assessment models relating the change in driver involvements to the implementation of the raised legal drinking age.	Illinois raised its legal drinking age in 1980 from 19 to 21.	An 8.8 percent reduction in single vehicle night male driver involvements was found for drivers in the affected age groups (sig.).	The raising of the legal drinking age law has been effective in the reduction of single vehicle night male driver involvements for drivers ages 19 and 20 in Illinois.	**INCLUDE.**<br><br>-

[106]	**First author, year, country**	**General aim**	**Study design**	**The policy measure(s) or intervention(s) at hand**	**Measured policy effects on MLDA**	**Reflection on policy and other occurrences by authors**	**Comments**
	Klein, 1981, USA.	This report analyses the effect of raising the MLDA in Maine from 18 to 20 years of age on night male driver involvements and single vehicle night male traffic accidents.	- Accident data was obtained from the state of Maine between 1974 and 1979, and was broken down by sex, time of day and age of driver.<br>- Single vehicle night male accidents, night male driver involvements and single vehicle day male accidents (for comparison) were used.<br>- Night male driver involvements and single vehicle night male accidents were analyzed as a proxy measure for alcohol involvement by age, using intervention analysis.<br>- Box-Tiao Intervention Analysis was used.	Raising the MLDA in Maine from 18 to 20 years of age.	- Results found reductions of 18.6% and 13.9% for night male driver involvements by 18- and 19-year-olds, respectively (sig.).<br>- There was a reduction of 19.9% single vehicle night male accidents for age 18 (sig.).	Possible factors that may have confounded the effect of the law change are:<br>1) Availability of alcohol to 19-year-olds due to older peers within groups.<br>2) Some 19-year-olds may not be 'carded' when attempting to purchase liquor, this dampening the effect.<br>3) The close proximity of 19-year-olds to the higher MLDA.<br>4) A possible 'real' effect of the law on daytime single vehicle accidents by 18-year-olds due to reduced availability of alcohol.<br>5) Drinking among teenagers in the late afternoon resulting in a daytime accident.	**INCLUDE.**<br><br>- Data from the Fatal Accident Reporting System (FARS).

[107]	**First author, year, country**	**General aim**	**Study design**	**The policy measure(s) or intervention(s) at hand**	**Measured policy effects on MLDA**	**Reflection on policy and other occurrences by authors**	**Comments**
	Young, 2000, USA.	We examine the relationship between motor-vehicle fatalities and alcohol taxes, prices, and various drinking laws.	- Using regression models, we estimate the relationships between motor-vehicle fatality rates and beer taxes, prices, and other explanatory variables using data across states and over time in the 1980s, based on data from 48 states over 9 years.<br>- We estimate state-specific fixed effects, estimate both logistic and linear functional forms, and we employ technically superior weights computed from the fitted values of first-stage regressions.	- Federal and state excise taxes on beer.<br>- Retail price of beer.<br>- Minimum legal age for purchase and consumption of beer.<br>- Preliminary breath test.<br>- Lawsuits against servers of alcohol ('dram-shop laws').<br>- Mandatory seatbelt use law.<br>- Fines for driving under the influence (DUI); e.g., administrative driver's license suspension or revocation.	- None of the beer tax or price coefficients is significantly different from 0.<br>- The magnitudes of the estimated effects are much smaller than those reported in some previous studies.<br>- Seatbelt laws, the minimum legal drinking age, and dram-shop laws typically have statistically significant, negative relationships with fatalities.	- Exactly why the more recent studies do not find significant tax or price effects is not easy to determine. One explanation may be that as other policies have been implemented to deter drunken driving, the marginal effect of taxes and prices has diminished.<br>- This study has found that a number of other public policies besides taxes are significantly related to fatalities, and also that some are not. Our estimates generally indicate that the legal drinking age and laws regarding seatbelts and dram-shop liability are significantly related to fatality rates. No consistent relationships were found between fatalities and a number of mandatory minimum sanctions—administrative per se penalties, DUI fines, and DUI license actions. 'Penalty' variables are less important than factors relating to the probability of arrest. That is, mandatory fines or license suspensions will only be effective if there are substantial efforts to arrest drunken drivers.<br>- Future studies might attempt to control for a number of factors. The 1980s, in particular, was a decade in which awareness of alcohol abuse was on the rise. Surely their impact varied significantly across states and over time. Also, there occurred much tougher and more visible enforcement of existing laws on driving and drinking (and again, this must have occurred differentially across states and over time). Many states also increased drug and alcohol education in the schools. These factors, as well as changes in individual consumption behavior totally unrelated to drunken driving (e.g., the focus on individual health), all were very likely significant factors in the recent declines in alcohol-related fatalities.	**INCLUDE.**<br><br>- Data on fatal traffic accidents were taken from the Fatality Analysis Reporting System (FARS).

[108]	**First author, year, country**	**General aim**	**Study design**	**The policy measure(s) or intervention(s) at hand**	**Measured policy effects on MLDA**	**Reflection on policy and other occurrences by authors**	**Comments**
	Birckmayer, 1999, USA.	The study examined the association between the minimum legal drinking age (MLDA for beer) and suicides among youths ages 18 to 20 years.	- The study used pooled cross-sectional time-series data on youth suicide and the MLDA for the 48 contiguous states in the United States from 1970 to 1990 (using Poisson regression to estimate the association).<br>- Since young people in some states can legally buy alcohol in neighboring states with lower drinking ages, we created an independent variable to capture the potential effect of youths traveling across state borders to obtain alcoholic beverages.	- Data from 48 contiguous states were used between 1970 and 1990.<br>- When MLDAs increased, some state laws included grandfather clauses exempting those who at the time of the effective date had reached the prior legal age for buying alcohol.	A significant association exists between MLDA and youth suicide. Between 1970 and 1990, the suicide rate of 18- to 20-year-old youths living in states with an 18-year MLDA was 8% higher than the suicide rate among 18- to 20-year-old youths in states with a 21-year MLDA.<br>- We found no evidence that potential border crossings from states with higher legal drinking ages to states with lower drinking ages are associated with suicides.<br>- Changes in state-level beer tax were also not associated with youth suicide in our model.	- The potential effect of MLDAs on alcohol consumption is a function not only of drinking age, but also of the level of enforcement of drinking laws. In the case of our 2 crude proxies for enforcement of the MLDA, police per capita and a rolling 5-year average of all-age liquor-law arrests, we found an association for liquor-law arrests only. Liquor-law arrests include a diverse range of violations and do not provide a direct measure of MLDA enforcement.	**INCLUDE.**<br><br>- Data for the primary outcome of the study – the number of suicide victims classified by age and year in each of the 48 contiguous states – came from the Mortality Files of the National Center for Health Statistics.

[109]	**First author, year, country**	**General aim**	**Study design**	**The policy measure(s) or intervention(s) at hand**	**Measured policy effects on MLDA**	**Reflection on policy and other occurrences by authors**	**Comments**
	Chesson, 2000, USA.	This study investigates the effect of alcohol policy on sexually transmitted disease (STD) rates in the US.	Reduced-form regressions of STD rates on state alcohol taxes for the years 1981–95 controlling for state and year are used, as well as quasi- experimental analysis (diagnostic tests and robustness checks) of alcohol policy changes.	Within a 15-year period (1981–95), focus is on MLDA changes and beer taxes (50 states and the District of Columbia) and liquor taxes (only the 32 states, plus the District of Columbia) that do not have state monopolies in wholesale or retail liquor commerce.	-In general, STD rates are responsive to increases in alcohol taxes and in the drinking age. The presumed relationship is that a more restrictive alcohol policy reduces alcohol consumption, which in turn decreases risky sexual activity.<br>- Quasi- experimental analysis of alcohol policy changes support these findings and offers evidence that increases in the drinking age reduce STD rates in the 15- to 19-year old group (sig.), but have no effect on older age groups (the MLDA changes are only significant and negative for ages 15-19).	- It is not surprising that drinking-age changes (which affect only a small subset of the population) do not appear to influence overall STD rates, only for ages 15-19.<br>- The results suggest that drinking-age increases influenced only the age group subject to the drinking-age regulations.	**INCLUDE.**<br><br>- Gonorrhea and syphilis rates (calculated per 100,000 population) are obtained from the Centers for Disease Control and Prevention, which records state surveillance reports of these cases.

[110]	First author, year, country	General aim	Study design	The policy measure(s) or intervention(s) at hand	Measured policy effects on MLDA	Reflection on policy and other occurrences by authors	Comments
	Jones, 1992, USA.	This study examined the effect of legal drinking age (LDA) on fatal injuries (regarding motor vehicle drivers, motorcyclists, pedestrians, unintentional injuries, excluding motor vehicles, suicides and homicides) in persons aged 15 to 24 years in the United States between 1979 and 1984.	Effects on pre-LDA teens, adolescents targeted by LDA, initiation at LDA, and post-LDA drinking experience were assessed using logistic regression analyses.	All 50 states and the District of Columbia were studied for the years 1979 through 1984.	- The results of this study suggest that the net benefit of an LDA of 21 is found not only among motor vehicle drivers, but among other categories of violent death as well (sig. for some).<br>- The findings indicate that raising the LDA may have three effects (sig. for some): 1) delaying legal access to alcohol among pre-LDA adolescents, 2) preventing traumatic deaths that occur with legal access (a 3.9% increase in death rate among persons of a given age who can drink legally) and 3) delaying the onset of heavy drinking and associated fatal injuries that can occur with experience.	-	**INCLUDE.**<br><br>- Mortality data were collected from the National Center for Health Statistics.

[111]	First author, year, country	General aim	Study design	The policy measure(s) or intervention(s) at hand	Measured policy effects on MLDA	Reflection on policy and other occurrences by authors	Comments
	Dee, 2001, USA.	This study provides empirical evidence on the structural relationship between alcohol use and teen childbearing among black and white teens by exploiting the exogenous variation in youth alcohol availability generated by changes in state minimum legal drinking ages.	- Panel data was constructed on childbearing rates by state, year, age, and race for the 1977-92 period.<br>- Reduced-form childbearing models are based on state-level panel data and two-way fixed effect specifications, as well as models that incorporate as controls the contemporaneous childbearing data from older women who were unaffected by the state changes in youth alcohol policy.<br>- An additional regression framework is used similar to 'differences-in-differences-in-differences" (DDD) estimation.	Increases in state-level MLDA over the 1977-92 period.	- The results suggest that teen exposure to a low MLDA (that is, easier access to alcohol) generated statistically significant increases in teen childbearing for both black and white teens.<br>- This conventional panel data model was validated by counterfactual estimations, which found that, in similarly specified models, the movement to higher MLDA had small and statistically imprecise effects on the childbearing rates of older women.<br>- Using DDD estimations, the results suggest that the links between MLDA exposure and teen childbearing are statistically insignificant for white teens, and also indicating that the estimated links between MLDA exposure and teen childbearing persist among black teens. More specifically, the movement to an MLDA of 21 reduced childbearing rates among black teens by roughly 6 percent.	- Effective restrictions on youth alcohol availability and use can lead to sizable reductions in the prevalence of teen childbearing. The persistence of these effects among black teens may also point to the interactive effects of other important contextual factors such as their lower rate of contraceptive use and the relatively small age gap between black teen mothers and the fathers of their children.<br>- This persistent link between alcohol availability and childbearing among black teens is a particularly unusual result since some prior studies have emphasized the unresponsiveness of black teen childbearing to other public policies. Furthermore, these results also provide motivation and guidance for future research on an important determinant of teen childbearing. In particular, the heterogeneity of these results by race suggests provocatively that the alcohol availability of the fathers as well as related sexual behaviors (for example, sexual frequency and contraceptive use) interact to, determine the effects of alcohol availability and use on teen childbearing.	**INCLUDE.**<br><br>- The childbearing data for these evaluations were drawn from various editions of the Vital Statistics of the United States for each state and the District of Columbia.

[112]	First author, year, country	General aim	Study design	The policy measure(s) or intervention(s) at hand	Measured policy effects on MLDA	Reflection on policy and other occurrences by authors	Comments
	Barreca, 2015, USA.	The goal of this paper is to better understand the relationship between MLDAs and birth outcomes in the USA.	From information reported on birth certificates between 1978 and 1988, a 'triple-differences' estimation strategy is employed, which compares birth outcomes across 14- to 20-year-old mothers who were exposed to different MLDAs because of the state and year in which they gave birth. The triple-differences approach also includes a control group of infants born to women who were between the ages of 21 and 24 years.	The analysis mainly focuses on the period when the MLDA was increasing, that is, 1978–1988.	- Our estimates provide limited evidence that MLDA policies affect birth outcomes.<br>- Our analyses of traditional birth outcomes suggest, counter-intuitively, that low MLDAs may lead to better outcomes for Black mothers (versus White mothers) in the most affected age range, and better outcomes among young mothers. As such, much of our evidence points toward positive selection.<br>- Our study also presents novel evidence that an MLDA of 18 years increases fetal losses, as evidenced by the higher probability of giving birth to a female child.<br>- In some cases, we also find evidence that the surviving newborns have better outcomes, which suggests that access to alcohol leads to culling of unhealthier fetuses.	- The year-to-year changes are similar in both the 'treatment' states (states with changes in MLDA) and 'placebo' states (states without changes in MLDA), which suggests that a substantive portion of the time series variation is driven by omitted factors and secular trends unrelated to the changes in the MLDA.<br>- One explanation for the differences (between Black and White woman) is that the policy had differential impacts on drinking behavior across these two racial groups. For example, increases in the MLDA may have reduced binge-drinking among Black mothers relatively more than White mothers. Another possible explanation is that Black women have less health capital and the effects of in utero exposure to alcohol are nonlinear in the maternal level of health capital.<br>- When comparing the 1967-1977 period with the 1977-1988 period (for robustness checks), differences could be expected because of: 1) Changes in women's behavioral responses caused by an increased awareness of the dangers of alcohol consumption during pregnancy, 2) Enforcement of the MLDA may have changed over time.	**INCLUDE.**<br><br>- Our birth outcome data come from the National Center for Health Statistics (NCHS) natality files (1968–1989).<br>- Data on MLDA laws come from the Distilled Spirits Council of the United States.<br>- We have information on the month and year that the MLDAs changed for each state during our sample period.

[113]	**First author, year, country**	**General aim**	**Study design**	**The policy measure(s) or intervention(s) at hand**	**Measured policy effects on MLDA**	**Reflection on policy and other occurrences by authors**	**Comments**
	Grucza, 2012, USA.	The objective of this study is to examine whether individuals who were legally permitted to drink prior to age 21 remained at elevated risk of suicide and homicide in adulthood.	- Analysis of data from the U.S. Multiple Cause of Death files, 1990 to 2004, combined with data on the living population from the U.S. Census and American Community Survey (between 1949 and 1972).<br>- Logistic regression models were used to evaluate whether adults who were legally permitted to drink prior to MLDA 21 were at elevated risk for death by these causes. A quasi-experimental analytical approach was employed, incorporating state and birth-year fixed effects.	The years during which the drinking age was in flux (individuals who turned 18 during the years 1967 to 1990).	- In the population as a whole, we found no association between minimum drinking age and homicide or suicide.<br>- Significant policy-by-sex interactions were observed for both outcomes, such that women exposed to permissive drinking age laws were at higher risk for both suicide and homicide.	It may be that excessive drinking was simply not part of the culture for women born in earlier years of the cohorts examined here and that changing cultural norms may be a key contributor to MLDA-associated suicide and homicide risk observed for more recent birth years. In any event, the fact that MLDA effects were not the same for all birth years indicates that the lower MLDA alone does not result in elevated suicide and homicide risk but that its effects may be dependent on other aspects of the cultural environment.	**INCLUDE.**<br><br>- Data from the U.S. Multiple Cause of Death files.<br>- Data on the living population from the U.S. Census and American Community Survey.

[114]	**First author, year, country**	**General aim**	**Study design**	**The policy measure(s) or intervention(s) at hand**	**Measured policy effects on MLDA**	**Reflection on policy and other occurrences by authors**	**Comments**
	Howland, 1998, USA.	The effects of changes in state MLDA laws on drowning were examined for the targeted (18-20 years) and two adjacent (15-17 years and 21-23 years) age groups, over a period of 21 years (1970-90).	Drowning rates among youth in 48 contiguous US states were examined from 1970 to 1990. Poisson maximum likelihood regression was used to estimate the effects of both lowering and increasing the MLDA.	- Our study examines the effects of changes in the MLDA laws (lowering and raising) from 1970 to 1990.<br>- We use two proxy measures of enforcement: police per capita and all age liquor law arrests.	- No significant association between drowning and MLDA was found for any of the age groups studied.	- It is possible that alcohol's role in drowning is negligible. - It is possible that MLDA laws do not substantially affect drinking in aquatic settings.<br>- A more likely explanation for the failure to find an effect between MLDA laws and adolescent drowning is that during the period of observation other, more powerful factors, were affecting drowning rates. The period observed was marked by a strong secular trend in declining drowning rates. Between 1970 and 1990 the number of drownings for all ages declined by 39%. The reasons for this trend are not apparent.	**INCLUDE.**<br><br>Data on drowning were derived from the National Health Statistics Mortality files.

Fourth path: Secondary societal impact with considering a bridging variable

[35]	First author, year, country	General aim	Study design	The policy measure(s) or intervention(s) at hand	Measured policy effects on MLDA	Reflection on policy and other occurrences by authors	Comments
	Robertson, 1989, USA.	This study examines the amount of alcohol in the blood of fatally injured drivers per licensed driver in states where more than 80 percent of such drivers were tested for alcohol and where licensure data were available for the period 1982 – 1986.	Spill over effects, the legal-age effect and the experience effect were estimated calculating least-squares regression coefficients.	Legal Drinking Age (LDA) of 18 and 21 (in multiple states).	- A higher LDA is associated with fewer alcohol related crashes among those younger that the LDA (not sig.).<br>- The involvement of alcohol in fatal crashes increases with age as the LDA is approached (not sig.).<br>- No effect of drinking experience was evident.<br>- No evidence is found of a short-term spurt effect when teenagers reach the LDA, or of longer-term drinking experience reducing alcohol involvement in fatal crashes.	- Although it is obvious that all teenagers who have not reached the MLDA do not abstain from drinking, the data in this study suggest that a higher MLDA reduces fatal crashes among those below the LDA. The further the driver's age is below the LDA, the lower the alcohol-related crash rates of those drivers.<br>- The correlation among age, alcohol involvement, and night or single vehicle crashes (as is conducted in previous studies) has apparently contributed to an underestimation of the 'spillover' effect of lower LDAs on younger-than-legal-age drivers in previous studies using night and single-vehicle crashes as proxies for alcohol involvement.	**INCLUDE.**<br><br>- Data is used from the National Highway Traffic Safety Administration (FARS data).

[36]	First author, year, country	General aim	Study design	The policy measure(s) or intervention(s) at hand	Measured policy effects on MLDA	Reflection on policy and other occurrences by authors	Comments
	Voas, 2003, USA.	The objective of this research was to determine the extent to which the decline in alcohol-related highway deaths among drivers younger than age 21 years can be attributed to raising the minimum legal drinking age (MLDA) and establishing zero tolerance (0.02% blood alcohol concentration (BAC) limit for drivers younger than age 21 years) laws.	- Data on all drivers younger than age 21 years involved in fatalities in the United States from 1982 to 1997 were used in the study. Quarterly ratios of BAC-positive to BAC-negative drivers in each of the 50 states where analyzed in a pooled cross-sectional time-series analysis.<br>- The ratio of drinking to non-drinking drivers was used to minimize the contribution of state environmental factors, such as urban versus rural composition, the number of licensed drivers in a state, the number of vehicle miles traveled in a state, and changes in vehicle safety (e.g. air bags) and roadway safety features, all of which were expected to influence the total number of fatal crashes, but not to have a differential relationship with the proportion of alcohol-related crashes.<br>- Weighted least squares (WLS) regression models were used, including weighting the cases for some states more than others (data for more populous states are more stable), drinking drivers in fatal crashes become the dependent variable, and alcohol safety laws and other factors known to influence fatal crashes become the independent predictor variables.<br>- Per capita alcohol consumption was, among other factors, used as a control variable; mediation analyses were not performed, neither was the direct effect of MLDA laws on per capita consumption measured.	- Raising the MLDA.<br>- The adoption of zero tolerance (ZT) legislation, making it illegal for drivers younger than age 21 years to drive after drinking alcohol, typically setting the blood alcohol concentration (BAC) limit for youth at 0.00–0.02%.<br><br>- General alcohol safety laws: ALR laws, 0.10 and 0.08 per se limit laws (with a per se level of greater than 0.10% considered the same as absence of a per se law)<br>- Safety belt laws.	- After accounting for differences among the 50 states in various background factors, changes in economic and demographic factors within states over time, and the effects of other related laws, results indicated substantial reductions in alcohol-positive involvement in fatal crashes were associated with the two youth-specific laws (sig.).<br>- The expected levels for a state having both the zero-tolerance law and the MLDA law would be (1–0.189) × (1–0.244) = 0.613 of the nonlaw levels, for a combined reduction of 38.7%.<br>- The effect sizes for the MLDA and zero tolerance laws targeted at this age group are substantial, the zero-tolerance factor appears larger in this analysis.<br>- The adjusted beer consumption measure was, as expected, positively correlated with the impaired driver odds ratio indicating that states with higher consumption levels had more impaired underage drivers.	- Overall, the results of this study suggest that the policy of limiting youth access to alcohol through MLDA laws and reinforcing this action by making it illegal for underage drivers to have any alcohol in their system when driving is reducing the proportion of fatal crashes involving drinking drivers.<br>- The contributions of these individual laws cannot be completely separated. All of these laws are operating in a larger context in which public attitudes towards drinking and driving are influencing both the passage of safety legislation and the occurrence of fatal crashes. Safety laws, in turn, serve to publicize the limits of normative behavior.<br>- The effect sizes undoubtedly reflect influences beyond those resulting from their enforcement. The MLDA laws are only weakly enforced, nevertheless, they apparently have had a significant association with the reduction in alcohol-related fatal crashes involving underage drivers. This suggests that normative changes in drinking and in drinking and driving are occurring that are influenced by the existence of the legislation independently of the strength of its enforcement.<br>- The difference between MLDA and ZT laws may be because two-thirds of the states had already adopted MLDA laws by 1982 when this study began.<br>- The regression analyses conducted in this study is sensitive to specification effects. The national norms regarding drinking and driving may have changed, which is an underlying factor in both the passage of alcohol safety legislation and the number of drinking drivers involved in fatal crashes. There is strong evidence that such a normative change has occurred. This study did not include any measure of media coverage of the drinking-and-driving issue or any measure of public attitudes toward drinking and driving because such a measure was not available.	**INCLUDE.**<br><br>- Information on alcohol-related highway deaths was obtained from the National Highway Traffic Safety Administration's Fatal Accident Reporting System (FARS).

[44]	First author, year, country	General aim	Study design	The policy measure(s) or intervention(s) at hand	Measured policy effects on MLDA	Reflection on policy and other occurrences by authors	Comments
	Hingson, 1983, USA.	- This paper examines the impact of raising the drinking age on teenage drinking, driving after drinking, and non-fatal accident involvement in Massachusetts prior to the law's enactment and twice at yearly intervals after the law enactment.<br>- Massachusetts was compared with New York State, who retained an 18-year-old drinking age.	- Random telephone surveys with approximately 1,000 16-19-year-olds in each state were undertaken prior to the law's enactment and twice at yearly intervals after the law, to assess the law's impact on teenage drinking, driving after drinking, and non-fatal accident involvement.<br>- Respondents were asked where they most often obtained their alcohol (e.g., liquor/grocery store, bar/club, at home) and where they drank > 5 consumptions in the last month (e.g., party, bar).<br>- Drinking was measured asking for ‘any drinking’ in the last month and ‘drinking 6+ drinks at one time’ in the last month.<br>- Log-linear analysis was used on the survey data.<br>- Fatal crash data reported to the US Department of Transportation by each state from 1976-1981 were also analyzed.<br>- To assess law enforcement practices and problems, interviews were conducted with over 50 Massachusetts police officers representing all levels of command in urban, rural, and suburban jurisdictions.	- Legislation raising the legal drinking age in Massachusetts from 18 to 20 in 1979.<br>- At the time Massachusetts raised its legal drinking age from 18 to 20, the two states had similar laws regarding age of driving licensure and penalties for driving while intoxicated.	- After the law, the frequency of teenage drinking in bars and clubs and the percentage of teenagers reporting they most often obtained their alcohol in liquor stores and groceries dropped in Massachusetts compared to New York. (sig.).<br>- During the two years after the law, the proportion of Massachusetts teenagers who had someone else purchase alcohol for them or who most often obtained alcohol from their homes nearly doubled (sig.).<br>- The average daily consumption of alcohol in the 16-19-year-old age group during the two years after the law did not decline in Massachusetts compared to New York. Nor did teenagers report shifts to the use of other psychoactive drugs.<br>- After the law, the proportion of teenagers who reported driving after drinking heavily (six or more drinks) did not decline in either state. However, the frequency that teenagers reported they drove after any drinking declined significantly more in Massachusetts.<br>- The three methods of statistical analysis indicated no significant difference between the two states in the overall teenage fatal accident trends (not sig.).<br>- The frequency with which teenagers were arrested for driving under the influence did not significantly change in Massachusetts during the first year after the law compared to the previous two years.<br>- Enforcement of the law focusing on the sellers was minimal and sporadic.<br>- The intensity of enforcement varied widely from community to community.<br>- The reasons most often cited for the variability in enforcement of the law among communities across the state was the lack of personnel and competing priorities, particularly in some high crime inner-city jurisdictions. Moreover, many officers did not perceive teenage purchasing of alcohol or drinking per se as a sufficiently serious crime to stigmatize<br>Juveniles by putting an arrest on their records.	- The study examined the first two years following enactment of the law. During this time period, the 18 and 19-year-old age groups who had previously been allowed to drink had that privilege revoked. One could hypothesize that the previous drinking habits of this group would be resistant to change.<br>- The state's law provides a symbolic statement to teenagers that its citizens disapprove of their drinking, and fears the accidents they may cause when they drive after drinking. The study results prompt us to ask whether the law could have had a greater impact among all Massachusetts teenagers if enforcement efforts were more consistent and if greater attention were paid to preventing the common practices of non-teenagers purchasing alcohol for teenagers or liquor outlets not requiring age identification. Without sufficient resources and coordination of enforcement efforts, those police who actively strive to enforce the law in one community may find their efforts negated by minimal enforcement in the next.<br>- Lack of community resources and variable willingness to enforce laws focused on teenagers raise questions about whether alternative strategies such as increased enforcement of the drunk driving and traffic safety laws aimed at all drivers, or requirements for safer cars and improved road design would yield greater reductions in nonfatal and fatal accidents both among teenagers and non-teenagers.	**INCLUDE.**<br><br>- In addition to telephone surveys, data from the Fatality Analysis Reporting System (FARS) was used.

[45]	First author, year, country	General aim	Study design	The policy measure(s) or intervention(s) at hand	Measured policy effects on MLDA	Reflection on policy and other occurrences by authors	Comments
	Vingilis, 1981, Canada.	This paper reports four types of data which are relevant to the study of the effects of the increase in the MLDA.	Data are from:<br>1) surveys of drinking and drinking problems among high school students (measuring: 1) alcohol use in the month prior, 2) feeling 'tight' at least once in the month prior, 3) 'drunk' at least once in the month prior, 4) 5 or more drinks on a singes occasion at least once in the month prior). Conducted in 1977 and 1979, stratified proportional sample in the province. Log linear analyses.<br>2) a study (surveys) of perceptions of vice-principals. Conducted in 1972 and 1980.<br>3) a trend analyses of young drinking offender charges; and<br>4) trend analyses of drinking-driving statistics and driver fatalities. Two types of drinking-driving statistics were obtained: monthly Ontario drinking-driving convictions form 1977 and 1979 for 16-21-year-olds and monthly Ontario accident fatalities from 1973 to 1979 for 16-21-year-olds. Time series analyses were used.	The increase of the MLDA in 1979 in Ontario (from 18 to 19).	1) the proportion of drinkers among 18-19-year-olds decreased from 1977 to 1979 (as well as a decrease in the proportion feeling 'tight'). Comparable effects were observed for younger drinkers (under 18) in the exact opposite direction.<br>2) The findings from this study indicate that the vice-principals have perceived either no change or less student drinking and alcohol-related problems at their school since the increase in legal drinking age (between 20 per cent and 30 per cent of the vice-principals reported a decrease in drink-related behaviors).<br>4) There were no significant differences between the pre-intervention and post-intervention time periods for both types of drinking-driving statistics.	- The results of the four studies indicate few statistically significant changes, however, these findings seem to tentatively suggest a minimal effect for 18-19-year-old high school students, but not for the regular (once a week or more) or younger drinkers.<br>- The fact that regular drinkers reported no changes in their drinking habits could partially explain why major changes were not observed for the more general methods of measurement, that is, the rates of accidents, charges and convictions and the perceptions of vice-principals.<br>- Additionally, a large proportion of Ontario youths are not in school and these non-students may be heavier drinkers. Conviction and fatality statistics are not sensitive enough measures, as it seems that too few high-accident-and-arrest-risk youths were changing their drinking patterns for an effect to emerge from these statistics.<br>- The minimal Ontario effect was expected, as it was felt that the one-year increase was not sufficient to cause a major impact on youthful drinking behavior.	INCLUDE.<br><br>- Data from the Fatal Accident Reporting System (FARS).<br><br>- Only 1, 2 and 4 approved criteria and are extracted.

[46]	First author, year, country	General aim	Study design	The policy measure(s) or intervention(s) at hand	Measured policy effects on MLDA	Reflection on policy and other occurrences by authors	Comments
	Fell, 2008, USA.	This study reports on an effort to evaluate and interrelate the existence and strength of two core MLDA laws and fourteen expanded laws designed to (a) control the sales of alcohol, (b) prevent possession and consumption of alcohol, and (c) prevent alcohol impaired driving by youth aged 20 and younger.	- Our first analysis determined if the enactment of the possession and purchase laws (the two core MLDA laws) was associated with a reduction in the ratio of drinking to nondrinking drivers aged 20 and younger who were involved in fatal crashes controlling for as many variables as possible.<br>- Annual FARS data were used from 1982 to 1990. An Analysis of Variance (ANOVA) was used.<br>- Our second analysis determined whether the existence and strength of any of the 16 underage drinking laws was associated with a reduction in the percentage of drivers aged 20 and younger involved in fatal crashes who were drinking. Pooled data from the 1998-2004 FARS data was used. Stepwise Linear Regression models were used.	- Two core MLDA laws (prohibiting possession and purchase of alcohol by youth).<br>- 14 additional underage drinking laws (consumption, furnishing/selling, age 21 for on-premises servers/sellers, age 21 for off-premises servers/sellers, zero tolerance, use and lose, keg registration, RBS training, use of fake ID, transfer/production of fake ID, retailer support provisions for fake ID, social host-underage parties, GDL with night restrictions, state control of alcohol).	- In the presence of numerous covariates, the possession and purchase laws account for an 11.2% ($p = 0.041$) reduction in the ratio of alcohol-positive to alcohol-negative younger than age 21 drivers involved in fatal crashes.<br>- Making it illegal to use a false identification to purchase alcohol was significant (second analysis). Specifically, from state to state, a unit difference (increase) in the strength of the False ID Use law was associated with a 7.3% smaller outcome measure ($p = .034$).	- Differences across states in patterns of underage alcohol use and drinking-related problems may exist that call for varying mixes of legal provisions. Such differences across states in effectiveness of laws could also explain why we found few significant results.<br>- The awareness of these laws by youth and the enforcement of these laws may play a much greater role than the presence, absence or strength (as assessed in this study).<br>- This study did not have the opportunity to uncover the impact of the enforcement of the laws, which may be the most important factor in MLDA effectiveness. Even without substantial enforcement, it may be important for states to adopt effective expanded MLDA 21 laws to have a good foundation in preventing, or at least reducing, underage drinking.	**INCLUDE.**<br><br>- The outcome data - drinking driver and nondrinking driver fatal traffic crashes - were obtained from the Fatality Analysis Reporting System (FARS) maintained by NHTSA.<br>- Information from the National Institute on Alcohol Abuse and Alcoholism (NIAAA) Alcohol Policy Information System (APIS) dataset (1998-2005) was used.<br>- Information from the National Highway Traffic Safety Administration's (NHTSA, 2006) Digest of Impaired Driving and Selected Beverage Control Laws was used.<br>- Information from the Insurance Institute for Highway Safety (IIHS) was used.

[49]	First author, year, country	General aim	Study design	The policy measure(s) or intervention(s) at hand	Measured policy effects on MLDA	Reflection on policy and other occurrences by authors	Comments
	Yu, 1998, USA.	This study aims to examine: (1) the change of alcohol use and purchase patterns among the underaged immediately after the raise of the purchase age; (2) the long-term change in underage alcohol use and purchase patterns after the raise of the purchase age; and (3) the change in impaired driving practices among youth over time after the raise of the purchase age.	- Five telephone surveys were conducted with youths aged 16 to 24 in 10 sampled New York State counties in 1982, 1983, 1985, 1986, and 1996.<br>- Data were collected through telephone interviews from randomly selected New Yorkers (a series of five surveys were conducted in 1982, before the enactment of the 19-drinking age law; 1983, after the enactment of the 19-drinking age law, 1985, before the 21-drinking law; 1986, after the 21-drinking age law, and in 1996, a decade after the 21-purchase age was enacted).<br>- Two dependent variables are included: respondents' frequency of drinking in the past month and frequency of driving under the influence of alcohol in the past month.<br>- A three-stage stratified proportionate random sampling procedure was designed. The 57 non-New York City counties were stratified on the dimension of a county's young adult population (16-20 in 82 and 83, 16-24 in 85 and 86) and a county's alcohol crash involvement among those young adults.	New York state raised the legal alcohol drinking age twice in the 1980's, from 18 to 19 in 1982 and from 19 to 21 in 1985.	- Analysis of the self-reported data showed that, 10 years after the enactment of the 21-drinking age law, alcohol use among 18-, 19-, and 20-year-olds decreased by up to 58% (sig.).<br>- Frequent heavy weekend drinking was reduced by 53% for 16-year-olds and 43% for 17-year-olds between 1982 and 1996 (sig.).<br>- Over the following 10 years, the prevalence of self-reported alcohol use increased slightly to 73%, representing only a 1% decline from the 1985 rate for respondents who were 21 and older.<br>- Alcohol purchase rates of 19- and 20-year-olds were reduced by -70% from 1985 to 1996 (sig.).<br>- From 1982 (before the 19 law) to 1996, the impaired driving rates for 18-year-olds decreased by 84%.<br>- Although impaired driving rates declined over the survey years for each age group, -25% of all underage respondents in 1996 reported that they had ridden in a vehicle with an impaired driver.<br>- Between 1982 and 1983, when the purchase age was raised to 19, the perceived parental approval of alcohol use for the 18-year-old respondents dropped from 69% to 42%. Between 1985 and 1986, when the purchase age was raised to 21, the perceived parental approval rates changed from 68% to 48% for 19-year-olds and from 78% to 56% for 20-year-olds.<br>- In 1996, 69%, 76%, 77%, 82% and 80%, respectively, of the 16-, 17-, 18-, 19-, and 20-year-old respondents thought their peers would approve of their drinking.	- Both the 19 and 21 purchase age laws had immediate impact on alcohol purchase and use by the targeted youth groups. A decade later, the effectiveness of the 21-purchase age law continues.<br>- Analyses of 21-to 24-year-old respondents, who were not affected by the change in the law, showed that alcohol purchase and use rates did not significantly decline from 1985 to 1996. This finding provides further evidence that the purchase age laws tend to have a distinctive impact on the age groups which the laws specifically target.<br>- Weekend drinking tends to be one of the most important factors in youth highway crashes. Findings indicate that weekend drinking and heavy weekend drinking decreased, however, many underage respondents indicated drinking alcohol away from their own home, most commonly at friends' houses. After the establishment of the higher alcohol purchase ages, when youths cannot use alcohol at home in the presence of their parents, their alternative drinking location would be their friends' houses where parents may be absent.<br>- Continued enforcement of the 21-purchase age law will maximize its effect in reducing underage purchase and use of alcohol. Enforcement efforts may emphasize parental involvement in media campaigns.<br>- Anti-drunk driving campaigns, such as "friends don't let friends drive drunk," should not only target adult party-bar goers, but also target underage youths. "Zero Tolerance" laws, which reduce the illegal blood alcohol concentration level to 0.02 for drivers under the age of 21 and which have been enacted in 45 states and the District of Columbia, will also provide additional leverage to enforce the 21-purchase age law.	**INCLUDE.**<br><br>- The New York State Youth Alcohol Survey.<br>- New York State Department of Motor Vehicles records.<br><br>- Follow-up on study [48], using comparable data and methods, only now measuring long-time changes.<br><br>- Unclear about the significance of effects.

[57]	First author, year, country	General aim	Study design	The policy measure(s) or intervention(s) at hand	Measured policy effects on MLDA	Reflection on policy and other occurrences by authors	Comments
	Miron, 2009, USA.	We challenge the view that MLDAs reduce traffic fatalities based on three findings. First, the overall impact estimated in earlier research is driven by states that increased their MLDA prior to any inducement from the federal government. Second, even in early-adopting states, the impact of the MLDA did not persist much past the year of adoption. Third, the MLDA has at most a minor impact on teen drinking.	- We examine the relation between MLDAs and traffic fatalities using aggregate road facilities data of the complete population and of 15-24-year-olds and state-level panel data, reconstructing the analysis of Dee (1999) (using FARS to construct a panel data set for the 48 contiguous states over the period 1977–1992) and extending it to include Alaska, Hawaii, and Washington DC, and the years 1976 and 1993–2005. We focus on 18- to 20-yr-old fatalities.<br>- Using MTF data, we employ the two specific measures common in the literature, ‘‘drinker’’ (having any drink of alcohol in the last month) and ‘‘heavy episodic drinker’’ (having five or more drinks in a row at some point in the last 2 weeks). We also examine the number of motor vehicle accidents that respondents report as occurring after consuming alcohol. We estimate regressions similar to the traffic fatality calculations with these dependent variables. The measure of the MLDA is identical to that used in previous literature, a dummy for having a drinking age of 18 years.	- Changes in de MLDA over the period 1976-2005.<br>- Mandatory seatbelt law.<br>- The BAC limit for legal driving.<br>- Beer taxes.<br><br>We omit several potentially relevant policies, in part because of data availability, in part to conform with Dee (1999), and in part because previous studies have found limited evidence of any impact on TFRs. These variables include dram shop liability laws, mandatory sentences for driving under the influence, sobriety check points, anti-plea-bargaining statutes, changes in tort liability laws that place greater responsibility with intoxicated drivers, happy hour regulations, enforcement (however, enforcement is too low to have any impact on the results examined) and alcohol education programs.	- The MLDA fails to have the fatality-reducing effects that previous articles have reported; trends between 1910 and 2000 in aggregate data of road facility rates show no difference in trends between 15-24-year-olds and that of the entire population.<br>- The declines in the total and 15–24 TFR that began around 1969 long precede the adoptions of an MLDA of 21 in the mid-1980s. The key fact about TFRs, therefore, is that they have been trending downward for decades and have been poorly correlated with the MLDAs.<br>- State-level panel data for the past 30 years show that any nationwide impact of the MLDA is driven by states that increased their MLDA prior to any inducement from the federal government (FUDAA). Even in early-adopting states, the impact of the MLDA did not persist much past the year of adoption.<br>- The MLDA appears to have only a minor impact on teen drinking (sig.), these reductions derive mainly from states that adopted the MLDA21 before enactment of the FUDAA.<br>- Nevertheless, when the number of accidents post-alcohol consumption was analyzed, the panel estimates reveal that movement away from an MLDA of 18 is associated with a statistically insignificant change in reporting of alcohol-related traffic accidents.	- These results suggest that, at most, the MLDA21 reduced TFR18–20 in states that adopted the policy on their own. This raises the question of endogeneity. The MLDA21 in these states may have been enacted in response to grassroots concern against drunk driving or implemented alongside other efforts to reduce traffic fatalities. Relatedly, states that adopted on their own may have been states that devoted significant resources to enforcement.<br>- The limited effects found in non-early adopting additionally challenges the desirability of coercive federalism.<br>- Landmark improvements in the accident avoidance and crash protection features of passenger cars (in place around 1970), and control for advances in medical technology might explain the drastic reductions in traffic fatalities over the past half century and should be made to additionally measure and control for as they are likely to be major determinants of the declining traffic fatality trends.	**INCLUDE.**<br><br>- Data from the Fatal Accident Reporting System (FARS) were used.<br>- Data from Monitoring the Future (MTF) surveys were used.

[59]	First author, year, country	General aim	Study design	The policy measure(s) or intervention(s) at hand	Measured policy effects on MLDA	Reflection on policy and other occurrences by authors	Comments
	O'Malley, 1991, USA.	This purpose of this study; 1) to delineate cross-sectional differences in drinking behaviour among US high school seniors and young adults that may be due to variations in recent years in state-level MLDA laws, and 2) to examine the effects of recent changes in MLDA on alcohol consumption and other relevant attitudes and behaviours.	- Analyses used existing data collected by the MTF project, a separate coordinated study used time-series analyses of official reports to examine effects of increases in the MLDA in several states on rates of fatal crashes.<br>- The consumption of alcohol is measured by asking respondents for the number of drinking occasions during the last 30 days and the last two weeks (having five or more drinks in a row).<br>- Time-series results were compared with findings from self-reported data.	- Differing MLDA between 1976 and 1987.	- Higher MLDA were associated with lower levels of alcohol use among high school seniors and recent high school graduates (even after multivariate controls, MLDA remains a significant and substantive predictor of alcohol use).<br>- Combined across all states that increased the MLDA from 18 (to 19, 20 or 21), there was a 13.3% decrease in drinking in the past 30 days.<br>- Alcohol involved highway crashes decline among the 18- to 20-year-old population (sig.), and the present research makes it clear that the decline is directly a result of lower levels of consumption (and because the under-21 group spend less time in bars and taverns when the MLDA is 21).<br>- The lower levels of use persisted into the early 20s, even after all respondents were of legal age and the alcohol is legally accessible.	- Time trends in alcohol use in the constant-21 states are not monotonic. This shows that overall declines in alcohol use were not attributable solely to changes in MLDA. However, some declines appear due to the effect of changes in the laws, because the states that increased their MLDA showed larger declines.<br>- The most common alternative explanation for differences in drinking behavior by high school seniors associated with different MLDA, is that states also differ on other factors, such as standards of religion, anti-alcohol attitudes, or whether states changed their laws voluntarily, or in response to external forces (federal action).	**INCLUDE.**<br><br>- Data from Monitoring the Future (MTF) surveys were used.<br>- Data from the Fatal Accident Reporting System (FARS) were used.

[62]	First author, year, country	General aim	Study design	The policy measure(s) or intervention(s) at hand	Measured policy effects on MLDA	Reflection on policy and other occurrences by authors	Comments
	Smith, 1984, USA.	The effects on the raised MLDA in Massachusetts is examined on drinking, drinking and driving, and nonfatal and fatal crash involvement of 16-17-year-olds (teenagers immediately younger than those targeted by the law).	- Data from Massachusetts are compared with those from New York, where the MLDA remained at 18.<br>- A total of 3 years of survey data from the two states and 6 years of data from FARS were used for pre- and post-law comparisons.<br>- An anonymous random digit-dialing telephone survey was conducted in Massachusetts prior to enactment of the law in 1979, asking teenagers about personal characteristics, drinking practices, procurement of alcohol, use of psychoactive drugs, driving after drinking and nonfatal accident involvement. A similar survey was conducted in the New York area (respondents were asked for their drinking behavior and behavior on how they obtained their alcohol comparable to the New York sample).<br>- Twice at yearly intervals following enactment of the law, surveys of similar size were repeated in each state.<br>- Log-linear analysis was used on the survey data.<br>- In addition, FARS data was used for both states from 1976 to 1982 (single-vehicle nighttime accidents were examined separately).<br>- Data were fitted to a log-linear model, an analysis of variance and an analysis of covariance.	The 1979 Massachusetts law raising the MLDA from 18 to 20.	- The findings suggest that raising the MLDA had minimal effects on the drinking behavior of Massachusetts teenagers.<br>- There was a significant decline in Massachusetts in the number of teenagers reporting drinking at a bar/club/restaurant or in an automobile, an increase of teenagers drinking at parties and a decrease in the number of teenagers purchasing alcohol at liquor stores after the raise of the MLDA and compared to New York.<br>- Massachusetts 16-17-year-olds were more likely in each of the 2 post-law years to have had others purchasing alcohol for them (compared to New York).<br>- After the enactment of the law, driving after drinking declined significantly in Massachusetts relative to New York.<br>- Our analysis did not reveal a significant difference in single-vehicle nighttime fatal accidents (and total fatal accidents) among 16-17-year-olds in Massachusetts and New York after the enactment of the law.	- The present findings suggest that the effect of the MLDA changed where these teenagers drink and how they obtain alcohol.<br>- It is interesting that both before and after enactment of the law, 16-17-year-olds in New York drove less frequently after drinking compared to Massachusetts teenagers. Perhaps, this is the result of having a stable (but lower) drinking age in New York over several decades.<br>- Changes in drinking age may offer some reduction in teenage traffic crash involvement, but teenage drinking and teenage driving after drinking remain serious problems, even in states that raise their MLDA.<br>- The present study indicates lawbreaking among young people, perhaps fostering cynicism toward the legislative process and disregard for law enforcement. As long as teenagers are never asked for ID or have others purchase alcohol for them, significant declines in harm are unlikely.	**INCLUDE.**<br><br>- Data from the Fatal Accident Reporting System (FARS).<br><br>- Hingson et al. (1983) examined the first 2 post-law years in Massachusetts [44].

[64]	First author, year, country	General aim	Study design	The policy measure(s) or intervention(s) at hand	Measured policy effects on MLDA	Reflection on policy and other occurrences by authors	Comments
	Buijs (chapter 4, in Van Havere), 2017, Belgium.	The goal of the current study is to investigate whether the legislative change of the minimum legal drinking age in Belgium, after 2009, has had an impact on health. Considering the fact that this change is fairly recent, it should be clear that the current research has to be restricted to the direct, short-term impact on adolescents, since any potential long-term changes in adult chronic conditions will only become tangible in the years to come. As such, this study focuses on the impact of the minimum age on adolescent injuries and neuropsychiatric conditions linked to alcohol.	- Data was collected on the incidence of hospital-based health service use between 2002 and 2013, linked to the diagnostic codes using databases from the Belgian Federal Public Service (FPS) (two main, administrative registration systems recording the incidence of hospital-based healthcare use (MZG/RHM & MPG/RPM)). Data from both registration systems was collected, based on diagnostic codes, sex and age groups. Adolescents were grouped into three age categories (12-15-, 16-17- and 18-19 years).<br>- Descriptive analyses are provided, using sex- and age-standardized incidence rates per 10,000 population. Single-level, negative binomial models were applied in which the relationship between the independent covariates age group, time and gender and the dependent variable, incidence rate, was investigated. An interaction term between age group and time was added to see whether certain age groups had a higher or lower incidence risk after the implementation of the legislation.	- The impact of the minimum legal drinking age in Belgium. - In December 2009, the Belgian government amended its existing alcohol legislation, to prohibit access to distilled alcoholic beverages to adolescents under the age of 18 and access to all (both distilled and fermented) alcoholic beverages to adolescents under the age of 16. This restriction applied to both the possession by adolescents, as well as alcohol sales by retailers, bars and restaurants.	- In general, the results of the statistical analysis did not provide evidence that the legislative change of the minimum legal drinking age in Belgium, after 2009, has had an impact on health-related outcomes (i.e. neuropsychiatric and acute conditions).<br>- The decreasing trend that was found in adolescent alcohol consumption (chapter 3), was not found in health-related outcomes. Instead, diverging time trends were found depending on the health-related outcome.	- These health-related outcomes showed increasing, decreasing or stable prevalence over time, but no significant trend reversal could be observed after 2009 indicating that the minimum legal drinking age in Belgium did not have an impact on health-related outcomes.<br>- The absence of time trends in problematic drinkers could be expected since this is another target group than the one on which the 2009 minimum legal drinking age is targeting on.	**INCLUDE.**<br><br>- The first one is called 'Minimum Hospital Data' ('Minimale Ziekenhuisgegevens' [MZG]/ 'Résumé Hospitalier Minimal' [RHM]).<br>The second registration system is called 'Minimum Psychiatric Data' ('Minimale Psychiatrische Gegevens' [MPG]/'Résumé Psychiatrique Minimum' [RPM])

[75]	First author, year, country	General aim	Study design	The policy measure(s) or intervention(s) at hand	Measured policy effects on MLDA	Reflection on policy and other occurrences by authors	Comments
	Lillis, 1987, USA.	Assessing the impact of increasing the legal drinking age on drinking and driving behavior.	- A multiple-indicator, pre-comparison and post-comparison design was used.<br>- Changes in indicators for 18-year-olds were compared with changes for other age groups.<br>- Three independent data sources related to drinking and driving were utilized: 1) the accident records submitted by police agencies to the Department of Motor Vehicles (data from two periods was used; 12/4/81 to 12/3/82 and 12/4/82 to 12/3/83), 2) arrest records maintained by the New York State Police, and 3) self-reported drinking-and-driving data (measuring 'driving after feeling the effects of alcohol' at least once in the 28 days prior to the survey), and questions regarding the purchase behavior of specific beverages (i.e., beer, wine or liquor) in the preceding 28 days at specific locations (i.e., bars of stores)) culled from the Youth Alcohol Survey (using telephone interviews comprising 2000 16- to 20-year-old New Yorkers conducted in 1982 (before the increase) and 1983 (after the increase)).	The legal minimum purchase age for alcohol beverages in New York State that increased from 18 to 19 years old in 1982.	- Analyses of three independent indicators of drinking and driving provide strong support for the hypothesis that the increase in the legal purchase age in New York State from 18 to 19 years resulted in decreases in drinking-and-driving behavior by 18-year-olds.<br>- Changes in the rates of drinking-and-driving indicators showed a 21% decrease in fatal and injury-causing crashes and a 39% decrease in fatal crashes for 18-year-olds (sig.).<br>- Non-crash DWI arrests of 18-year-old drivers decreased 35% (sig.).<br>- A 46% decrease in self-reported drinking and driving from the survey were found for 18-year-olds (sig.) following the increase in the purchase age to 19.<br>- For all measures, the rate of decrease for 18-year-olds was significantly greater than the rate of decrease for drivers 21 years old and older.<br>- After the increase, the rate of beer purchasing by 18-year-olds was significantly smaller than the rate for 19- or 20-year-olds (33% vs. 51% and 47%, respectively), and decreased significantly for 18-year-olds following the increase (from 52% to 33%, respectively).	- The findings support the theory that the 19-year-old purchase age in New York has had a major immediate impact on 18-year-olds in the state.<br>- Opponents of purchase-age increases have argued that any changes in drinking-driving crash rates are due to the STOP-DWI legislation and programs, and not to purchase age policies. However, none of the laws and few programs have been aimed specifically at young drivers (e.g., 18-year-olds). Efforts of STOP-DWI cannot explain the differences between the rate of change for 18-year-olds.	INCLUDE.<br><br>- The accident records submitted by police agencies to the Department of Motor Vehicles.<br>- Arrest records maintained by the New York State Police.<br>- The Youth Alcohol Survey.

[80]	First author, year, country	General aim	Study design	The policy measure(s) or intervention(s) at hand	Measured policy effects on MLDA	Reflection on policy and other occurrences by authors	Comments
	Dee, 2003, USA.	This study examines the effects of teen alcohol use and availability on educational attainment, demonstrating effects of changes in MLDA on drinking and educational attainment.	- The first section of this article uses the NELS-88 to establish an empirical baseline: teens who drink (measured as past month alcohol use called 'drinkers', drinking 10 or more drinks within the past month called 'moderate drinkers, and the consumption of five or more drinks in a row the last two weeks is called 'heavy drinker') are less likely to complete high school and less likely to enter college using linear probability estimates / weighted OLS regressions.<br>- Increases in MLDA during the 1980s are used as exogenous determinants of teen drinking (using data from MTF-surveys in Weighted Least Squares Estimates).<br>- The last section tests whether educational attainment within states increased after the MLDAs were increased, using PUMS data and Reduced-Form and Two-Sample Instrumental Variables Estimates.	This study used the increases in MLDA during the 1980s and state excise taxes on beer.	- Teens who drink are less likely to complete high school and less likely to enter college.<br>- We demonstrate that teens who faced an MLDA of 18 were substantially more likely to drink than teens who faced a higher drinking age.<br>- Teen exposure to an MLDA of 18 had small and statistically insignificant effects on indicators for high school completion, college entrance, and college persistence.	- Estimates of the policy determinants of teen drinking demonstrated that, though the cross-state variation in beer taxes correlates with teen drinking, the within-state variation does not. Therefore, frequent recommendations for increased beer taxes appear to be based on what may only be a spurious correlation generated by unobserved state heterogeneity.<br>- The within-state increases in MLDA, which significantly affected all levels of teen drinking, provided a source of exogenous variation for identifying the true effect of teen alcohol consumption on educational attainment.<br>- By focusing on the magnitudes of the links among alcohol policy, teen drinking, and educational attainment, this identification strategy has also underscored the fact that alcohol control policies could at best be a fairly weak policy lever for improving the levels of schooling among youth.<br>- Other policy interventions with larger and more direct links to the schooling decisions made by teens should be able to promote a greater improvement in the accumulation of human capital.	**INCLUDE.**<br><br>- National Education Longitudinal Study of 1988 (NELS-88)<br>- Data from the 1977–92 Monitoring the Future (MTF) surveys<br>- Data from the 1960–69 birth cohorts in the Census Bureau's 1990 5% Public-Use Microdata Sample (PUMS).

[82]	**First author, year, country**	**General aim**	**Study design**	**The policy measure(s) or intervention(s) at hand**	**Measured policy effects on MLDA**	**Reflection on policy and other occurrences by authors**	**Comments**
	Decker, 1988, USA.	- It is essential to know whether raising the MLDA to 21 can materially reduce the number of deaths among persons aged 15 through 24 years caused by motor vehicle crashes (MVCs).	- Analyzed the Tennessee MVC data (FARS) for 1980 through 1986. Additionally, a count of licensed drivers by county, population and deaths counts by county, an estimate of total miles driven in Tennessee, an estimate of the total and nighttime miles driven and estimates of the prevalence of self-reported DUI were used.<br>- Three age groups were considered; those aged 15 through 18 years, 19 through 20 years and those aged 21 through 24 years.<br>- Focus was on single-vehicle nighttime (SVN) fatal crashes to obtain a sensitive measure for the extent of alcohol involvement in MVCs.<br>- Rates within a given age stratums were compared by using the incidence density ration method, to compare rates among age groups, two-tailed t-tests were performed.	- In 1984, Tennessee raised its MLDA for possession or purchase of alcohol from 19 to 21 years (the age-law).<br>- In 1982, Tennessee adopted legislation that markedly increased the likelihood of imposition and the severity of penalties for conviction for DUI (the penalty-law).	*(only focusing on effects subsequent to the age law)*<br>- There was a significant and persistent decline (38%) in the *SVN fatality rate* among drivers aged 19 through 20 years following implementation of the age-law (no border effects were found).<br>- Among persons aged 15 through 18 years, in 1985, the year following the introduction of the age-law, the *overall* MVC death rate dropped 24% (sig.).<br>- Similarly, in 1985, the year following introduction of the age law, the *overall* MVC death rate dropped 24% (sig.) among persons aged 19 through 20 years.<br>- During 1980 through 1982, the *crude death rate* for persons aged 15 through 18 years declined 13% for 1883 through 1986 (sig.).<br>- *The mean BAC* in 1985 for youth 15 through 18 years was 48% lower than all other years in the study period (sig.). After implementation of the age-law, the mean BAC for 19 through 20-year-olds showed a 35% reduction (sig.) and 20% (sig.) for drivers aged 21 through 24 years.	- The implementation of a law denying alcohol to persons aged 19 through 20 years caused a sudden and dramatic decline in drunk driving among that age group, an effect still present at the end of the study period.<br>- Stiffened penalties or increased enforcement directed against DUI has been associated with declines in DUI fatality rates, however, beneficial effects began before the legal changes, parallel with increasing publicity and social disapproval stimulating those legal changes, and beneficial effects almost invariably disappeared within a few years.<br>- Our data suggest that publicity and other social influences may have played a particularly important role in producing the prolonged reduction in alcohol related MVC mortality seen in the 15- through 18-year-old age group.<br>- Our data indicate that laws raising the MLDA to 21 can be highly effective in reducing alcohol-related MVCs among drivers aged 19 through 20 years, a group apparently quite resistant to the effects of increased DUI penalties end anti-DUI publicity.<br>- We cannot clearly apportion the responsibility for the benefits among influences link DUI laws or a new MLDA, but each appears contributory.	**INCLUDE.**<br><br>- Data on fatal traffic accidents were taken from the Fatality Analysis Reporting System (FARS).

[100]	First author, year, country	General aim	Study design	The policy measure(s) or intervention(s) at hand	Measured policy effects on MLDA	Reflection on policy and other occurrences by authors	Comments
	Filkins, 1981, USA.	- Twelve years of Michigan accident data, assess the impact of lowering the minimum legal drinking age from 21 to 18 in 1972, and subsequently raising it back to age 21 in 1978.<br>- DUIL (Driving Under the Influence of Liquor) arrest data were also obtained and analyzed for 1978 and 1979.	- All accidents occurring in Michigan that were investigated by police agencies and reported on official forms were obtained for the twelve years from 1968 through 1979.<br>- Driver age and the presence or absence of drinking in the accident were the key variables, and partitioning the chi-square statistic into its degrees of freedom was the primary analytic technique.	Lowering the minimum legal drinking age from 21 to 18 in 1972, and subsequently raising it back to age 21 in 1978, and DUIL arrest data (for 1978 and 1979).	- The results show clearly that the minimum legal drinking age influences drinking-driving patterns among the affected age groups. Alcohol related accidents increased among 18- to 20-year-old drivers when the legal drinking age was reduced to 18, and non-fatal accidents decreased when it was later increased to 21 (sig.).<br>- Analysis of the DUIL arrest data also demonstrates clearly that the recently increased legal drinking age altered drinking-driving practices among the affected drivers (not sig.). The 18-, 19-, and 20-year-old drivers, whether considered singly or as a group, consistently showed fewer arrests in 1979 than in 1978.	In addition, there have been changes in the larger context which influence drinking and driving patterns, both singly and in combination. One can cite the energy crisis of the mid-1970's and the recent economic downturn. These perturbations, of course, preclude simple before-after comparisons of only the affected age groups in the analytical and inferential work, but they cannot in any sense be used to dismiss the findings out of hand.	**INCLUDE.**<br><br>- Michigan accident data.<br>- Arrest data for DUIL.

[115]	First author, year, country	General aim	Study design	The policy measure(s) or intervention(s) at hand	Measured policy effects on MLDA	Reflection on policy and other occurrences by authors	Comments
	Fertig, 2009, USA.	This study examines the consequences of minimum legal drinking age (MLDA) laws on birth outcomes.	- To estimate the effect of MLDA laws on birth outcomes, we use a linear probability model which includes year–month of conception fixed effects, state fixed effects, maternal age at conception fixed effects, and state-specific time trends.<br>- Women aged 21–24 at the time of conception are treated as a control group to account for unobserved time-varying state-level factors which could affect infant health.	Increases in state minimum drinking ages over the 1980s (using birth years 1978–1989 to create a sample of births conceived in the years 1978–1988 to mothers aged 14–24 at the time of conception).	- Changes in the minimum legal drinking age (MLDA) are related to prenatal drinking when the drinking age is 18 (sig.).<br>- A drinking age of 18 is associated with adverse outcomes among births to young mothers (particularly strong for black mothers), including higher incidences of low birth weight (sig.) and premature birth (sig.), but not congenital anomalies.<br>- Parental characteristics are related to MLDA laws, a lower drinking age is associated with lower educational levels for white women (sig.) and the absence of paternal information on the birth certificate for black women (sig.).	- Alcohol policy that more effectively curtailed drinking, or the risky behaviors associated with it, might hold greater promise for infant health.<br>- The evidence suggests that lenient drinking laws generate poor birth outcomes in part because they increase the number of unplanned pregnancies.<br>- Our results also suggest that stricter alcohol policies may have positive unintended consequences—benefits for the well-being of a generation beyond those directly targeted.	**INCLUDE.**<br><br>- Data from the National Longitudinal Survey of Youth (NLSY) is used.<br>- Data is used from the National Vital Statistics (NVS) for the years 1978–1988.<br>- Data on MLDA laws come from the Distilled Spirits Council of the U.S. (DISCUS)

[116]	First author, year, country	General aim	Study design	The policy measure(s) or intervention(s) at hand	Measured policy effects on MLDA	Reflection on policy and other occurrences by authors	Comments
	Joksch, 1993, USA.	This study examined whether limiting legal access to alcohol for certain age groups affected the commission of selected crimes by individuals in those age groups.	- Arrest data from the Uniform Crime Reports and FARS-data were analyzed in relation to changes in the drinking age in the range 18 to 21 years.<br>- The analysis had three parts: 1) relating blood alcohol of drivers in fatal accidents to changes in the drinking age, 2) relating arrests to changes in the drinking age and 3) relating changes in blood alcohol to changes in arrests.	Changes in the drinking age in the range 18 to 21 years (only states that raised the drinking age between 1981 and 1986) were studied (18 states), in addition, comparison states with no changes in their drinking ages were used (13 states).	- Reduced alcohol involvement in fatal accidents was, overall, significantly related to a raised drinking age.<br>- The corresponding analyses of arrest data showed declines on the order of 10 per-<br>cent related to raised drinking ages for vandalism and for disorderly conduct, but not for violent crimes.<br>- This study found a strong suggestion that among the four crime types, the effect of the raised drinking age increased with decreasing severity of the crime.<br>- This study found no indication of the expected relationship between changes in the drinking indicator and changes in the crime indicators (however, standard errors were very large).	The effects of raising the drinking age may differ among the states due to differences in the distribution systems for alcoholic beverages, varying degrees of enforcement, neigh-boring states with lower drinking ages, and more subtle socioeconomic factors.	**INCLUDE.**<br><br>- Arrest data from the Uniform Crime Reports were examined.<br><br>- Fatal (motor vehicle) Accident Reporting System (FARS) was studied.

[117]	First author, year, country	General aim	Study design	The policy measure(s) or intervention(s) at hand	Measured policy effects on MLDA	Reflection on policy and other occurrences by authors	Comments
	Norberg, 2009, USA.	We use a ‘‘natural experiment’’ study design to compare the 12-month prevalence of alcohol and substance use disorders among adult subjects exposed to different minimum legal drinking age laws in the 1970s and 1980s.	- The sample pools 33,869 respondents born in the United States from 1948 to 1970, and drawn from 2 nationally representative cross-sectional surveys. Analyses control for state and birth year fixed effects, age at assessment, alcohol taxes, and other demographic and social background factors.<br>- The main outcome measures for the present study were binary variables reflecting whether the respondent met DSM-IV criteria for alcohol, marijuana, or other illegal substance abuse or dependence within the previous 12 months.<br>- Logistic and probit regression models were used to estimate the relative odds or relative risk of a past-year alcohol or drug use disorder or cross-state migration among ‘‘exposed’’ and ‘‘unexposed’’ subjects, and to investigate the possibility of differences in effect estimates across demographic groups.	- Changes in minimum legal drinking age (MLDA) laws in the United States during the 1970s and 1980s.<br>- Control for state beer taxes in the year that the respondent turned 18.	- Adults who had been legally allowed to purchase alcohol before age 21 were more likely to meet criteria for an alcohol use disorder or another drug use disorder within the past year, even among subjects in their 40s and 50s (exposure to a younger legal purchase age is associated with more than a 30% increase in the odds of a past-year alcohol use disorder) (sig.).<br>- The effect estimates were little changed by inclusion of age of initiation as a potential mediating variable in the multiple regression models.<br>- There were no significant differences in effect estimates by respondent gender, black or Hispanic ethnicity, age, birth cohort, or self-reported age of initiation of regular drinking.	- The MLDA effects do not seem to be working through age of drinking initiation: although MLDA exposures did predict age of onset of regular and weekly drinking, the effect estimates for alcohol and substance use disorders were little changed in regression models that also included lifetime abstention status and age of initiation, and were similar and separately significant among subjects who had already started to drink by the age of 16 and among those who had not. Although age of onset may indeed be a causal risk factor for later substance use disorders, these results suggest that the long-term effects of MLDA exposures on harmful drinking may work through other aspects of late adolescent drinking, such as the intensity or patterning of drinking.<br>- It is plausible that the effects of MLDA laws on patterns of drinking among early onset drinkers could be explained by the influence of MLDA laws on the behavior of their more law-abiding peers. For example, if young adults prefer to drink with friends than to drink alone, then even among respondents who had already begun to drink before age 18, a more restrictive purchase age could influence the frequency, activities, and social composition of the encounters around which people form enduring social relationships.<br>- MLDA laws were not the only social processes that might have affected lifetime patterns of alcohol and other substance use for cohorts coming of age in this period. If the apparent MLDA effects are not attributable to changing drinking age laws themselves, then they are due to an environmental factor closely tied to the timing of these changing laws.<br>- We have not tried to account for cross-state differences in law enforcement or ease of access to alcohol in neighboring states, and we did not distinguish between furnishing, purchase, possession, and consumption.	**INCLUDE.**<br><br>- The 1991 National Longitudinal Alcohol Epidemiological Survey (NLAES)<br>- The 2001 National Epidemiological Study of Alcohol and Related Conditions.

[118]	First author, year, country	General aim	Study design	The policy measure(s) or intervention(s) at hand	Measured policy effects on MLDA	Reflection on policy and other occurrences by authors	Comments
	Plunk, 2015, USA.	We used MLDA changes during the 1970s and 1980s as a natural experiment to investigate how underage exposure to permissive MLDA affected high school dropout.	- MLDA exposure was added to two data sets: (a) the 5% public use microdata samples of the 1990 and 2000 censuses (n = 3,671,075), and (b) a combined data set based on the 1991–1992 National Longitudinal Alcohol Epidemiological Survey (NLAES) and the 2001–2002 National Epidemiological Survey on Alcohol and Related Conditions (NESARC; n = 16,331).<br>- We used logistic regression to model different thresholds of MLDA on high school dropout. We also estimated models conditioned on demographic variables and familial risk of developing alcohol problems.	The period of greatest change in MLDA (1978–1987).	- Only the MLDA of 18 predicted high school dropouts (sig.).<br>- Exposure was associated with 4% and 13% higher odds of high school dropout for the census and NLAES/NESARC samples, respectively.<br>- The MLDA of 18 also promoted transitioning from regular to weekly drinking while of high school age, but only for those individuals with a history of parental alcohol problems (sig.).	- The most plausible way by which underage high school students were affected by the MLDA of 18 would be their 18-year-old peers, which suggests that permissive MLDA could have promoted high school environments similar to those we observe on college campuses today. Current proposals to lower the drinking age in response to risky underage college drinking would need to address the degree to which these behaviors would occur earlier at the high school level. The apparent differential effect based on predisposition for developing drinking problems also provides further evidence that policy can successfully affect drinking behavior in young adult populations characterized by high environmental and genetic risk.<br>- High school dropout is a complex phenomenon, and the data sets we used did not capture many relevant risk factors (e.g., individual-level factors including parental educational attainment, childhood experiences, and socioeconomic status; school- or district-level factors such as quality of instruction and local funding). As such, our estimates reflected the average effect of MLDA exposure while holding constant other unmeasured factors that might also have influenced educational attainment.	**INCLUDE.**<br><br>- The Integrated Public Use Microdata Series for the 1990 and 2000 decennial censuses.<br>- The 1991–1992 National Longitudinal Alcohol Epidemiological Survey (NLAES).<br>- The 2001–2002 National Epidemiological Survey on Alcohol and Related Conditions (NESARC).

[119]	**First author, year, country**	**General aim**	**Study design**	**The policy measure(s) or intervention(s) at hand**	**Measured policy effects on MLDA**	**Reflection on policy and other occurrences by authors**	**Comments**
	Yu, 1995, USA.	This paper examines the impact of the purchase age law on the involvement in drinking-driving convictions by age groups and birth cohorts in the 1980's in New York.	A systematic random sample was selected from the New York state driver license file, which contains 15,032 drivers who were convicted of DD (driving while intoxicated/driving while ability impaired) at least once in the period from 1978 to 1988.	New York state raised the legal alcohol drinking age twice in the 1980's, from 18 to 19 in 1982 and from 19 to 21 in 1985.	- The involvement of the 16-20 age group in DD convictions decreased in the late 1980s. The proportion of the 16-20 group in the convicted DD population decreased from 15% in 1980 to 7.6% in 1988, a reduction of 50%.<br>- Accompanying this decrease, is a shift of the modal age of the convicted population each year. In 1980, 20.7% of the convicted offenders population was 21-24, in 1988 the model age moved to 25-29 (independent of the driving population and only evident in the analyses of the 21 MLDA).<br>- A pattern suggests that at the same age, the early birth cohorts were more involved in DD convictions than the later cohorts.	- This study supports the restriction hypothesis and the beginning drinker hypotheses. DD involvement tends to be influenced by a cohort effect. Proportions in DD convictions increase when adults become beginning drinkers, when they become serial beginning drinkers their DD involvement remains high over time. On the other hand, the general trend indicates that DD convictions of the affected age groups are on the decline.<br>- Continued efforts should be made to enforce the 21 MLDA, since it appears to be effective in reducing involvement in DD among affected age groups.<br>- Special education programs should be mounted for youths approaching the MLDA to prepare them for transition.	**INCLUDE.**<br><br>- The New York state driver license file.<br><br>- Statistical significance is not displayed or calculated.

[120]	**First author, year, country**	**General aim**	**Study design**	**The policy measure(s) or intervention(s) at hand**	**Measured policy effects on MLDA**	**Reflection on policy and other occurrences by authors**	**Comments**
	Chaloupka, 1991, USA.	The purpose of this study is to estimate the effects of drunk driving deterrents and other alcohol related policies on drunk driving.	- The dataset employed is an annual time-series of state cross-sections for the 48 contiguous states of the U.S. from 1982 through 1988.<br>- Total and alterative alcohol involved motor vehicle fatality rates, for the general population and for 18-to-20-year-olds are used as measures of drunk driving.	- The dataset covers the years from 1982 through 1988 for the 48 contiguous states of the US.<br>- All the important DUI legislation (implied consent laws, illegal and administrative per se laws, minimum legal drinking ages (including controls for grandfather clauses and border crossing), preliminary breath test laws, open container laws, no plea-bargaining laws, laws specifying mandatory minimum penalties for conviction of driving under the influence and dram shop statutes of case laws) are estimated simultaneously.	- The results indicate that the most effective policies are increased beer taxes and mandatory administrative license action of one year.<br>- The next most effective policies are the 21-year-old legal drinking age, preliminary breath test, dram shop laws and relatively large mandatory fines.	-	**INCLUDE.**<br>- Information from the National Highway Traffic Safety Administration (NHTSA) Fatal Accident Reporting System (FARS) were used to measure drunk driving.

[121]	**First author, year, country**	**General aim**	**Study design**	**The policy measure(s) or intervention(s) at hand**	**Measured policy effects on MLDA**	**Reflection on policy and other occurrences by authors**	**Comments**
	Houston, 1995, USA.	This study assesses the effectiveness of state regulatory efforts to improve traffic safety.	Annual data for the period 1967 to 1991 for all 50 states were analyzed through estimates in a constant coefficient model for a pooled time series analysis.	Prominent state actions in this policy area (traffic safety) include legislation mandating the use of seat belts and raising the minimum legal drinking age. Changes to the speed limits on state highways also must be considered in any discussion of traffic safety.	- Mandatory seat belt laws, an increased minimum legal drinking age and a 55-mph maximum speed limit were all found to significantly reduce state traffic fatality rates.<br>- The state MLDA is highly significant, suggesting that a change in the MLDA has a persistent effect on state fatality rates. - The implementation of mandatory seat belt laws also has a statistically significant influence in reducing the state fatality rate (regardless of the method of enforcement).	- This analysis does not consider the impact of drinking-and-driving laws. Future studies of this nature should incorporate these policies designed to deter alcohol-impaired driving to better assess the true nature of all state regulatory policies designed to reduce motor vehicle fatalities.	**INCLUDE.**<br><br>- Data from the Fatal Accident Reporting System (FARS) was used.<br>- Data from the annual report 'Accident Facts' were used.

[122]	First author, year, country	General aim	Study design	The policy measure(s) or intervention(s) at hand	Measured policy effects on MLDA	Reflection on policy and other occurrences by authors	Comments
	Dang, 2008, USA.	The percent of drivers involved in fatal crashes who had consumed alcohol and had blood alcohol concentration (BAC) of .08 or above prior to the crash steadily decreased from 1982 to 1997 and then leveled off (more or less). In an attempt to explain the 1982-1997 reduction and the 1997-2005 level trend, this report presents a statistical analysis of factors that influenced the historical alcohol-related driving trends from 1982 to 2005.	The study is based on disaggregate logistic regression of imputed Fatality Analysis Reporting System (FARS) cases from all 50 States and the District of Columbia from 1982 to 1997, to (1) provide clarification to the change in the historical alcohol-related driving trends and (2) assess the effects of alcohol programs that directly and indirectly changed public attitudes and behavior toward drinking and driving during the last 20 years.	Alcohol-related legislation that are independent variables:<br>1) .10 BAC laws make it illegal to operate a motor vehicle at or above .10 g/dL. Most of the States passed this law during the 1980s. All 50 had passed .10 laws by 1997.<br>2) .08 BAC laws make it illegal to operate a motor vehicle at or above .08 g/dL. By 1997, 16 out of 51 States had enacted this law, and by 2005 every State had.<br>3) Administrative License Revocation (ALR) laws revoke or suspend the driver's license of an individual who refuses to submit to a BAC test or fails a test. By 1997, 40 out of 51 States had passed this law.<br>4) Minimum Legal Drinking Age 21 (MLDA-21) laws make it illegal for an individual under the age of 21 to drink. This law went into effect in every State during the 1980s.<br>5) Zero Tolerance laws make it illegal for an individual under the age of 21 to operate a vehicle with a BAC level of .02 or more. By 1997, 47 out of 51 States had passed this law.	- In general, this study supports earlier findings that alcohol laws such as BAC, ALR, Zero Tolerance, and MLDA-21 significantly reduced (from 1982 to 1997) the proportion of drivers involved in fatal crashes who had BAC of .08 or higher as well as those who had BAC of .01 or higher (44% of change).<br>- Each individual law was associated with a statistically significant reduction in alcohol-related driving for both groups of drivers (BAC of .08 or above and BAC of .01 or above): MLDA-21 and Zero Tolerance laws reduced alcohol involvement by individuals less than 21 years of age, while .10 BAC, .08 BAC, and ALR laws reduced alcohol involvement at all ages.	- The leveling off after 1997 does not in any way suggest that the laws are becoming less effective. On the contrary, they continue to successfully hold (1) the proportion of drivers with a BAC of .08 or above and (2) the proportion of drivers with BAC of .01 or above, close to the historically low 1997 rates. But there has been little additional improvement because the laws were already in effect in most of the States by 1997, and the demographic changes that reduced impaired driving had also leveled off.<br>- Although enforcement and activism (MADD, SADD) could not be directly included as variables in the model, it could be argued that their effect is implicitly present. Parts of the fatality reductions that the model attributes to the various laws is a consequence of the enforcement and publicity activities that have made the laws effective.<br>- Other potential independent variables mentioned (but not included): sobriety checkpoints, the economy, increased taxes and other important safety legislation (e.g., seat belt laws).	INCLUDE.<br><br>Imputed Fatality Analysis Reporting System (FARS) cases are used.

[123]	First author, year, country	General aim	Study design	The policy measure(s) or intervention(s) at hand	Measured policy effects on MLDA	Reflection on policy and other occurrences by authors	Comments
	Roy, 1979, USA.	The objective of this DUIL (Driving Under the Influence of Liquor) study was to determine the age and sex distribution of drunk driver defendants in the state of Massachusetts, and to assess the incidence of simultaneous offenses by age. This study was undertaken to assess any shifts in age of defendants since the enactment	The Office of the Commissioner of Probation (OCP) analysed data based on court appearance records received from 70 probation departments state-wide from October 1979. This data was compared to records received from February to March, 1979. The OCP is unique in that all criminal and delinquency records statewide are centrally filed and stored in the OCP Central File.<br>Only those records reflecting new charges for drunk driving were included in the samples.	Massachusetts legislation which raised the legal drinking age from 18 to 20 years in April 1979.	- No significant shifts in volume of drunk driver arrests have occurred as a result of the new legislation.<br>- When the October sample was compared to a similar sample in February, this DUIL study found a 26 percent increase in the number of teenagers (15-19 years of age) who were charged with driving under the influence of liquor (n=250 in February, n=316 in October). While teenagers comprised about 14 percent of the February DUIL defendants, they accounted for over 17 percent in October.<br>- Teenagers were found to have a higher than predicted frequency of multiple offenses, including: operating to endanger, use of a motor vehicle without authority, leaving the scene of an accident with property damage and personal injury, crimes against property and public order offenses.	- While one would have expected a sharp reduction in teenage drunk drivers after the change in legislation, this follow-up research found an increased number of arrests among people under 20 years of age. This may be due to: intensive police enforcement. However, it is also likely that some teenagers are probably not honoring the new law. They may be drinking more in cars inasmuch as they cannot legally drink in a tavern or bar.	**INCLUDE.**<br><br>- The (OCP) Central File.

[124]	**First author, year, country**	**General aim**	**Study design**	**The policy measure(s) or intervention(s) at hand**	**Measured policy effects on MLDA**	**Reflection on policy and other occurrences by authors**	**Comments**
	Loeb, 1987, USA.	This study evaluates the efficacy in reducing fatality rates by investigating policy-related variables.	- A cross-section model is used, with data from 1979 relating to all fifty states and the District of Columbia.<br>- Ordinary least squares (OLS) regression were conducted.	- Inspection systems of motor vehicles.<br>- Legal minimum drinking age.<br>- Alcohol consumption.<br>- Average speed.	- Raising the MLDA does not significantly reduce fatality rates, the coefficient associated with this variable while negative, was never significantly different from zero in any specification suggested.<br>- The beer consumption variable was consistent in having a positive and significant effect on fatality rates.<br>- The vehicular speed variable suggests that raising the average speed significantly increase fatality rates.<br>- Inspection of motor vehicles are found to cause a significant reduction in fatality rates.	- The insignificance found for the MLDA variable may be due to the postponement of accidents by youths in the 18-20 age category till they reach the age of 21 or older (i.e., postponing traffic deaths in that age peer group to a future period).<br>- Reducing consumption of malt beverages would result in a significant decline in the fatality rate (e.g., tax reforms).<br>- The empirical results of this investigation provide a powerful argument against increases in average speeds and is in support of imposing or maintaining the inspection of motor vehicles to secure a significant reduction in motor vehicle deaths.	**INCLUDE.**<br><br>- Main data on fatality rates, fuel consumption and highway mileage are from U.S. highway statistics (1979).<br>- Other data are from the American Automobile Association (1980), the US Statistical Abstract (1980) and the World Almanac (1979).

[125]	**First author, year, country**	**General aim**	**Study design**	**The policy measure(s) or intervention(s) at hand**	**Measured policy effects on MLDA**	**Reflection on policy and other occurrences by authors**	**Comments**
	Klepp, 1996, USA.	- The aim of the present study was to assess the effect of the increased minimum drinking age on self-reported drinking and driving behaviors in two consecutive year cohorts (born before 1967 and born after 1967).	- The cohorts were comprised of students originally from the same suburban seventh-grade school districts in Minnesota, their drinking and driving were assessed across two consecutive years surrounding the change in drinking age law, allowing comparison of the cohorts at the same age (drinking and driving survey-data were collected in 1987 and 1988).<br>- The survey focused on (limited to the alcohol-subject) drinking and driving behavior, asking participants:<br>1) whether they had a license to drive a motor vehicle and how many alcoholic drinks they had 'been able to handle and still manage to drive during the last three months;<br>2) how often during the past three months they had two or more drinks within one or two hours before driving.<br>- Participants were divided into two cohorts (before 1967, the 'older cohort' and after 1967, the 'younger cohort').<br>- Data were analyzed using procedure GLM in SAS.	The raise of the minimum drinking age law in Minnesota from 19 to 21 in 1986.	- The younger cohort in 1988 (having been able to handle at least one drink and still manage to drive within the last three months) reported significantly less drinking and driving than the older cohort in 1987 (51% vs. 61%; $p < .000$).<br>- Fewer of the younger cohort in 1988 (having been able to handle at least one drink and still manage to drive within the last three months) reported driving after two drinks than in the older cohort in 1987 (29% vs. 35%; $p < .000$).<br>- Asking both cohorts whether they had five or more drinks within two hours prior to driving, there were significant differences in the prevalence (16% vs. 18%; $p < .04$).<br>- When asked "what was the largest number of alcohol drinks that you, personally, have been able to handle and still manage to drive during the past three months", significantly fewer drinks were reported by the younger cohorts in 1988 compared to the older cohort in 1987 (2.1 drinks vs. 2.6 drinks).	- The increased minimum drinking age appears to have been effective in reducing the overall drinking and driving prevalence primarily by deterring drivers who had been drinking one or a few drinks.<br>- Despite the small reduction in drinking and driving, the results from this study provide some support for the notion that reduced alcohol availability actually reduced the overall rates of drinking and driving among underage consumers.	INCLUDE.<br><br>- Surveys for the junior high school smoking prevention studies.

[126]	First author, year, country	General aim	Study design	The policy measure(s) or intervention(s) at hand	Measured policy effects on MLDA	Reflection on policy and other occurrences by authors	Comments
	Fell, 2009, USA.	To examine which laws that target youthful drivers and which laws that affect drivers of all ages are effective in reducing alcohol-related crash fatalities among young people. The study had two primary objectives: (1) to determine if the enactment of six MLDA-21 laws was associated with reductions in the rate of underage drinking to nondrinking drivers involved in fatal crashes after the effective date, and (2) to determine if the implementation of other key drinking-and-driving laws and socioeconomic conditions in the states had an effect on fatal crashes involving underage drinking drivers.	- A pre- and post-law design was used including data of Crash Incidence Ratio (CIR, i.e., the ratio of crash involved drinking drivers to crash-involved nondrinking drivers) of drivers ages 20 and younger and drivers aged 26 and older, covering a 23-year period from 1982 through 2004. The data were analysed using Structural Equation Modelling (SEM) techniques.<br>- Mediation analysis was performed estimating the indirect effect of per capita beer consumption in the associations between the investigated laws and CIR.<br>- To adjust for most of the extraneous factors within both groups of drivers, non-alcohol-related crashes were used as a control group.<br>- Alcohol involvement was documented through BAC test results collected by police or coroners.<br>- Covariates used are: The frequency of sobriety checkpoints, Per capita beer consumption rates, a latent "strong economy" variable represented by the employment rate and VMT (vehicle miles traveled) per capita.<br>- Although it would be preferable to use beer consumption measures that are specific to each age group cohort, those measures are not available, so the overall rate for a state must therefore serve as a proxy measure for beer consumption within each component age group.	- We selected six underage drinking laws for analysis: (1) a law making it illegal for youth aged 20 or younger to publicly possess alcohol, (2) a law making it illegal for youth aged 20 or younger to purchase alcohol, (3) a law mandating that beer kegs be registered to the purchaser, (4) ZT (zero-tolerance) for alcohol in drivers aged 20 or younger, (5) GDL (graduated driver licensing) with night restrictions for novice drivers (intended to reduce the risk of underage drinking and driving), and (6) a use and lose law resulting in a driver's license suspension for an alcohol violation for youth aged 20 or younger.<br>- The possession and purchase (1 and 2) laws are core MLDA-21 laws; their effective dates were the same, so they were treated as one law.	- Significant decreases in the underage fatal Crash Incidence Ratio (CIR) were associated with the implementation of possession and purchase laws (−16%, p < .001), the ZT law (−5%, p = .015), and the use and lose law (−5%, p = .026), and three of the laws targeting all drivers (.08 blood alcohol concentration illegal per se law, secondary or upgrade to a primary seat belt law, and an administrative license revocation law).<br>- Beer consumption was associated with a significant increase in the underage fatal CIR.<br>- The direct effects of laws targeting drivers of all ages on adult drinking drivers aged 26 and older were similar but of a smaller magnitude compared to the findings for those aged 20 and younger.	- These results provide substantial support for the effectiveness of underage 21 drinking laws, with four of the six laws we examined having significant associations with reductions in underage drinking-and-driving fatal crashes.<br>- Beer consumption was also positively related to CIR of < 20 years; every additional gallon of ethanol consumed (per capita) was associated with a 44% increase (*p* < .001) in the underage alcohol- related fatal CIR.<br>- Nevertheless, the decreases in fatal CIR <21 years due to the enactment of core MLDA-21 laws could not be explained by increases in per capita beer consumption.<br>- Only GDL with night restrictions and keg registration laws failed to show significant associations with fatal CIR reductions. This does not necessarily mean these laws are not effective (although that is one possible conclusion); it could mean that any effects were not detected in this study using these measures.<br>- There are at least 16 underage drinking laws that have been adopted by some of the states that could have an impact on underage impaired driving. We obtained the legislative effective dates from the states for 6 of the 16 laws. Effective dates for the remaining 10 underage laws are not readily available and should be accessed to help states decide what their legislative agenda should be when it comes to reducing underage drinking and its consequences.	**INCLUDE.**<br><br>- National Institute on Alcohol Abuse and Alcoholism (NIAAA) Alcohol Policy Information System (APIS) data set (1998–2005).<br>- Annual state-level data from NHTSA's Fatality Analysis Reporting System (FARS) from 1982 to 2004 were used.

Unintended developments and implications

[35]	First author, year, country	General aim	Study design	The policy measure(s) or intervention(s) at hand	Measured policy effects on MLDA	Reflection on policy and other occurrences by authors	Comments
	Robertson, 1989, USA.	This study examines the amount of alcohol in the blood of fatally injured drivers per licensed driver in states where more than 80 percent of such drivers were tested for alcohol and where licensure data were available for the period 1982 – 1986.	Spill over effects, the legal-age effect and the experience effect were estimated calculating least-squares regression coefficients.	Legal Drinking Age (LDA) of 18 and 21 (in multiple states).	- A higher LDA is associated with fewer alcohol related crashes among those younger that the LDA (not sig.).<br>- The involvement of alcohol in fatal crashes increases with age as the LDA is approached (not sig.).<br>- No effect of drinking experience was evident.<br>- No evidence is found of a short-term spurt effect when teenagers reach the LDA, or of longer-term drinking experience reducing alcohol involvement in fatal crashes.	- Although it is obvious that all teenagers who have not reached the MLDA do not abstain from drinking, the data in this study suggest that a higher MLDA reduces fatal crashes among those below the LDA. The further the driver's age is below the LDA, the lower the alcohol-related crash rates of those drivers.<br>- The correlation among age, alcohol involvement, and night or single vehicle crashes (as is conducted in previous studies) has apparently contributed to an underestimation of the 'spillover' effect of lower LDAs on younger-than-legal-age drivers in previous studies using night and single-vehicle crashes as proxies for alcohol involvement.	**INCLUDE.**<br><br>- Data is used from the National Highway Traffic Safety Administration (FARS data).

[38]	First author, year, country	General aim	Study design	The policy measure(s) or intervention(s) at hand	Measured policy effects on MLDA	Reflection on policy and other occurrences by authors	Comments
	Wagenaar, 1983, USA.	The purpose of this study was to assess the effect of raising the drinking age in Maine on fatal, injury and property damage crash involvement among young drivers.	- Monthly frequencies of motor vehicle crashes among drivers aged 18-45 in the states of Maine and Pennsylvania (comparison state without legislative changes) from 1972 through 1979 were examined.<br>- Information was gathered from Maine accident report forms provided by police officers investigating crashes (whether the driver had been drinking was used as one indicator of alcohol-related crash involvement).<br>- Additional data on single-vehicle nighttime male crashes were used as a comparison for police reported data.<br>- A multiple time series design was used, controlling for the effects of long-term trends, seasonal cycles and other factors with Box-Jenkins time series models.	In 1977, the legal drinking age in Maine was raised from 18 to 20.	- Raising the legal drinking age in Maine resulted in significant reductions in youthful alcohol-related property damage crashes (measured both by police reports and comparison analyses, an estimated 16.8% and 21.5% fewer 18- to 19-year-old drivers were involved in alcohol-related property damage crashes after the drinking age was raised), but had no demonstrable effect on the incidence of alcohol-related injury and fatal crashes among young drivers.	A plausible assumption for not finding effect on the incidence of injury and fatal crashes could be that young drinking drivers involved in injury and fatal crashes are likely to be heavier drinkers than drinking drivers involved in property damage chases. One could then speculate that the findings of the present study indicate that the drinking-driving behavior of heavy drinkers is less affected by the raised drinking age than drinking driving behavior of moderate drinkers.	**INCLUDE.**<br><br>-

[40]	**First author, year, country**	**General aim**	**Study design**	**The policy measure(s) or intervention(s) at hand**	**Measured policy effects on MLDA**	**Reflection on policy and other occurrences by authors**	**Comments**
	Rock, 1991, USA.	The impact on traffic accidents of changes in the drinking age in Illinois was studied.	- A data set suitable to analyze the impact of these changes is compiled monthly by the IDOT. For the entire state from 1970 to mid- 1989, traffic accident totals (also broken down by fatal, injury, or property damage only), age of drivers involved, and road conditions were selected.<br>- Using auto-regressive integrated moving average (ARIMA) techniques and total accidents by month over a 20-year period, the experience in Illinois is reexamined.	Illinois lowered their drinking age for beer and wine from 1973 to 1980 to 19 and raised it in 1980 from 19 to a uniform MLDA of 21.	- The lower drinking age in Illinois was responsible for an increase of more than 5,000 accidents per month, a 14 percent increase.<br>- When the age was raised back to 21 in 1980, the figures reversed a similar amount (sig.).	- The Illinois data set could be used to examine other areas of interest and controversy. For example, it is alleged that those immediately under the drinking age are more likely to obtain alcohol illegally than those who are farther below. With an increase of the drinking age to 21, one could examine the percentage reductions in accidents in the younger age groups.<br>- A variety of other public policies could be examined. Laws enacted in Illinois have allowed right turn on red (1973), lowered the maximum speed limit to 55 mph (1974), raised the maximum speed limit on most Interstate highway segments to 65 mph (1987), and mandated the use of seat belts (1985).<br>- Changes in the drinking age in bordering states are relevant; for a number of years the lower drinking age in Wisconsin attracted many younger drivers from Illinois.<br>- The short- versus long-term effects can differ: short-term impacts can dissipate if below-age drinkers obtain alternative sources of alcohol, or they can be reinforced if new cohorts of younger drivers do not develop patterns of driving after drinking.<br>- An alternative explanation, raising the drinking age does not reduce fatalities, more lives are lost among older drivers than are saved among younger drivers. An extension to this was that inexperience in drinking, independent of age, was the major hazard.	**INCLUDE.**<br><br>- The Illinois Department of Transportation (IDOT) data was used.

[53]	First author, year, country	General aim	Study design	The policy measure(s) or intervention(s) at hand	Measured policy effects on MLDA	Reflection on policy and other occurrences by authors	Comments
	Jager, 2015, USA.	- Drawing from a developmental-contextual perspective, and using multi-cohort panel data, we examine how the trajectories of age 18–26 binge drinking (i.e., consuming five or more drinks in a row), have changed across the last three decades.<br>- We focus on two components of the chronosystem that may help explain historical variation in young adult binge drinking trajectories: (a) historical variation in the frequency and timing of social role acquisition and (b) historical variation in minimum legal drinking age (MLDA).	- As part of the national Monitoring the Future Study, over 64,000 youths from 28 consecutive cohorts of high-school seniors between 1976 and 2004 were surveyed at biennial intervals between ages 18 and 26.<br>- We focused on the first 5 waves of the MTF panel data, using Full Information Maximum Likelihood estimation analyses.<br>- The output measure of this study is 'binge-drinking', operationalized as 5 or more drinks in a row in the past two weeks.	- Historical increases in minimum legal drinking age.<br>- The 'Big 5' young adult social roles (marriage, parenthood, education, residential status, and employment) were also outcome of interest but not a measure or intervention.	- For both genders, the positive quadratic effect of cohort year on age 18 binge drinking was no longer significant once MLDA laws were controlled, thus linking MLDA laws that were implemented in the mid-1980s with the sharp decline in age 18 binge drinking across the mid-1980s and early 1990s (sig.).<br>- We found that historical increases in minimum legal drinking age account for a portion of the historical decline in age 18 levels of binge drinking, while historical variation in social role acquisition (e.g., marriage, parenthood, and employment) accounts for a portion of the historical acceleration in age 18–22 growth.	Future research should focus on other proximal contextual factors that are known to be associated with binge drinking and have potentially varied historically (in both linear and non-linear fashions). Although by no means an exhaustive list, the following are known to be associated with binge drinking: the perceived risk and availability of alcohol, parental attitudes as well as peer norms regarding alcohol use, and alcohol abuse prevention efforts, including school- based programs, media campaigns.	**INCLUDE.**<br><br>- Data from the 1976–2004 Monitoring the Future (MTF) surveys were used.

[56]	First author, year, country	General aim	Study design	The policy measure(s) or intervention(s) at hand	Measured policy effects on MLDA	Reflection on policy and other occurrences by authors	Comments
	Laixuthai, 1993, USA.	This paper examines the frequency of youth drinking and heavy drinking in 1982 and 1989 to 1) investigate the effects of MLDA and alcoholic beverage prices on youth drinking, and 2) investigate the effects of a uniform age of 21 on the price sensitivity of youth alcohol use.	- Dichotomous probit methods are used for analysis.<br>- Categorical information on the number of drinking occasions in the last 30 days, the last year, and in the respondent's lifetime, as well as the number of times in the last two weeks that he or she had five or more drinks in one occasion is obtained. This information was rearranged in comparable variables for analysis.	- The MLDA and federal excise taxes on beer (in 1982 and 1989).	- Increased alcoholic beverage prices and/or minimum legal drinking ages reduce the frequency of alcohol consumption and heavy drinking by youths (sig.).<br>- Reductions in drinking are not limited to infrequent drinkers only, even larger reductions occur in the numbers of youths who drink frequently or fairly frequently.<br>- The price sensitivity of youth alcohol use fell after the change to a uniform legal drinking age of 21.<br>- Underage youth who live within 25 miles of a state with a lower MLDA apparently cross the border to obtain alcoholic beverages.	The full price of consuming alcohol for a youth is the sum of both the money price and the indirect costs (the legal obstacles to consumption, such as a higher MLDA). Other indirect costs include the money and time to obtain a fake ID, to obtain the alcohol itself and others. Increased MLDA and taxes both increase the full price of drinking for underage youth. In 1989, high school seniors face a greater indirect cost of obtaining alcohol than they did in 1982 due to the uniform MLDA of 21. Therefore, the same increase in tax has a smaller impact on full price in 1989 (and consequently, consumption), than in 1982.	**INCLUDE.**<br><br>- Data from the 1982 and 1989 Monitoring the Future (MTF) surveys.<br>- Data from the American Chamber of Commerce Researchers Association is used for the tax variable.

[57]	First author, year, country	General aim	Study design	The policy measure(s) or intervention(s) at hand	Measured policy effects on MLDA	Reflection on policy and other occurrences by authors	Comments
	Miron, 2009, USA.	We challenge the view that MLDAs reduce traffic fatalities based on three findings. First, the overall impact estimated in earlier research is driven by states that increased their MLDA prior to any inducement from the federal government. Second, even in early-adopting states, the impact of the MLDA did not persist much past the year of adoption. Third, the MLDA has at most a minor impact on teen drinking.	- We examine the relation between MLDAs and traffic fatalities using aggregate road facilities data of the complete population and of 15-24-year-olds and state-level panel data, reconstructing the analysis of Dee (1999) (using FARS to construct a panel data set for the 48 contiguous states over the period 1977–1992) and extending it to include Alaska, Hawaii, and Washington DC, and the years 1976 and 1993–2005. We focus on 18- to 20-yr-old fatalities.<br>- Using MTF data, we employ the two specific measures common in the literature, ‘‘drinker’’ (having any drink of alcohol in the last month) and ‘‘heavy episodic drinker’’ (having five or more drinks in a row at some point in the last 2 weeks). We also examine the number of motor vehicle accidents that respondents report as occurring after consuming alcohol. We estimate regressions similar to the traffic fatality calculations with these dependent variables. The measure of the MLDA is identical to that used in previous literature, a dummy for having a drinking age of 18 years.	- Changes in de MLDA over the period 1976-2005.<br>- Mandatory seatbelt law.<br>- The BAC limit for legal driving.<br>- Beer taxes.<br><br>We omit several potentially relevant policies, in part because of data availability, in part to conform with Dee (1999), and in part because previous studies have found limited evidence of any impact on TFRs. These variables include dram shop liability laws, mandatory sentences for driving under the influence, sobriety check points, anti-plea-bargaining statutes, changes in tort liability laws that place greater responsibility with intoxicated drivers, happy hour regulations, enforcement (however, enforcement is too low to have any impact on the results examined) and alcohol education programs.	- The MLDA fails to have the fatality-reducing effects that previous articles have reported; trends between 1910 and 2000 in aggregate data of road facility rates show no difference in trends between 15-24-year-olds and that of the entire population.<br>- The declines in the total and 15–24 TFR that began around 1969 long precede the adoptions of an MLDA of 21 in the mid-1980s. The key fact about TFRs, therefore, is that they have been trending downward for decades and have been poorly correlated with the MLDAs.<br>- State-level panel data for the past 30 years show that any nationwide impact of the MLDA is driven by states that increased their MLDA prior to any inducement from the federal government (FUDAA). Even in early-adopting states, the impact of the MLDA did not persist much past the year of adoption.<br>- The MLDA appears to have only a minor impact on teen drinking (sig.), these reductions derive mainly from states that adopted the MLDA21 before enactment of the FUDAA.<br>- Nevertheless, when the number of accidents post-alcohol consumption was analyzed, the panel estimates reveal that movement away from an MLDA of 18 is associated with a statistically insignificant change in reporting of alcohol-related traffic accidents.	- These results suggest that, at most, the MLDA21 reduced TFR18–20 in states that adopted the policy on their own. This raises the question of endogeneity. The MLDA21 in these states may have been enacted in response to grassroots concern against drunk driving or implemented alongside other efforts to reduce traffic fatalities. Relatedly, states that adopted on their own may have been states that devoted significant resources to enforcement.<br>- The limited effects found in non-early adopting additionally challenges the desirability of coercive federalism.<br>- Landmark improvements in the accident avoidance and crash protection features of passenger cars (in place around 1970), and control for advances in medical technology might explain the drastic reductions in traffic fatalities over the past half century and should be made to additionally measure and control for as they are likely to be major determinants of the declining traffic fatality trends.	**INCLUDE.**<br><br>- Data from the Fatal Accident Reporting System (FARS) were used.<br>- Data from Monitoring the Future (MTF) surveys were used.

[60]	First author, year, country	General aim	Study design	The policy measure(s) or intervention(s) at hand	Measured policy effects on MLDA	Reflection on policy and other occurrences by authors	Comments
	Plunk, 2013, USA.	This present study uses changes in MLDA laws during the 1970s and 1980s as a natural experiment to investigate the potential impact of permissive MLDA exposure on average alcohol consumption, frequency of drinking, and patterns of binging and more moderate, nonheavy drinking.	- Policy exposure data were paired with alcohol use data, including past-year drinkers born between 1948 and 1972 (n = 24,088). Average daily intake, overall drinking frequency, and frequency of both binge episodes (5+ drinks) and days without a binge episode (nonheavy drinking) for the previous year at the time of interview were tracked for each respondent.<br>- We extend the 2-by-2 analysis to multiple groups and times by including state and birth-year fixed- effects categorical variables to all models.<br>- Multinomial logistic regression was used for the main analyses, wherein we modelled the relative odds of the 3 drinking frequency categories for binge and nonheavy drinking occasions related to permissive MLDA exposure. Logistic, linear, and negative binomial regressions were used for ancillary analyses, to investigate the potential effect of MLDA exposure on drinking status (e.g., lifetime abstainer vs. past-year drinker), average ounces of alcohol per day, and total drinking days, respectively.	A period of MLDA change that occurred between the early 1970s and mid-1980s.	- Exposure to permissive MLDAs was associated with higher odds to report frequent binging (15% higher odds to binge more than once per month, compared with the odds of having had no such occasion) and to report any moderate drinking (sig.) (in other words, that frequent binge drinking becomes more common, while any nonheavy drinking behavior among drinkers becomes less frequent).<br>- These associations were largely driven by men and those who did not attend college (sig.).<br>- Overall drinking frequency and average alcohol consumption were not affected by MLDA exposure (not sig.).	- Exposure to permissive <21 MLDA laws seems to be associated with a certain pattern of drinking behavior that persists into later adulthood, namely, that frequent binge drinking becomes more common, while any nonheavy drinking behavior among drinkers becomes less frequent.<br>- Going to college was associated with a decreased potential MLDA exposure effect. Binge drinking has decreased in the general population, but has remained common on college campuses with the campus environment—characterized by easy access to alcohol coupled with a culture that promotes drinking—likely insulating against policies aimed at restricting underage access to alcohol. We propose that our findings offer support for this campus insulation effect because of ease of alcohol availability, whereby policy exposure for those individuals who attended college would have been substantively different compared with their noncollege peers of the same age.<br>- We should not overly focus on college students when assessing how MLDA affects youth and young adult drinking behavior. While college campuses are arguably conducive to heavy drinking irrespective of policies intended to curb underage alcohol use, in our sample, individuals outside the college environment seem to have been greatly affected by changes in MLDA.<br>- Laws intended to penalize or curb youth possession or consumption in other ways then the ability to legally purchase alcohol were not included in our analyses. There were also no allowances made for smaller jurisdictions within a state that might have had different policies that could further limit access (e.g., dry counties). There could also be other factors influencing lifetime drinking patterns (e.g., cross-state migration).	**INCLUDE.**<br><br>- The 1991–1992 National Longitudinal Alcohol Epidemiological Survey (NLAES).<br>- The 2001–2002 National Epidemiological Survey on Alcohol and Related Conditions (NESARC).

[62]	First author, year, country	General aim	Study design	The policy measure(s) or intervention(s) at hand	Measured policy effects on MLDA	Reflection on policy and other occurrences by authors	Comments
	Smith, 1984, USA.	The effects on the raised MLDA in Massachusetts is examined on drinking, drinking and driving, and nonfatal and fatal crash involvement of 16-17-year-olds (teenagers immediately younger than those targeted by the law).	- Data from Massachusetts are compared with those from New York, where the MLDA remained at 18.<br>- A total of 3 years of survey data from the two states and 6 years of data from FARS were used for pre- and post-law comparisons.<br>- An anonymous random digit-dialing telephone survey was conducted in Massachusetts prior to enactment of the law in 1979, asking teenagers about personal characteristics, drinking practices, procurement of alcohol, use of psychoactive drugs, driving after drinking and nonfatal accident involvement. A similar survey was conducted in the New York area (respondents were asked for their drinking behavior and behavior on how they obtained their alcohol comparable to the New York sample).<br>- Twice at yearly intervals following enactment of the law, surveys of similar size were repeated in each state.<br>- Log-linear analysis was used on the survey data.<br>- In addition, FARS data was used for both states from 1976 to 1982 (single-vehicle nighttime accidents were examined separately).<br>- Data were fitted to a log-linear model, an analysis of variance and an analysis of covariance.	The 1979 Massachusetts law raising the MLDA from 18 to 20.	- The findings suggest that raising the MLDA had minimal effects on the drinking behavior of Massachusetts teenagers.<br>- There was a significant decline in Massachusetts in the number of teenagers reporting drinking at a bar/club/restaurant or in an automobile, an increase of teenagers drinking at parties and a decrease in the number of teenagers purchasing alcohol at liquor stores after the raise of the MLDA and compared to New York.<br>- Massachusetts 16-17-year-olds were more likely in each of the 2 post-law years to have had others purchasing alcohol for them (compared to New York).<br>- After the enactment of the law, driving after drinking declined significantly in Massachusetts relative to New York.<br>- Our analysis did not reveal a significant difference in single-vehicle nighttime fatal accidents (and total fatal accidents) among 16-17-year-olds in Massachusetts and New York after the enactment of the law.	- The present findings suggest that the effect of the MLDA changed where these teenagers drink and how they obtain alcohol.<br>- It is interesting that both before and after enactment of the law, 16-17-year-olds in New York drove less frequently after drinking compared to Massachusetts teenagers. Perhaps, this is the result of having a stable (but lower) drinking age in New York over several decades.<br>- Changes in drinking age may offer some reduction in teenage traffic crash involvement, but teenage drinking and teenage driving after drinking remain serious problems, even in states that raise their MLDA.<br>- The present study indicates lawbreaking among young people, perhaps fostering cynicism toward the legislative process and disregard for law enforcement. As long as teenagers are never asked for ID or have others purchase alcohol for them, significant declines in harm are unlikely.	**INCLUDE.**<br><br>- Data from the Fatal Accident Reporting System (FARS).<br><br>- Hingson et al. (1983) examined the first 2 post-law years in Massachusetts [44].

[63]	First author, year, country	General aim	Study design	The policy measure(s) or intervention(s) at hand	Measured policy effects on MLDA	Reflection on policy and other occurrences by authors	Comments
	Subbaraman, 2013, USA.	The primary aim of this study was to evaluate the effects of both raising and lowering the MLDA on per capita ethanol (EtOH) consumption in longer and more accurate time series panel than any previous study.	Generalized least squares model specifications controlling for income, unemployment rates, and population characteristics were implemented using MLDA and aggregate EtOH consumption data from U.S. states from 1950 to 2002.	The effects of both lowering and raising the MLDA.	- Estimates from the full 1950 to 2002 period which include both the lowering and raising of the MLDA indicate that raising the MLDA from 18 to 21 decreased total consumption by 1.51%, beer consumption by 2.31%, and spirits consumption by 1.86% across drinkers of all ages, *implying* substantial changes among underage drinkers (the effects observed relate the MLDA to total alcohol sales across drinkers of all ages, not just among 18- to 20-year-olds).<br>- Results for the later 1976 to 2002 period do corroborate that the MLDA effects observed for the entire 1950 to 2002 period may be largely attributed to raising the MLDA. Increasing the MLDA by 3 years (i.e., from 18 to 21, as was generally done) during this period significantly predicted a 2.25% decrease in per capita total consumption, a 1.8% decrease in beer consumption, and a 3.36% decrease in spirits consumption. These estimates are quite substantial considering that only those under the age of 21 were impacted by the law.	- These results add to the mounting evidence that increasing the MLDA decreases total EtOH consumption as well as alcohol-related harms.<br>- Others have proposed that alcohol policies may interact advantageously. Ponicki and colleagues (2007) reported that raising the MLDA and raising beer taxes independently appeared to reduce fatal motor vehicle accidents in 48 U.S. states from 1975 to 2001 and that increasing the MLDA appeared to reduce proportionately more accidents when taxes were high compared with when taxes are low, suggesting that alcohol policies may work synergistically.	**INCLUDE.**<br><br>- Government and industry beverage-specific sales volume data and used year- and state-specific estimates of mean EtOH content for each beverage type.<br>- MLDA data came from the Prevention Research Center's Statewide Availability Data System.

[64]	**First author, year, country**	**General aim**	**Study design**	**The policy measure(s) or intervention(s) at hand**	**Measured policy effects on MLDA**	**Reflection on policy and other occurrences by authors**	**Comments**
	Buijs (chapter 3, in Van Havere), 2017, Belgium.	The analyses carried out in this chapter were set out to investigate four research questions.<br>- Firstly, it was investigated whether or not the proportion of life-time abstainers, weekly drinkers and binge drinkers evolved differently between adolescents aged under 16 and those older, at the regional and national level.<br>- Secondly, the evolution of life-time and weekly consumption of distilled spirits among adolescents under the age of 18 and those older was investigated at the regional and national level.<br>- A third research question widened the perspective by investigating trends in life-time and weekly alcohol consumption as well as life-time drunkenness among adolescents aged 11-15 years old in 30 countries. This was done in order to analyze the impact of the minimum legal drinking age, as well as a wider array of policies and alcohol affordability, on the outcome measures.<br>- The fourth research question was investigated at the national and international level and concerned the role of socioeconomic status on the different outcome measures.	-	- The impact of the minimum legal drinking age in Belgium. - In December 2009, the Belgian government amended its existing alcohol legislation, to prohibit access to distilled alcoholic beverages to adolescents under the age of 18 and access to all (both distilled and fermented) alcoholic beverages to adolescents under the age of 16. This restriction applied to both the possession by adolescents, as well as alcohol sales by retailers, bars and restaurants.	- Concerning research questions 1 and 2, the analyses at the Flemish and Belgian level showed significant statistical interactions between age and time. This indicates that over the research period, alcohol consumption patterns in the different age groups (under 16 versus older for fermented drinks, under 18 versus older for distilled drinks) have evolved differently, in the sense that the odds of consuming alcohol decreased at a higher pace for those under the respective age limit. This could indicate towards an effect of the law on minimum legal drinking age.<br>- At the international level, similar relations were found between alcohol consumption, age and gender in the group of 11-15 years old. Moreover, the significant time trend, which was observed in Belgium, was also found in the international sample, indicating that overall, alcohol consumption has decreased in the 2002-2014 period. Therefore, no specific evidence was found that supports an impact of the Belgian drinking age legislation on adolescent alcohol consumption.<br>- Furthermore, supplemented analysis on the Belgian MLDA investigating "what works" to reduce alcohol consumption in adolescents younger than 16 years old found mixed results. The minimum legal drinking age was found to be non-significant in relation to life-time and weekly alcohol consumption and positively related to life-time drunkenness indicating that countries with higher minimum legal drinking ages typically have a higher proportion of life-time drunkenness.<br>- When evaluating a wider array of policies that restrict alcohol availability, a significant relation was found between stricter policies and lower odds of weekly alcohol consumption.<br>- The effect of marketing restrictions and affordability changes were also measured. Current marketing restrictions were not found to have a significant effect on alcohol outcome measures.<br>- Affordability changes on the other hand were highly significant with increased affordability being linked to higher odds of alcohol consumption: when alcohol is cheaper, adolescents tend to drink more.	- Although indications towards an effect of the MLDA law were found, it could also be due to other events (e.g. 2008 financial crisis) or increased alcohol prices.<br>- It remains uncertain what the Belgian consumption trend would have looked like in the absence of the 2009 minimum legal drinking age legislation.<br>- It is important to note that in the mixed results in the "what works" analyses, reversed causality could play a role, i.e. that countries which have a higher proportion of life-time drunkenness institute higher minimum legal drinking ages.<br>- An important caveat in the wider array of analyses on policy, is the failure to incorporate enforcement of the above policies into the regression analysis. Such information was not systematically available for the complete international sample.<br>- The results also show that the combination of policy measures (the Total Policy Index represents the mean of the availability index, the affordability index and the marketing restrictions index) can be effective in the reduction of both life time and weekly alcohol consumption.	**INCLUDE.**<br><br>- Methodological report describing study design not found.

[66]	First author, year, country	General aim	Study design	The policy measure(s) or intervention(s) at hand	Measured policy effects on MLDA	Reflection on policy and other occurrences by authors	Comments
	Coate, 1987, USA.	This article presents the first set of estimates of the responsiveness of youth alcohol use and motor vehicle death rates to variations in the price of alcohol. In addition, it examines the sensitivity of these two outcome measures to changes in the legal drinking age.	- Two survey datasets are used (NHANES I and II) between May 1971 and June 1974, and between February 1976 and February 1980.<br>- In studying youth demand for alcoholic beverages, we focus on alcohol use by youths aged 16 through 21.<br>- Infrequent and frequent beer consumption was measured by asking for the frequency of drinking beer in a week (no more information is provided).<br>- Data (vehicle accidents) based on a time series of state cross sections for the period from 1975 through 1981.<br>- There age groups were focused on: youths aged 15 through 17, 18 through 20 and 21 through 24.<br>- For both aims, estimates were obtained using variants of multiple regression analysis (controlling for multiple variables, e.g., border-hopping, age, family income, vehicle miles traveled per licensed driver).	- The upward trend in state MLDA for the purchase and consumption of alcoholic beverages.<br>- Increased taxation of alcoholic beverages (mainly focusing on beer).	- Use of alcohol by youths declines when either the price of alcoholic beverages or the MLDA increases. Youth alcohol consumption is inversely related at a statistically significant level to beverage price and MLDA, for both infrequent and frequent/heavy drinkers.<br>- Analysis indicate a statistically significant decline in the motor vehicle accident mortality rate with the effects of a real beer tax for youths aged 15 through 17, 18 through 20 and 21 through 24.<br>- Changes in the MLDA also produced statistically significant inverse effects on accident mortality for youths aged 18 through 20 (affecting the mortality of only those young people who might have legally purchased alcoholic beverages at the lower MLDA).<br>- Positive and significant coefficients of the border variable were obtained for the 18-through-20-year-old cohort.	- If reductions in youth alcohol consumption and motor vehicle accident deaths are desired, the preceding figures suggest that both a uniform MLDA of 21 and an increase in the Federal excise tax rate on beer are effective policies to accomplish this goal.<br>- They also suggest that the tax policy may be more potent than the MLDA policy.<br>- An excise tax increase lowers the death rates of youths between the ages of 15 and 17 and between the ages of 21 and 24. These age groups do not receive the same benefits from a rise in the MLDA, and also reduce fatal crashes involving adult drivers. However, a tax hike may greatly stimulate the demand for illegally produced beer.<br>- Young drivers may respond to increasingly severe penalties for offenses only if the possibility of apprehension and conviction is not trivial. If substantial resources must be allocated to increasing the probability of arrest and conviction, a policy of increased excise taxes may be preferable to or complementary with a system of penalties.	**INCLUDE.**<br><br>- the first and second National Health and Nutrition Examination Surveys (NHANES I and NHANES II).<br>- Data on youth motor vehicle accidents mortality (FARS).

[68]	First author, year, country	General aim	Study design	The policy measure(s) or intervention(s) at hand	Measured policy effects on MLDA	Reflection on policy and other occurrences by authors	Comments
	Williams, 1988, USA.	Utilizing survey data for one year before, one year after and 3 years after New York raised its purchase age from 18 to 19 years, this study examines the short-term alcohol purchasing and long-term purchasing and consumption patterns of 16- to 20-year-olds.	A three-stage, stratified proportionate random sampling design was used to select approximately 1,800 16- to 20-year-old New Yorkers living in households.<br>- Anonymous telephone interviews were conducted in 1982 before New York raised its minimum legal purchase age for alcoholic beverages to 19 years. A second sample was interviewed in 1983, approximately one year after the new purchase age law and a third sample utilizing the same design was interviewed in 1985, approximately 3 years after the new purchase age went into effect.<br>- Alcohol purchasing was determined by analyzing survey questions that asked respondents, "In the past 4 weeks did you purchase any beer (liquor) in bars, clubs or restaurants (stores)?" Consumption prevalence levels were established by analyzing survey questions that asked respondents: "How many days in the past 4 weeks did you have a drink?<br>- All comparisons are tested using a proportions test which yields a z-score by comparing the appropriate prevalence measures.	The raise of the MLDA of the state of New York from 18 to 19 in 1982.	- In 1983, the year following the purchase age increase, purchasing prevalence measures for 18- year-olds were significantly lower when compared to 19- and 20-year-old measures for all categories of purchasing.<br>- Eighteen-year-old purchasing measures decreased significantly for all categories from 1982 to 1983.<br>- On the long term, for all purchasing categories, 18-year-old prevalence was significantly lower than the comparable measure for 19- and 20-year-olds in 1985.<br>- Consumption measures of eighteen-year-olds were lower than measures for 19- and 20- year-olds for all measures, and the differences were significant in seven out of 10 comparisons.	- New York's purchase age increase resulted in significant short-term decreases for reported alcohol purchasing by the age group directly affected by the law change and adds support to earlier findings related to alcohol consumption.<br>- The long-term influence of the higher purchase age was maintained since 18-year-olds continued to show significantly lower prevalence rates of alcohol consumption and purchasing in 1985 when compared to 19- and 20- year-olds.<br>- The short-term changes and patterns for alcohol purchasing among 18-year-olds compared to age groups slightly younger and older strongly suggested a purchase-age-specific effect as opposed to a general youth phenomenon.<br>- Age-specific influence was operating on *purchasing* by 16-, 17- and 18-year-olds and was not evident for reported *consumption*. One such potential influence is the implementation of photo licenses. In 1984 all new licenses as well as all renewals were in the form of a special coated card to prevent tampering and included a photograph of the driver's face. This may have further diminished the capacity of illegal purchasers to obtain alcohol.	**INCLUDE.**<br><br>-

[81]	First author, year, country	General aim	Study design	The policy measure(s) or intervention(s) at hand	Measured policy effects on MLDA	Reflection on policy and other occurrences by authors	Comments
	DiNardo, 1992, USA.	This paper analyzes the impact of increases in the minimum drinking age on the prevalence of alcohol and marijuana consumption among high school seniors in the United States.	- The empirical analysis is based on a large sample of students from 43 states over the years 1980-1989, using log-linear model estimates and a structural probit model estimate.<br>- Consumption is measured as the past 30 day alcohol (and marijuana) participation.	This study used the increases in MLDA between 1980-89 in 43 sample states.	- We find that higher minimum drinking ages reduces the prevalence of alcohol consumption.<br>- Increased legal minimum drinking ages had the unintended consequence of increasing the prevalence of marijuana consumption. The unintended consequence is attributable to standard substitution effects.	- An increased drinking age helps create a climate of societal disapproval for all drug use, not only alcohol. In the case of marijuana, this change in societal 'climate' is not sufficient to offset the large substitution induced by the decreased prevalence of alcohol consumption.	**INCLUDE.**<br><br>- Data from the 1980–89 Monitoring the Future (MTF) surveys

[82]	First author, year, country	General aim	Study design	The policy measure(s) or intervention(s) at hand	Measured policy effects on MLDA	Reflection on policy and other occurrences by authors	Comments
	Decker, 1988, USA.	- It is essential to know whether raising the MLDA to 21 can materially reduce the number of deaths among persons aged 15 through 24 years caused by motor vehicle crashes (MVCs).	- Analyzed the Tennessee MVC data (FARS) for 1980 through 1986. Additionally, a count of licensed drivers by county, population and deaths counts by county, an estimate of total miles driven in Tennessee, an estimate of the total and nighttime miles driven and estimates of the prevalence of self-reported DUI were used.<br>- Three age groups were considered; those aged 15 through 18 years, 19 through 20 years and those aged 21 through 24 years.<br>- Focus was on single-vehicle nighttime (SVN) fatal crashes to obtain a sensitive measure for the extent of alcohol involvement in MVCs.<br>- Rates within a given age stratums were compared by using the incidence density ration method, to compare rates among age groups, two-tailed t-tests were performed.	- In 1984, Tennessee raised its MLDA for possession or purchase of alcohol from 19 to 21 years (the age-law).<br>- In 1982, Tennessee adopted legislation that markedly increased the likelihood of imposition and the severity of penalties for conviction for DUI (the penalty-law).	*(only focusing on effects subsequent to the age law)*<br>- There was a significant and persistent decline (38%) in the *SVN fatality rate* among drivers aged 19 through 20 years following implementation of the age-law (no border effects were found).<br>- Among persons aged 15 through 18 years, in 1985, the year following the introduction of the age-law, the *overall* MVC death rate dropped 24% (sig.).<br>- Similarly, in 1985, the year following introduction of the age law, the *overall* MVC death rate dropped 24% (sig.) among persons aged 19 through 20 years.<br>- During 1980 through 1982, the *crude death rate* for persons aged 15 through 18 years declined 13% for 1883 through 1986 (sig.).<br>- *The mean BAC* in 1985 for youth 15 through 18 years was 48% lower than all other years in the study period (sig.). After implementation of the age-law, the mean BAC for 19 through 20-year-olds showed a 35% reduction (sig.) and 20% (sig.) for drivers aged 21 through 24 years.	- The implementation of a law denying alcohol to persons aged 19 through 20 years caused a sudden and dramatic decline in drunk driving among that age group, an effect still present at the end of the study period.<br>- Stiffened penalties or increased enforcement directed against DUI has been associated with declines in DUI fatality rates, however, beneficial effects began before the legal changes, parallel with increasing publicity and social disapproval stimulating those legal changes, and beneficial effects almost invariably disappeared within a few years.<br>- Our data suggest that publicity and other social influences may have played a particularly important role in producing the prolonged reduction in alcohol related MVC mortality seen in the 15-through 18-year-old age group.<br>- Our data indicate that laws raising the MLDA to 21 can be highly effective in reducing alcohol-related MVCs among drivers aged 19 through 20 years, a group apparently quite resistant to the effects of increased DUI penalties end anti-DUI publicity.<br>- We cannot clearly apportion the responsibility for the benefits among influences link DUI laws or a new MLDA, but each appears contributory.	**INCLUDE.**<br><br>- Data on fatal traffic accidents were taken from the Fatality Analysis Reporting System (FARS).

[83]	First author, year, country	General aim	Study design	The policy measure(s) or intervention(s) at hand	Measured policy effects on MLDA	Reflection on policy and other occurrences by authors	Comments
	Figlio, 1995, USA.	Monthly Wisconsin time-series data from 1976 to 1993 were used to estimate the effects of increased minimum drinking ages on alcohol-related crashes involving teenagers.	- This study analyzes 18-year time series data with monthly observations of alcohol-related crashes in the state of Wisconsin, stratified by age, from 1976 to 1993.<br>- The long time-series were used to utilize Box-Jenkins time series techniques to gauge the effects of the introduction of Wisconsin's minimum drinking age laws.<br>- The over-21 age group whose members could drink legally over the entire time period was used as a control group. - A two-way random effects model was used to determine the extent of border hopping as a result of interstate drinking age differences.	Wisconsin, the state in question in this study, raised its drinking age to 19 in 1984 and to 21 in 1986. Prior to 1986, Wisconsin had a lower drinking age than did its neighbors, suggesting that border hopping may have occurred.	- Raising the drinking age has resulted in substantially lower alcohol-related crash rates involving teenagers (sig.).<br>- Crashes increased in years in which Wisconsin's drinking age was lower than those of its neighbors, suggesting that "border hopping" resulted from interjurisdictional policy differences (sig.). Counties with moderate border traffic and interstate drinking age differentials have substantially more alcohol-related crashes than other counties, all else constant.	- A uniform, 21-year drinking age does, as critics argue, reduce state-level autonomy in determining alcohol policy.<br>- In addition, to the extent that a large percentage of teenagers do not drink and drive, increasing the drinking age to 21 does present a dilemma with respect to fairness. Forbidding all teenagers from drinking may indeed penalize many for the actions of a few.	**INCLUDE.**<br><br>-

[84]	First author, year, country	General aim	Study design	The policy measure(s) or intervention(s) at hand	Measured policy effects on MLDA	Reflection on policy and other occurrences by authors	Comments
	Lovenheim, 2010, USA.	An important but unexamined policy parameter is the degree to which cross-state differences in MLDAs induce teenage drunk driving. Most of the research addresses the effect of raising (in a majority of states) the drinking age to 21, while little attention has been paid to the equalization of the drinking ages. This paper adds to the literature by examining the effect of MLDA evasion across states with different alcohol restrictions from 1977 to 2002.	We use Geographic Information System (GIS) software to match with each U.S. county the closest locality in which an 18, 19, or 20-year-old legally can purchase alcohol and measure the population-weighted average distance from the county to that locality. Then, using data from FARS covering 1977–2002, which contains information on every fatal accident in the United States, we show that accidents involving only older drivers vary systematically with MLDA changes and with the distance to lower-MLDA borders. This variation suggests a difference-in-difference methodology is necessary to control for spurious fatal accident variation that is correlated with the timing of MLDA increases. We then estimate such a difference-in-difference model, which identifies how the likelihood that an 18, 19 or 20-year-old driver is involved in a fatal accident relative to older drivers varies with MLDA law changes and distance to lower-MLDA borders (using a longitudinal design).	Changes in MLDA between 1977 to 1988, and the equalization from 1988 onwards.<br><br>Controlling for:<br>- 0.08 per se laws.<br>- 0.02 zero tolerance laws.<br>- primary/secondary seatbelt law.<br>- BAC Law.<br>- Beer Tax.	- The effect of restricting alcohol by raising the MLDA locally increases the likelihood that an 18 or 19-year-old (but not a 20-year- old) driver is involved in a fatal accident (relative to all drivers over 25 years old) caused by the proximity of lower MLDAs.<br>- For counties more than 25 miles from a lower MLDA border, raising the drinking age within a state has a negative and statistically significant effect on the likelihood that a teenage driver is involved in a fatal accident. Thus, for countries more than 25 miles from a lower MLDA border, the results were consistent with previous literature that MLDA restrictions are effective in reducing accident fatalities.<br>- The effect of changes in the MLDA is quite heterogeneous across states, depending on the fraction of a state's population that need not travel far to reach a state with a lower MLDA.	Our results suggest that, by ignoring MLDA evasion, previous studies have underestimated the total effect of MLDA increases on teenage drunk driving. Unequal policies across unmonitored borders can induce the very behaviors the restrictions are meant to eliminate.	**INCLUDE.**<br><br>- Data from the Fatal Accident Reporting System (FARS) were used.

[87]	**First author, year, country**	**General aim**	**Study design**	**The policy measure(s) or intervention(s) at hand**	**Measured policy effects on MLDA**	**Reflection on policy and other occurrences by authors**	**Comments**
	Ponicki, 2007, USA.	The current study tests explicitly for interdependence between the impacts of MLDA and beer taxes in models of traffic fatalities. It is anticipated that the impacts of MLDA will diminish as one looks at age groups more distant to the 18- to 20-year olds more directly affected by these laws. The current study thus compares the interacted MLDA and tax effects across various youth and adult age groups.	The interdependence between the impacts of MLDA and taxes is investigated using a panel of 48 US states over the period 1975 to 2001. This is accomplished by introducing a multiplicative interaction term between MLDA and beer tax rates. All age-group–specific regression models control for numerous other variables previously shown to affect vehicle fatalities, as well as fixed effects to account for unexplained cross-sectional and time-series variation. The dependent variable in each model is the number of age-specific vehicle-occupant fatalities occurring in a state divided by the number (in thousands) of state residents of the relevant age group.	The current analyses concentrate on 2 forms of limitations on availability that have been shown to affect youth traffic fatalities: minimum legal drinking age (MLDA) laws and beer taxes.	- The present analyses indicate that traffic fatalities among those aged 18 to 20 can be effectively reduced by elevating the MLDA and by increasing beer tax rates (i.e., fatalities decline with higher MLDA and beer tax rates have a negative impact on youth fatalities) and MLDA has the largest and most-significant impact on fatalities aged 18 to 20.<br>- However, the direct negative impact of the beer tax variable on youth fatalities drops by half and is only marginally significant when the interaction term is introduced.<br>- The variable measuring the interaction between MLDA and beer taxation has a significant impact in the anticipated negative direction, indicating that each limitation on alcohol availability has a smaller impact on fatalities as the other form of availability is more restricted.<br>- As expected, a given change in MLDA causes a larger proportional change in fatalities when beer taxes are low than when they are high.	- These findings suggest that a community's expected benefit from a proposed limitation on alcohol availability depends on its current regulatory environment. Specifically, communities with relatively strong existing policies might expect smaller impacts than suggested by prior research, while places with weak current regulations might expect larger benefits from the same policy initiative.<br>- Higher MLDA are responsible for a smaller impact of beer taxes on youth drinking and fatalities, and strengthening drunken driving policies may have reduced the marginal impact of taxes and prices, which could explain the failure of many recent US studies to find significant tax effects on traffic crashes.<br>- The current study's findings suggest that the effectiveness of any given policy will vary over time and place, and more specifically will be more or less effective based on how much other regulation a community has (i.e., the current analyses indicate that local characteristics must be taken into account when predicting the impact of a proposed policy change).	**INCLUDE.**<br><br>- Data is derived from the annual micro-data sets of the Fatal Accident Reporting System (FARS).

[92]	**First author, year, country**	**General aim**	**Study design**	**The policy measure(s) or intervention(s) at hand**	**Measured policy effects on MLDA**	**Reflection on policy and other occurrences by authors**	**Comments**
	Dumochel, 1987, USA.	The present study was undertaken to assess longer-term effects of raising the alcohol purchase age. It has the advantage of including the experience of additional states that have enacted such legislation.	We compared changes in fatal crash involvement among affected drivers before and after the age changes with the experience of drivers not affected by the age change in those same states. The fatal crash involvements among affected drivers were also compared with those of same-age and other-age drivers in states that did not change their laws in the years 1975-84. The statistical analysis used produces regression coefficients that estimate the proportional reduction in driver fatal crash involvement rates associated with the prohibition of alcohol from drivers in particular state-age-year combinations.	The data available for this study include the years 1975-84, and it was possible to study twenty-six states that changed their laws during this period.	- Based on the 87,153 nighttime driver fatal crash involvements that occurred during 1975-84, raising the minimum legal alcohol purchase age was estimated to produce a 13 percent reduction in nighttime driver fatal crash involvements (sig.).<br>- In states with several years of experience with the raised purchase age law, no significant differences in the effects of the age change were observed after the first years of the change.<br>- The effect of the hazardousness of the first year of legal alcohol purchase was negligible and after a rerun, nonsignificant.	One possible interpretation of these results is that the law changes had the effect of reducing fatality rates not only for drivers whose legal ability to purchase alcohol is affected by the laws but for younger and older drivers (including "beginning" drinkers) as well.	**INCLUDE.**<br><br>- Fatal Accident Reporting System (FARS) data is used, based on drivers aged sixteen through twenty-four who were in crashes in which someone was killed during 1975-84 in 48 states in the US.<br>- Population estimates for each state were obtained from the U.S. Bureau of the Census.

[44]	First author, year, country	General aim	Study design	The policy measure(s) or intervention(s) at hand	Measured policy effects on MLDA	Reflection on policy and other occurrences by authors	Comments
	Hingson, 1983, USA.	- This paper examines the impact of raising the drinking age on teenage drinking, driving after drinking, and non-fatal accident involvement in Massachusetts prior to the law's enactment and twice at yearly intervals after the law enactment.<br>- Massachusetts was compared with New York State, who retained an 18-year-old drinking age.	- Random telephone surveys with approximately 1,000 16-19-year-olds in each state were undertaken prior to the law's enactment and twice at yearly intervals after the law, to assess the law's impact on teenage drinking, driving after drinking, and non-fatal accident involvement.<br>- Respondents were asked where they most often obtained their alcohol (e.g., liquor/grocery store, bar/club, at home) and where they drank > 5 consumptions in the last month (e.g., party, bar).<br>- Drinking was measured asking for 'any drinking' in the last month and 'drinking 6+ drinks at one time' in the last month.<br>- Log-linear analysis was used on the survey data.<br>- Fatal crash data reported to the US Department of Transportation by each state from 1976-1981 were also analyzed.<br>- To assess law enforcement practices and problems, interviews were conducted with over 50 Massachusetts police officers representing all levels of command in urban, rural, and suburban jurisdictions.	- Legislation raising the legal drinking age in Massachusetts from 18 to 20 in 1979.<br>- At the time Massachusetts raised its legal drinking age from 18 to 20, the two states had similar laws regarding age of driving licensure and penalties for driving while intoxicated.	- After the law, the frequency of teenage drinking in bars and clubs and the percentage of teenagers reporting they most often obtained their alcohol in liquor stores and groceries dropped in Massachusetts compared to New York. (sig.).<br>- During the two years after the law, the proportion of Massachusetts teenagers who had someone else purchase alcohol for them or who most often obtained alcohol from their homes nearly doubled (sig.).<br>- The average daily consumption of alcohol in the 16-19-year-old age group during the two years after the law did not decline in Massachusetts compared to New York. Nor did teenagers report shifts to the use of other psychoactive drugs.<br>- After the law, the proportion of teenagers who reported driving after drinking heavily (six or more drinks) did not decline in either state. However, the frequency that teenagers reported they drove after any drinking declined significantly more in Massachusetts.<br>- The three methods of statistical analysis indicated no significant difference between the two states in the overall teenage fatal accident trends (not sig.).<br>- The frequency with which teenagers were arrested for driving under the influence did not significantly change in Massachusetts during the first year after the law compared to the previous two years.<br>- Enforcement of the law focusing on the sellers was minimal and sporadic.<br>- The intensity of enforcement varied widely from community to community.<br>- The reasons most often cited for the variability in enforcement of the law among communities across the state was the lack of personnel and competing priorities, particularly in some high crime inner-city jurisdictions. Moreover, many officers did not perceive teenage purchasing of alcohol or drinking per se as a sufficiently serious crime to stigmatize<br>Juveniles by putting an arrest on their records.	- The study examined the first two years following enactment of the law. During this time period, the 18 and 19-year-old age groups who had previously been allowed to drink had that privilege revoked. One could hypothesize that the previous drinking habits of this group would be resistant to change.<br>- The state's law provides a symbolic statement to teenagers that its citizens disapprove of their drinking, and fears the accidents they may cause when they drive after drinking. The study results prompt us to ask whether the law could have had a greater impact among all Massachusetts teenagers if enforcement efforts were more consistent and if greater attention were paid to preventing the common practices of non-teenagers purchasing alcohol for teenagers or liquor outlets not requiring age identification. Without sufficient resources and coordination of enforcement efforts, those police who actively strive to enforce the law in one community may find their efforts negated by minimal enforcement in the next.<br>- Lack of community resources and variable willingness to enforce laws focused on teenagers raise questions about whether alternative strategies such as increased enforcement of the drunk driving and traffic safety laws aimed at all drivers, or requirements for safer cars and improved road design would yield greater reductions in nonfatal and fatal accidents both among teenagers and non-teenagers.	**INCLUDE.**<br><br>- In addition to telephone surveys, data from the Fatality Analysis Reporting System (FARS) was used.

[45]	First author, year, country	General aim	Study design	The policy measure(s) or intervention(s) at hand	Measured policy effects on MLDA	Reflection on policy and other occurrences by authors	Comments
	Vingilis, 1981, Canada.	This paper reports four types of data which are relevant to the study of the effects of the increase in the MLDA.	Data are from:<br>1) surveys of drinking and drinking problems among high school students (measuring: 1) alcohol use in the month prior, 2) feeling 'tight' at least once in the month prior, 3) 'drunk' at least once in the month prior, 4) 5 or more drinks on a singes occasion at least once in the month prior). Conducted in 1977 and 1979, stratified proportional sample in the province. Log linear analyses.<br>2) a study (surveys) of perceptions of vice-principals. Conducted in 1972 and 1980.<br>3) a trend analyses of young drinking offender charges; and<br>4) trend analyses of drinking-driving statistics and driver fatalities. Two types of drinking-driving statistics were obtained: monthly Ontario drinking-driving convictions form 1977 and 1979 for 16-21-year-olds and monthly Ontario accident fatalities from 1973 to 1979 for 16-21-year-olds. Time series analyses were used.	The increase of the MLDA in 1979 in Ontario (from 18 to 19).	1) the proportion of drinkers among 18-19-year-olds decreased from 1977 to 1979 (as well as a decrease in the proportion feeling 'tight'). Comparable effects were observed for younger drinkers (under 18) in the exact opposite direction.<br>2) The findings from this study indicate that the vice-principals have perceived either no change or less student drinking and alcohol-related problems at their school since the increase in legal drinking age (between 20 per cent and 30 per cent of the vice-principals reported a decrease in drink-related behaviors).<br>4) There were no significant differences between the pre-intervention and post-intervention time periods for both types of drinking-driving statistics.	- The results of the four studies indicate few statistically significant changes, however, these findings seem to tentatively suggest a minimal effect for 18-19-year-old high school students, but not for the regular (once a week or more) or younger drinkers.<br>- The fact that regular drinkers reported no changes in their drinking habits could partially explain why major changes were not observed for the more general methods of measurement, that is, the rates of accidents, charges and convictions and the perceptions of vice-principals.<br>- Additionally, a large proportion of Ontario youths are not in school and these non-students may be heavier drinkers. Conviction and fatality statistics are not sensitive enough measures, as it seems that too few high-accident-and-arrest-risk youths were changing their drinking patterns for an effect to emerge from these statistics.<br>- The minimal Ontario effect was expected, as it was felt that the one-year increase was not sufficient to cause a major impact on youthful drinking behavior.	INCLUDE.<br><br>- Data from the Fatal Accident Reporting System (FARS).<br><br>- Only 1, 2 and 4 approved criteria and are extracted.

[47]	First author, year, country	General aim	Study design	The policy measure(s) or intervention(s) at hand	Measured policy effects on MLDA	Reflection on policy and other occurrences by authors	Comments
	Schelleman-Offermans, 2017, the Netherlands.	The main aim of this study is to investigate the effect of the planned increase of the minimum legal age for the sale of alcohol in the Netherlands from 16 to 18 years old on the compliance of alcohol retailers (on- and off-premises) using 15-year-old mystery shoppers. In other words, the question is raised whether the compliance with the minimum legal age for selling alcoholic beverages via on-premise (sport bars, public bars, cafe's and disco's) and off-premise outlets (take-away restaurants, supermarkets, liquor stores, and alcohol home delivery services) significantly has increased for 15-year-olds after the minimum legal age was raised.	A total of 1770 alcohol purchase attempts by 15-year-old mystery shoppers were conducted in three independent cross-sectional Dutch representative samples of on- and off-premise alcohol outlets in 2013 (T0), 2014 (T1), and 2016 (T2). The effect of the policy change was estimated controlling for gender and age of the vendor. Univariate analyses (Chi-square) were conducted to explore changes in compliance one and two years after the increased minimum age was introduced. A single logistic regression analysis was conducted to estimate the effect of the policy change on compliance (no/yes) of alcohol sellers, controlling for the passage of time (T1, T2, with T0 as reference).	The increase from 16 to 18 years of the MLDA for the sale and purchase of all alcoholic beverages in the Netherlands in 2014.	- Results showed that vendors requested more frequently an ID (significant overall increase of 7.4% points after one year and 23.3% points after two years) of the 15-year-old mystery shoppers after the policy change.<br>- Mean compliance rates including all alcohol outlets increased significantly by 9.2% points after almost one year and 27.4% points after two years and 5 months compared with before the policy change, even after controlling for the gender and age of the vendor.<br>- Two years after the policy change, alcohol vendors were up to 3 times more likely to comply with the alcohol age limit policy.	- It can be concluded that it became more difficult for 15-year-old adolescents to purchase alcohol after the minimum legal age for alcohol was raised from 16 to 18 years and its effect on compliance seems to increase over time<br>- It might be naïve to believe that the effect found in this study can entirely be attributed to the implementation of the increased minimum legal age for alcohol. Several other changes occurred in the years preceding the raise of the minimum legal age, which might have contributed to the observed effect on compliance, such as the increased media attention or the changes in parental norms considering underage drinking (became stricter).<br>- Effects of the increase in minimum legal age may have been more pronounced if local alcohol policies and enforcement efforts would have been more adjusted to the alcohol policy changes; a process which may take longer than one year. Indeed, after two years, the effect of the new minimum legal age on compliance rates is much greater than after one year.<br>- A rise in the compliance rate was already present in the years preceding the introduction of the new minimum legal age. This perhaps signifies a process in which a lowering in the general acceptability of juvenile drinking already started before the increased minimum legal age was introduced and alcohol vendors might have been anticipating this formal legal change (i.e., policy endogeneity).	**INCLUDE.**<br><br>- In 2013, 1399 alcohol purchase attempts were conducted by 51 mystery shoppers, followed by 361 purchase attempts conducted by 19 different mystery shoppers in 2014, and 398 attempts conducted by 17 again different mystery shoppers in 2016 in on- and off-premise outlets.

[49]	First author, year, country	General aim	Study design	The policy measure(s) or intervention(s) at hand	Measured policy effects on MLDA	Reflection on policy and other occurrences by authors	Comments
	Yu, 1998, USA.	This study aims to examine: (1) the change of alcohol use and purchase patterns among the underaged immediately after the raise of the purchase age; (2) the long-term change in underage alcohol use and purchase patterns after the raise of the purchase age; and (3) the change in impaired driving practices among youth over time after the raise of the purchase age.	- Five telephone surveys were conducted with youths aged 16 to 24 in 10 sampled New York State counties in 1982, 1983, 1985, 1986, and 1996.<br>- Data were collected through telephone interviews from randomly selected New Yorkers (a series of five surveys were conducted in 1982, before the enactment of the 19-drinking age law; 1983, after the enactment of the 19-drinking age law, 1985, before the 21-drinking law; 1986, after the 21-drinking age law, and in 1996, a decade after the 21-purchase age was enacted).<br>- Two dependent variables are included: respondents' frequency of drinking in the past month and frequency of driving under the influence of alcohol in the past month.<br>- A three-stage stratified proportionate random sampling procedure was designed. The 57 non-New York City counties were stratified on the dimension of a county's young adult population (16-20 in 82 and 83, 16-24 in 85 and 86) and a county's alcohol crash involvement among those young adults.	New York state raised the legal alcohol drinking age twice in the 1980's, from 18 to 19 in 1982 and from 19 to 21 in 1985.	- Analysis of the self-reported data showed that, 10 years after the enactment of the 21-drinking age law, alcohol use among 18-, 19-, and 20-year-olds decreased by up to 58% (sig.).<br>- Frequent heavy weekend drinking was reduced by 53% for 16-year-olds and 43% for 17-year-olds between 1982 and 1996 (sig.).<br>- Over the following 10 years, the prevalence of self-reported alcohol use increased slightly to 73%, representing only a 1% decline from the 1985 rate for respondents who were 21 and older.<br>- Alcohol purchase rates of 19- and 20-year-olds were reduced by -70% from 1985 to 1996 (sig.).<br>- From 1982 (before the 19 law) to 1996, the impaired driving rates for 18-year-olds decreased by 84%.<br>- Although impaired driving rates declined over the survey years for each age group, -25% of all underage respondents in 1996 reported that they had ridden in a vehicle with an impaired driver.<br>- Between 1982 and 1983, when the purchase age was raised to 19, the perceived parental approval of alcohol use for the 18-year-old respondents dropped from 69% to 42%. Between 1985 and 1986, when the purchase age was raised to 21, the perceived parental approval rates changed from 68% to 48% for 19-year-olds and from 78% to 56% for 20-year-olds.<br>- In 1996, 69%, 76%, 77%, 82% and 80%, respectively, of the 16-, 17-, 18-, 19-, and 20-year-old respondents thought their peers would approve of their drinking.	- Both the 19 and 21 purchase age laws had immediate impact on alcohol purchase and use by the targeted youth groups. A decade later, the effectiveness of the 21-purchase age law continues.<br>- Analyses of 21-to 24-year-old respondents, who were not affected by the change in the law, showed that alcohol purchase and use rates did not significantly decline from 1985 to 1996. This finding provides further evidence that the purchase age laws tend to have a distinctive impact on the age groups which the laws specifically target.<br>- Weekend drinking tends to be one of the most important factors in youth highway crashes. Findings indicate that weekend drinking and heavy weekend drinking decreased, however, many underage respondents indicated drinking alcohol away from their own home, most commonly at friends' houses. After the establishment of the higher alcohol purchase ages, when youths cannot use alcohol at home in the presence of their parents, their alternative drinking location would be their friends' houses where parents may be absent.<br>- Continued enforcement of the 21-purchase age law will maximize its effect in reducing underage purchase and use of alcohol. Enforcement efforts may emphasize parental involvement in media campaigns.<br>- Anti-drunk driving campaigns, such as "friends don't let friends drive drunk," should not only target adult party-bar goers, but also target underage youths. "Zero Tolerance" laws, which reduce the illegal blood alcohol concentration level to 0.02 for drivers under the age of 21 and which have been enacted in 45 states and the District of Columbia, will also provide additional leverage to enforce the 21-purchase age law.	**INCLUDE.**<br><br>- The New York State Youth Alcohol Survey.<br>- New York State Department of Motor Vehicles records.<br><br>- Follow-up on study [48], using comparable data and methods, only now measuring long-time changes.<br><br>- Unclear about the significance of effects.

[50]	**First author, year, country**	**General aim**	**Study design**	**The policy measure(s) or intervention(s) at hand**	**Measured policy effects on MLDA**	**Reflection on policy and other occurrences by authors**	**Comments**
	Sannen, 2014, the Netherlands.	The aim of this exploration is to gain more insight into possible shifts in alcohol- and/or drug use among 16- and 17-year-olds after the change in the minimum legal drinking age.	- In order to obtain a quick scope of the situation, prevention workers of thirteen regular institutions for addiction-care in the Netherlands were consulted.<br>- In the summer of 2014, a number of questions were submitted to 23 contacts at these institutions.<br>- At that time, the change in the minimum legal drinking age was in force for more than six months.	The minimum legal drinking age in the Netherlands was changed on January 2014 from 16 to 18 years. Vendors are no longer allowed to sell alcohol to individuals younger than 18 years old, and individuals under 18 are punishable by law if they carry alcohol in publicly accessible places.	- Prevention workers generally do not see signs of increased illicit drug use among 16 and 17-year-olds after the change in legislation.<br>- If they see changes in illicit drug use, these changes are not always associated with the increase in the alcohol age from 16 to 18 years.<br>- The signals that may indicate a shift in alcohol or illicit drug use among 16 and 17-year-olds after the change in legislation are usually concerning very specific groups of young people, limited in size.<br>- Prevention workers do not expect a massive shift in the future either. Reasons for this are that young people still are required an effort to obtain illicit drugs. Also, alcohol is still easily available for underage individuals in the Netherlands, and, for most young people, changing from alcohol to illicit drugs is a big change or transmission.	- Respondents only observe few desirable effects of the increase of the minimum legal drinking age in the Netherlands. They see that 16- and 17-year-olds continue to drink because it is still easy to obtain alcohol when going out, for example obtaining drinks from older friends.<br>- According to the prevention workers, a significant proportion of 16- and 17-year-olds engage in drinking more out of sight, on private property. Parents allow and facilitate this by providing accommodations and alcohol, because they do not support the change in legislation and because they are afraid that their children will go out and drink on the street.	**INCLUDE.**<br><br>-

[51]	First author, year, country	General aim	Study design	The policy measure(s) or intervention(s) at hand	Measured policy effects on MLDA	Reflection on policy and other occurrences by authors	Comments
	Hingson, 1985, USA.	This study explores whether, when Massachusetts raised its legal drinking age from 18 to 20 in early 1979, significant declines occurred in that state in 1) teenage drinking and in turn, 2) homicide rates, 3) suicide rates, and 4) deaths from nontraffic accidents in 1980, 1981 and 1982 relative to New York State, where the legal drinking age remained at 18 during that time.	- An anonymous random digit dialing telephone survey of 16 to 19-year-olds was conducted in Massachusetts and New York (control), prior to enactment of the law in 1979, asking teenagers about their personal characteristics, drinking practices (average drinks daily), procurement of alcohol, use of psychoactive drugs and possible experiences with police enforcement when obtaining alcohol. Twice and yearly intervals following the law were conducted (six waves for each state). Log linear analyses was used to test the impact of the law on dependent variables.<br>- Interviews were conducted with 50 Massachusetts police officers and inspectors in all levels of command and context, asking them about enforcement practices before and after the drinking age change.<br>- Arrest data from the Uniform Crime Reporting system were evaluated before and after the law change for both states.<br>- The number of fatalities from non-traffic accidents, suicides, and homicides were analyzed separately, collating data into pre- and post-law groups. The number of fatalities was fit to a log linear model.	Raise of the legal drinking age from 18 to 20 in early 1979 in Massachusetts.	- After the law, the frequency of teenage drinking in bars-clubs-restaurants and the percentage of teenagers reporting they most often obtained their alcohol in liquor stores and groceries dropped in Massachusetts compared with New York (sig.).<br>- One third attempting to purchase liquor indicated they were never asked for ID, 5% were stopped by the police just once, none were arrested the first year after the law.<br>- Two years after the law, the proportion of Massachusetts teenagers who had someone else purchase alcohol for them nearly doubled (sig.).<br>- The average daily consumption of alcohol in the target population (15 to 19-year-olds) two years after the law did not significantly decline in Massachusetts compared to New York.<br>- After the law change, arrests among the target population rose over 150% (for multiple alcohol-related offenses).<br>- The intensity of enforcement was varied between communities caused by lack of personnel and competing priorities (as the main reasons). Also, many officers did not perceive underage drinking or purchasing alcohol as a serious crime.<br>- During the three years after Massachusetts raised its drinking age, compared with New York, there were no significant changes among the target population in the number of deaths from: 1) accidental injury other than traffic accidents, 2) the number of suicides or 3) homicide deaths.	- This study found no reductions in non-motor vehicle accident deaths, homicides, or suicides relative to New York among 15 to 19-year-olds after Massachusetts raised its drinking age from 18 to 20.<br>- It is possible that some types of nontraffic accidents, homicides, and suicide are also more likely than others to involve alcohol, but the possible relations are not well established and hence the specific subcategories of violent death are not monitored over time.<br>- Despite similarities between states, it is possible that one of the two experienced a change that could confound a change in violent death rates.<br>- The actual contribution that drinking makes is quite small relative to other independent predictors of other causes of death.<br>- It is conceivable that changes in location of drinking (e.g., form bars to drinking at home) might reduce the frequency of nighttime travel and rates of fatal crashes.	INCLUDE.<br><br>- An anonymous random digit dialing telephone survey of 16 to 19-year-olds.<br>- Interviews with 50 Massachusetts police officers and inspectors.<br>- Arrest data from the Uniform Crime Reporting system.<br>- The number of fatalities from non-traffic accidents, suicides, and homicides.

[52]	First author, year, country	General aim	Study design	The policy measure(s) or intervention(s) at hand	Measured policy effects on MLDA	Reflection on policy and other occurrences by authors	Comments
	Grucza, 2009, USA.	To evaluate trends in the past 30-day prevalence of binge drinking by age, gender, and student-status among youth and young adults in the United States between 1979 and 2006.	- Data were analysed from twenty administrations of the National Survey on Drug Use and Health, calculating trends in relative risk for four different age groups, student status and race/ethnicity, stratified by gender and relative to the 24-34-year-old reference group.<br>- Binge drinking has been queried as the number of days in the past 30 days in which an individual has consumed five or more drinks on any one occasion. In the 1979 survey, individuals were asked about the largest number of drinks they had in any 1 day in the past 30 days. These questions were used to determine the prevalence of individuals who had drunk five drinks in a day, at least once in the past 30 days.<br>- Multistage probability sampling was used for all surveys.	The federally mandated transition to a uniform legal drinking age of 21 years, and other policy changes aimed at curbing underage drinking.	- Individuals younger than 20 years have experienced marked reductions in risk for binge drinking, suggesting that changes in the MLDA, as well as other policy changes and public health campaigns, have been successful.<br>- Countering the former trend, risk for binge drinking among girls and young women has been rising, with risk increasing faster for minorities than for whites.<br>- The reduction in risk for binge drinking among youths has not reached college students.	The reduction in binge drinking among the youths in general is likely to be at least partly attributable to the adoption of the uniform drinking age of 21 years. Other policy changes (e.g., zero-tolerance driving under the influence laws) may also have had effects but have not been as thoroughly studied as the MLDA and zero-tolerance policies.	**INCLUDE.**<br><br>- Data is used from the National Survey on Drug Use and Health (NSDUH) and the formerly known National Household Survey on Drug Abuse (NHSDA).

[93]	First author, year, country	General aim	Study design	The policy measure(s) or intervention(s) at hand	Measured policy effects on MLDA	Reflection on policy and other occurrences by authors	Comments
	Durant, 1993, USA.	- The purposes of this study are twofold: (1) to demonstrate how multiple interrupted time series with nonequivalent control variables can afford a more rigorous and statistically sophisticated approach to the study of policy design; (2) to assess the promise and pitfalls of the policy tools approach as a vehicle for developing mid-range theories of social regulatory policy.<br>- To these ends, we use traffic safety reform as a policy window for examining over time the independent comparative and conjoint impacts from 1975 through 1987 of three traffic safety reforms in Michigan.	We used interrupted time series analysis to estimate the effects of the various social regulatory tools. Because of seasonality and autocorrelation problems in each series, we employed Autoregressive Integrated Moving Average (ARIMA) analysis.	- Elevating the minimum-level drinking age (MLDA) from 18 to 21 in 1978.<br>- Increasing the certainty of penalty for driving while impaired by alcohol in 1983.<br>- Passing a mandatory seat belt law (MSBL) effective in 1985.	- To assess the actual impact of the three regulatory tools, controlling simultaneously for the effects of the others (plus the two control variables), we computed the estimates of intervention. Analysis revealed that neither the seat belt law nor miles driven exhibited statistically significant impacts on fatalities in any time series.<br>- After identifying the appropriate model and estimating it *for all drivers*, the impact of the MLDA and drinking-driving reforms appeared as statistically significant determinants of traffic fatalities (for drivers of all ages).<br>- Moreover, Michigan's MLDA demonstrated a surprisingly strong and long-term effect across all age groups.	- Most importantly, changing the MLDA was the first step Michigan took to improve highway safety. The MLDA was the first regulatory tool employed in Michigan, it passed many years before drinking-and-driving became a hot issue in other states, and it raised the minimum drinking age comprehensively rather than incrementally. Moreover, the MLDA's diminution of fatalities across all age categories and not just its younger target group is explained simply: young intoxicated drivers kill persons in all age groups, not just their own.<br>- To be most fully effective in reducing fatalities among young drivers, a high MLDA may have to be accompanied by effector tools which enhance the certainty of punishment afforded by regulators.	**INCLUDE.**<br><br>- Fatal Accident Reporting System (FARS) data is used from 1975 to 1987 in Michigan.

[95]	First author, year, country	General aim	Study design	The policy measure(s) or intervention(s) at hand	Measured policy effects on MLDA	Reflection on policy and other occurrences by authors	Comments
	Legge, 1990, USA.	To explore the impact of four types of policy interventions on traffic safety in New York State between 1975 and 1987.	- Autoregressive Integrated Moving Average (ARIMA) analysis with a "step" function is employed to measure the effect of each intervention.<br>- With regard to analyzing the effect of the DWI reform, single- vehicle fatal crashes involving male drivers at night will be compared to total daytime fatalities.	- The 28 November 1981 law which standardized and increased penalties for drinking and driving.<br>- The legislation which went into effect on 1 December 1982 raising the MLDA from 18 to 19 years of age.<br>- The mandatory seat belt law, effective 1 January 1985.<br>-The legislation which raised the MLDA from 19 to 21 years of age, effective 1 December 1985.	- The strongest impact was demonstrated by the 28 November 1981 DWI reform.<br>- While the effects of the age change laws were not significant (even when subdividing populations and time and day of the fatality), the impact of the seat belt law shows some strength.	- The most significant conclusion which challenges the literature to date, is that a strong law against drinking and driving is not necessarily confined to a short-term effect.<br>- The success of the law is best explained by a combination of strong public support, rigorous law enforcement, and the financing provisions of STOP-DWI. The new law raised the certainty of both conviction and more rigorous financial punishment. In addition, the program is popular because local governments are given considerable liberty in the usage of collected fines, and hence became strong advocates of the law. Another reason why this reform triggered a more permanent change is that groups such as MADD and other alcohol-conscious groups helped to create a climate which placed potential gains to public safety over the benefits of social drinking.<br>- The most disappointing results of the study concern the MLDA reforms. One possible explanation for the poor performance is New York's gradual approach to implementing change (in contrast to a state such as Michigan, which abruptly increased the drinking age from 18 to 21 in 1978). Another explanation could be that given the power of the DWI and seat belt reforms, it may have been difficult to reduce fatalities much further.	**INCLUDE.**<br><br>- Data from the Fatal Accident Reporting System (FARS) were used for measuring traffic fatalities.

[97]	First author, year, country	General aim	Study design	The policy measure(s) or intervention(s) at hand	Measured policy effects on MLDA	Reflection on policy and other occurrences by authors	Comments
	Grossman, 1986, USA.	- A primary purpose of this paper is to investigate the responsiveness of motor vehicle death rates of youths aged 15 through 24 to variations in the cost of beer as reflected by differences in state excise tax rates on beer.<br>- We also examined the effect of an increase in the legal drinking age on youth motor vehicle deaths.	The empirical research is based on a time series of state cross sections for the period from 1975 through 1981. Logit motor vehicle death rate regressions are obtained for three age groups: youths aged 15-17, youths aged 18-20, and youths aged 21-24.	- Real beer tax (sum of Federal and state excise taxes; price of alcohol).<br>- Beer legal drinking age.	- Negative and statistically significant real beer tax effects are obtained for youths aged 15 through 17, 18 through 20, and 21 through 24 (the elasticity of the death rate with respect to the real beer tax is -.09 for the youngest age group and -.17 for the other two age groups).<br>- Negative and statistically significant legal drinking age effects are obtained for youths aged 18 through 20<br>- Effects of taxes and MLDA are not affected by the inclusion of drinking sentiment (e.g., 'wet' counties permitting the sale of alcoholic beverages and religious background) proxies.	- That effects are not affected by drinking sentiment proxies means that the tax and MLDA effects emphasized here are not artifacts of the endogeneity of state laws and decision-making.<br>- If reductions in youth motor vehicle accident deaths are desired, both uniform MLDA of 21 and increase in the federal excise tax rate on beer are effective policies to accomplish this goal.<br>- Results also suggest that the tax policy may be more potent than the drinking age policy and may additionally reduce fatal crashes involving adults (as opposed to MLDA primarily focusing on youths).<br>- Excise tax hikes impose welfare costs on all segments of the population, MLDA is targeted at the group that accounts for a disproportionate share of motor vehicle accidents and deaths. However, enforcement and administrative costs associated with a uniform MLDA of 21 may exceed those associated with the tax policy.<br>- If substantial resources must be allocated to raising these probabilities (deterring offenses of drunken drivers), the excise tax policy may be preferable to or complementary with a system of large fines.	**INCLUDE.**<br><br>- Information on traffic mortality was obtained from the National Highway Traffic Safety Administration's Fatal Accident Reporting System (FARS).

[98]	**First author, year, country**	**General aim**	**Study design**	**The policy measure(s) or intervention(s) at hand**	**Measured policy effects on MLDA**	**Reflection on policy and other occurrences by authors**	**Comments**
	Saffer, 1986, USA.	The purpose of this paper is to estimate the responsiveness of youth motor vehicle fatality rates to increases in the legal drinking age and to variations in the cost of beer.	The data set employed is time series, from 1975 to 1981, of cross sections of the 48 contiguous states. Separate regressions for 15 to 17-year-olds 18 to 20-year-olds and 21 to 24-year-olds are presented (and a simultaneous estimation model is used to account for the endogeneity of the drinking age).	- Real beer tax (sum of Federal and state excise taxes; price of alcohol).<br>- Beer legal drinking age.	- The econometric results show that the drinking age and beer tax both have a significant influence on youth motor vehicle mortality.<br>- The drinking age is assumed to be endogenous in the methodology used to compute these estimates.	- The econometric results show that mortality has a significant causal effect on the drinking age and that ignoring the problem of endogeneity results in underestimation of the effects of this policy variable.	**INCLUDE.**<br><br>- Information on traffic mortality was obtained from the National Highway Traffic Safety Administration's Fatal Accident Reporting System (FARS).

[102]	First author, year, country	General aim	Study design	The policy measure(s) or intervention(s) at hand	Measured policy effects on MLDA	Reflection on policy and other occurrences by authors	Comments
	Colon, 1984, USA.	The relationship between the alcohol beverage purchase age and single-vehicle fatalities is examined.	- The 50 states and the District of Columbia were used in cross-sectional analyses for the year 1976. Two separate analyses were performed: a bivariate analysis that cross- tabulated the beverage purchase age and single-vehicle fatalities and a multiple regression analysis in which the single vehicle fatality rate was the dependent variable.	The MLDA in 50 states and the District of Columbia in the year 1976.	- Support was found for a positive association between the purchase age and single motor vehicle fatalities. Since the purchase age variable and the purchase age concept are inversely related (i.e., as the purchase age is reduced, the drinking age population increases), the hypothesis of a positive relationship between purchase age and traffic fatalities is supported.	- An alternative explanation for this finding is that minors residing in a locality with a high purchase age drive to adjacent localities where they can purchase alcoholic beverages legally.<br>- It appears that the present state of affairs, where each locality sets its own purchase age independently, exacerbates highway fatalities. A uniform national purchase age is suggested as a means of dealing with this problem.	**INCLUDE.**<br><br>**-**

[104]	**First author, year, country**	**General aim**	**Study design**	**The policy measure(s) or intervention(s) at hand**	**Measured policy effects on MLDA**	**Reflection on policy and other occurrences by authors**	**Comments**
	Saffer, 1987, USA.	The purpose of this paper is to estimate the responsiveness of youth motor vehicle fatality rates to increases in the MLDA and to variations in the cost of beer.	- This study uses time-series data of 48 state cross-sections from 1975 through 1981 and estimates separate fatality regressions for youths fifteen to seventeen, eighteen to twenty and twenty-one to twenty-four.	- The study employs a sample period in which states raised their MLDA.<br>- Real beer tax.	- The results for the single equation mortality model show that the real beer tax and the MLDA are negative and significant on motor vehicle death rates *for youths eighteen to twenty.*<br>- Using comparable analysis, the MLDA is significant in the *fifteen- to seventeen-year-old group* as well.<br>- For *twenty-one to twenty-four-year-olds*, the higher MLDA is insignificant.<br>- For the abovementioned two age groups, the real beer tax is negative and significant.	- The econometric results show that mortality has a significant causal effect on the MLDA and that ignoring the problem of endogeneity results in underestimation of the effects of this policy variable (the empirical evidence supports the endogeneity assumption).<br>- It should be noted that while MLDA and beer tax policies can reduce highway mortality, both policies also subject the innocent to punitive action. Economists have shown that the optimal method of deterring offenses is a program of certain and appropriate sanctions against the guilty. However, high cost of increasing the probability of apprehension could limit the desirability of a program of specific sanctions.	**INCLUDE.**<br><br>- Data from the Fatal Accident Reporting System (FARS).

[110]	First author, year, country	General aim	Study design	The policy measure(s) or intervention(s) at hand	Measured policy effects on MLDA	Reflection on policy and other occurrences by authors	Comments
	Jones, 1992, USA.	This study examined the effect of legal drinking age (LDA) on fatal injuries (regarding motor vehicle drivers, motorcyclists, pedestrians, unintentional injuries, excluding motor vehicles, suicides and homicides) in persons aged 15 to 24 years in the United States between 1979 and 1984.	Effects on pre-LDA teens, adolescents targeted by LDA, initiation at LDA, and post-LDA drinking experience were assessed using logistic regression analyses.	All 50 states and the District of Columbia were studied for the years 1979 through 1984.	- The results of this study suggest that the net benefit of an LDA of 21 is found not only among motor vehicle drivers, but among other categories of violent death as well (sig. for some).<br>- The findings indicate that raising the LDA may have three effects (sig. for some): 1) delaying legal access to alcohol among pre-LDA adolescents, 2) preventing traumatic deaths that occur with legal access (a 3.9% increase in death rate among persons of a given age who can drink legally) and 3) delaying the onset of heavy drinking and associated fatal injuries that can occur with experience.	-	**INCLUDE.**<br><br>- Mortality data were collected from the National Center for Health Statistics.

[115]	First author, year, country	General aim	Study design	The policy measure(s) or intervention(s) at hand	Measured policy effects on MLDA	Reflection on policy and other occurrences by authors	Comments
	Fertig, 2009, USA.	This study examines the consequences of minimum legal drinking age (MLDA) laws on birth outcomes.	- To estimate the effect of MLDA laws on birth outcomes, we use a linear probability model which includes year–month of conception fixed effects, state fixed effects, maternal age at conception fixed effects, and state-specific time trends.<br>- Women aged 21–24 at the time of conception are treated as a control group to account for unobserved time-varying state-level factors which could affect infant health.	Increases in state minimum drinking ages over the 1980s (using birth years 1978–1989 to create a sample of births conceived in the years 1978–1988 to mothers aged 14–24 at the time of conception).	- Changes in the minimum legal drinking age (MLDA) are related to prenatal drinking when the drinking age is 18 (sig.).<br>- A drinking age of 18 is associated with adverse outcomes among births to young mothers (particularly strong for black mothers), including higher incidences of low birth weight (sig.) and premature birth (sig.), but not congenital anomalies.<br>- Parental characteristics are related to MLDA laws, a lower drinking age is associated with lower educational levels for white women (sig.) and the absence of paternal information on the birth certificate for black women (sig.).	- Alcohol policy that more effectively curtailed drinking, or the risky behaviors associated with it, might hold greater promise for infant health.<br>- The evidence suggests that lenient drinking laws generate poor birth outcomes in part because they increase the number of unplanned pregnancies.<br>- Our results also suggest that stricter alcohol policies may have positive unintended consequences—benefits for the well-being of a generation beyond those directly targeted.	**INCLUDE.**<br><br>- Data from the National Longitudinal Survey of Youth (NLSY) is used.<br>- Data is used from the National Vital Statistics (NVS) for the years 1978–1988.<br>- Data on MLDA laws come from the Distilled Spirits Council of the U.S. (DISCUS)

[116]	First author, year, country	General aim	Study design	The policy measure(s) or intervention(s) at hand	Measured policy effects on MLDA	Reflection on policy and other occurrences by authors	Comments
	Joksch, 1993, USA.	This study examined whether limiting legal access to alcohol for certain age groups affected the commission of selected crimes by individuals in those age groups.	- Arrest data from the Uniform Crime Reports and FARS-data were analyzed in relation to changes in the drinking age in the range 18 to 21 years.<br>- The analysis had three parts: 1) relating blood alcohol of drivers in fatal accidents to changes in the drinking age, 2) relating arrests to changes in the drinking age and 3) relating changes in blood alcohol to changes in arrests.	Changes in the drinking age in the range 18 to 21 years (only states that raised the drinking age between 1981 and 1986) were studied (18 states), in addition, comparison states with no changes in their drinking ages were used (13 states).	- Reduced alcohol involvement in fatal accidents was, overall, significantly related to a raised drinking age.<br>- The corresponding analyses of arrest data showed declines on the order of 10 per-<br>cent related to raised drinking ages for vandalism and for disorderly conduct, but not for violent crimes.<br>- This study found a strong suggestion that among the four crime types, the effect of the raised drinking age increased with decreasing severity of the crime.<br>- This study found no indication of the expected relationship between changes in the drinking indicator and changes in the crime indicators (however, standard errors were very large).	The effects of raising the drinking age may differ among the states due to differences in the distribution systems for alcoholic beverages, varying degrees of enforcement, neigh-boring states with lower drinking ages, and more subtle socioeconomic factors.	**INCLUDE.**<br><br>- Arrest data from the Uniform Crime Reports were examined.<br><br>- Fatal (motor vehicle) Accident Reporting System (FARS) was studied.

[117]	First author, year, country	General aim	Study design	The policy measure(s) or intervention(s) at hand	Measured policy effects on MLDA	Reflection on policy and other occurrences by authors	Comments
	Norberg, 2009, USA.	We use a ''natural experiment'' study design to compare the 12-month prevalence of alcohol and substance use disorders among adult subjects exposed to different minimum legal drinking age laws in the 1970s and 1980s.	- The sample pools 33,869 respondents born in the United States from 1948 to 1970, and drawn from 2 nationally representative cross-sectional surveys. Analyses control for state and birth year fixed effects, age at assessment, alcohol taxes, and other demographic and social background factors.<br>- The main outcome measures for the present study were binary variables reflecting whether the respondent met DSM-IV criteria for alcohol, marijuana, or other illegal substance abuse or dependence within the previous 12 months.<br>- Logistic and probit regression models were used to estimate the relative odds or relative risk of a past-year alcohol or drug use disorder or cross-state migration among ''exposed'' and ''unexposed'' subjects, and to investigate the possibility of differences in effect estimates across demographic groups.	- Changes in minimum legal drinking age (MLDA) laws in the United States during the 1970s and 1980s.<br>- Control for state beer taxes in the year that the respondent turned 18.	- Adults who had been legally allowed to purchase alcohol before age 21 were more likely to meet criteria for an alcohol use disorder or another drug use disorder within the past year, even among subjects in their 40s and 50s (exposure to a younger legal purchase age is associated with more than a 30% increase in the odds of a past-year alcohol use disorder) (sig.).<br>- The effect estimates were little changed by inclusion of age of initiation as a potential mediating variable in the multiple regression models.<br>- There were no significant differences in effect estimates by respondent gender, black or Hispanic ethnicity, age, birth cohort, or self-reported age of initiation of regular drinking.	- The MLDA effects do not seem to be working through age of drinking initiation: although MLDA exposures did predict age of onset of regular and weekly drinking, the effect estimates for alcohol and substance use disorders were little changed in regression models that also included lifetime abstention status and age of initiation, and were similar and separately significant among subjects who had already started to drink by the age of 16 and among those who had not. Although age of onset may indeed be a causal risk factor for later substance use disorders, these results suggest that the long-term effects of MLDA exposures on harmful drinking may work through other aspects of late adolescent drinking, such as the intensity or patterning of drinking.<br>- It is plausible that the effects of MLDA laws on patterns of drinking among early onset drinkers could be explained by the influence of MLDA laws on the behavior of their more law-abiding peers. For example, if young adults prefer to drink with friends than to drink alone, then even among respondents who had already begun to drink before age 18, a more restrictive purchase age could influence the frequency, activities, and social composition of the encounters around which people form enduring social relationships.<br>- MLDA laws were not the only social processes that might have affected lifetime patterns of alcohol and other substance use for cohorts coming of age in this period. If the apparent MLDA effects are not attributable to changing drinking age laws themselves, then they are due to an environmental factor closely tied to the timing of these changing laws.<br>- We have not tried to account for cross-state differences in law enforcement or ease of access to alcohol in neighboring states, and we did not distinguish between furnishing, purchase, possession, and consumption.	**INCLUDE.**<br><br>- The 1991 National Longitudinal Alcohol Epidemiological Survey (NLAES)<br>- The 2001 National Epidemiological Study of Alcohol and Related Conditions.

[118]	First author, year, country	General aim	Study design	The policy measure(s) or intervention(s) at hand	Measured policy effects on MLDA	Reflection on policy and other occurrences by authors	Comments
	Plunk, 2015, USA.	We used MLDA changes during the 1970s and 1980s as a natural experiment to investigate how underage exposure to permissive MLDA affected high school dropout.	- MLDA exposure was added to two data sets: (a) the 5% public use microdata samples of the 1990 and 2000 censuses (n = 3,671,075), and (b) a combined data set based on the 1991–1992 National Longitudinal Alcohol Epidemiological Survey (NLAES) and the 2001–2002 National Epidemiological Survey on Alcohol and Related Conditions (NESARC; n = 16,331).<br>- We used logistic regression to model different thresholds of MLDA on high school dropout. We also estimated models conditioned on demographic variables and familial risk of developing alcohol problems.	The period of greatest change in MLDA (1978–1987).	- Only the MLDA of 18 predicted high school dropouts (sig.).<br>- Exposure was associated with 4% and 13% higher odds of high school dropout for the census and NLAES/NESARC samples, respectively.<br>- The MLDA of 18 also promoted transitioning from regular to weekly drinking while of high school age, but only for those individuals with a history of parental alcohol problems (sig.).	- The most plausible way by which underage high school students were affected by the MLDA of 18 would be their 18-year-old peers, which suggests that permissive MLDA could have promoted high school environments similar to those we observe on college campuses today. Current proposals to lower the drinking age in response to risky underage college drinking would need to address the degree to which these behaviors would occur earlier at the high school level. The apparent differential effect based on predisposition for developing drinking problems also provides further evidence that policy can successfully affect drinking behavior in young adult populations characterized by high environmental and genetic risk.<br>- High school dropout is a complex phenomenon, and the data sets we used did not capture many relevant risk factors (e.g., individual-level factors including parental educational attainment, childhood experiences, and socioeconomic status; school- or district-level factors such as quality of instruction and local funding). As such, our estimates reflected the average effect of MLDA exposure while holding constant other unmeasured factors that might also have influenced educational attainment.	**INCLUDE.**<br><br>- The Integrated Public Use Microdata Series for the 1990 and 2000 decennial censuses.<br>- The 1991–1992 National Longitudinal Alcohol Epidemiological Survey (NLAES).<br>- The 2001–2002 National Epidemiological Survey on Alcohol and Related Conditions (NESARC).

[123]	**First author, year, country**	**General aim**	**Study design**	**The policy measure(s) or intervention(s) at hand**	**Measured policy effects on MLDA**	**Reflection on policy and other occurrences by authors**	**Comments**
	Roy, 1979, USA.	The objective of this DUIL (Driving Under the Influence of Liquor) study was to determine the age and sex distribution of drunk driver defendants in the state of Massachusetts, and to assess the incidence of simultaneous offenses by age. This study was undertaken to assess any shifts in age of defendants since the enactment	The Office of the Commissioner of Probation (OCP) analysed data based on court appearance records received from 70 probation departments state-wide from October 1979. This data was compared to records received from February to March, 1979. The OCP is unique in that all criminal and delinquency records statewide are centrally filed and stored in the OCP Central File. Only those records reflecting new charges for drunk driving were included in the samples.	Massachusetts legislation which raised the legal drinking age from 18 to 20 years in April 1979.	- No significant shifts in volume of drunk driver arrests have occurred as a result of the new legislation.<br>- When the October sample was compared to a similar sample in February, this DUIL study found a 26 percent increase in the number of teenagers (15-19 years of age) who were charged with driving under the influence of liquor (n=250 in February, n=316 in October). While teenagers comprised about 14 percent of the February DUIL defendants, they accounted for over 17 percent in October.<br>- Teenagers were found to have a higher than predicted frequency of multiple offenses, including: operating to endanger, use of a motor vehicle without authority, leaving the scene of an accident with property damage and personal injury, crimes against property and public order offenses.	- While one would have expected a sharp reduction in teenage drunk drivers after the change in legislation, this follow-up research found an increased number of arrests among people under 20 years of age. This may be due to: intensive police enforcement. However, it is also likely that some teenagers are probably not honoring the new law. They may be drinking more in cars inasmuch as they cannot legally drink in a tavern or bar.	**INCLUDE.**<br><br>- The (OCP) Central File.
